# Association of microsatellite pairs with segmental duplications in insect genomes

**DOI:** 10.1186/1471-2164-14-907

**Published:** 2013-12-21

**Authors:** Susanta K Behura, David W Severson

**Affiliations:** 1 Eck Institute for Global Health, Department of Biological Sciences, University of Notre Dame, Notre Dame, IN 46556, USA

**Keywords:** Segmental duplication, Genome dynamics, Microsatellite, Insect genomes, Duplication shadowing, Gene duplication

## Abstract

**Background:**

Segmental duplications (SDs), also known as low-copy repeats, are DNA sequences of length greater than 1 kb which are duplicated with a high degree of sequence identity (greater than 90%) causing instability in genomes. SDs are generally found in the genome as mosaic forms of duplicated sequences which are generated by a two-step process: first, multiple duplicated sequences are aggregated at specific genomic regions, and then, these primary duplications undergo multiple secondary duplications. However, the mechanism of how duplicated sequences are aggregated in the first place is not well understood.

**Results:**

By analyzing the distribution of microsatellite sequences among twenty insect species in a genome-wide manner it was found that pairs of microsatellites along with the intervening sequences were duplicated multiple times in each genome. They were found as low copy repeats or segmental duplications when the duplicated loci were greater than 1 kb in length and had greater than 90% sequence similarity. By performing a sliding-window genomic analysis for number of paired microsatellites and number of segmental duplications, it was observed that regions rich in repetitive paired microsatellites tend to get richer in segmental duplication suggesting a “rich-gets-richer” mode of aggregation of the duplicated loci in specific regions of the genome. Results further show that the relationship between number of paired microsatellites and segmental duplications among the species is independent of the known phylogeny suggesting that association of microsatellites with segmental duplications may be a species-specific evolutionary process. It was also observed that the repetitive microsatellite pairs are associated with gene duplications but those sequences are rarely retained in the orthologous genes between species. Although some of the duplicated sequences with microsatellites as termini were found within transposable elements (TEs) of *Drosophila*, most of the duplications are found in the TE-free and gene-free regions of the genome.

**Conclusion:**

The study clearly suggests that microsatellites are instrumental in extensive sequence duplications that may contribute to species-specific evolution of genome plasticity in insects.

## Background

Microsatellites are tandem repeats of simple sequences (usually consisting of motifs less than 6 bp long) which are found ubiquitously across eukaryotic genomes. Generally, microsatellite loci are assumed to be selectively neutral [1-3]. However, increasing evidence now suggests that microsatellites are associated with important roles in genome structure and evolution and are often subjected to selective pressure [4-10].

Moreover, non-random genomic distributions of microsatellites are well documented in eukaryotes [4,11-15]. As much as 25% of the microsatellites are localized close to each other, generally within 10 bp, in different eukaryotic genomes as found by Kofler *et al.*[14]. Furthermore, Kofler *et al.*[14] showed that these microsatellites are localized close to each other in the genomes at a higher frequency than expected under the assumption of random genomic distribution. In addition, simple sequence coding sequences are distributed differentially in the genomes as evident from analysis of 25 insect species [10]. Also, the Mouse Genome Sequencing Consortium, [16] has revealed that the ends of chromosome arms in mice are associated with higher density of microsatellites than other chromosomal regions. However, the functional and evolutionary relevance of non-random genomic distribution of microsatellites is poorly understood [6].

Studies have indicated possible association of microsatellites with segmentally duplicated sequences in some organisms [17-22]. Segmental duplications (SDs), also known as low-copy repeats, are generally defined as DNA sequences of length greater than 1 kb which are duplicated with high degree of sequence identity (>90%) [23]. SDs are important features of genomes as they may have functional consequences in genomic instability and diseases as evident in humans [17]. SDs are generally found in the genome as mosaic forms of duplicated sequences [19]. A two-step process generates such mosaic structures [24]. In the first step, multiple duplicated sequences are aggregated at specific genomic regions. In the second step, these primary duplications undergo multiple secondary duplications. However, the mechanism of how duplicated sequences are aggregated in the first place is not clear.

The present study is a systematic investigation to determine the distribution of microsatellite sequences in segmental duplications of different insect genomes (n=20). Although microsatellites are extensively used as genetic markers for population and ecological investigations of insects [9], the relationship of microsatellite sequences with segmental duplications has not been established in spite of availability of several insect genome sequences. Here we show that specific microsatellite pairs along with the intervening sequences are repeated with different frequencies in the genome and many of the low copy repeats of these loci are segmentally duplicated, henceforth called as microsatellite-associated SDs or mSDs. The results further show that these repeated microsatellite pairs (rMP) tend to aggregate at different genome regions along with the segmentally duplicated sequences suggesting a role for microsatellites in segmental duplications in insect genomes.

## Methods

### Insect genomes

A total of 20 insect genomes were investigated in this study. They included twelve *Drosophila* species [*D. melanogaster*, *D. simulans*, *D. sechellia*, *D. yakuba*, *D. erecta*, *D. ananassae*, *D. pseudoobscura*, *D. persimilis*, *D. willistoni*, *D. grimshawi*, *D. virilis*, *D. mojavensis*], three mosquito species [*Aedes aegypti* (*A. aegypti*), *Anopheles gambiae* (*A. gambiae*), *Culex quinquefasciatus* (*C. quinquefasciatus*)], the wasp (*Nasonia vitripennis*), the honey bee (*Apis mellifera*), the beetle (*Tribolium castaneum*), the silk worm (*Bombyx mori)* and the pea aphid (*Acyrthosiphon pisum*). The insect names have been abbreviated as the first letter of the genus followed by three letters of the species names throughout the text and the illustrations. The genome sequences of the twelve *Drosophila* species were downloaded from FlyBase (http://www.flybase.org). The genome assembly version for each of these was r1.3 except *D. melanogaster* (r5.27), *D. pseudoobscura* (r2.10) and for *D. virilis* (r1.2). The genome sequences of the three mosquitoes were downloaded from VectorBase (http://www.vectorbase.org). The *A. mellifera* genome sequence was downloaded from http://hymenopteragenome.org/. The Nasonia genome sequence (*N. vitripennis*_OGS_v1.2) was obtained from http://www.hgsc.bcm.tmc.edu. The aphid genome sequence was obtained from the AphidBase (http://www.aphidbase.com/aphidbase/). The silkworm genome sequences were obtained from the SilkDB (http://www.silkdb.org/silkdb/). The genome sequence of *T. castaneum* was obtained from the BeetleBase (http://beetlebase.org/).

### Non-random association of microsatellite pairs

The SciRoKo software [25] was used to identify the mono-, di-, tri-, tetra- and hexa-nucleotide simple sequence repeats (SSRs) or microsatellites in each genome. Both perfect and imperfect SSRs were detected by using the default parameters, with fixed penalty = 5 for mismatches between motif sequences. From the output files of SciRoKo (that generates microsatellite sequences, their position in the genome with start and end coordinates in chromosomes/scaffolds/supercontigs), distances between neighboring microsatellites were calculated in each species. When two microsatellites of the same repeat motifs had the same intervening distance at more than one location, they were counted as repetitive microsatellite pair (rMP). We assumed that presence of microsatellite pairs with the same motifs and same intervening distance at multiple locations in a genome was due to a random chance. Test of this null assumption was performed by calculating statistical significance of the hypergeometric probability as follows. First, the number of microsatellite pairs associated with the same intervening distance but different SSRs (n1) and the number of pairs associated with the same SSR pairs but with different intervening distances (n2) was determined in each genome. The total number of possible combinations for these two groups of SSR pairs was calculated as C(n, n1)* C(n, n2), where ‘n’ is the total number of microsatellites identified in the genome minus one (*i.e.* interventions between microsatellites), and ‘C’ represents the function of combination. Thus, C(n, n1) was calculated as the number of possibilities for choosing the ‘n1’ pairs from all the detected microsatellites in a genome. Of these, C(n1,n3) was calculated as the number of possibilities for choosing the same SSR pairs with the same intervening distance (n3). Thus, the number of combinations of the same SSR pairs having different intervening distance was calculated as C(n − n1, n2 − n3). From these, the cumulative probability of hypergeometric distribution of SSR pairs with the same intervening distance was calculated as $p=\sum_{1}^{n3-1}\left[\frac{\left[C\left(n,n1\right)\times C\left(n,n3\right)\times C\left(n-n1,n2-n3\right)\right]}{C\left(n,n1\right)*C\left(n,n2\right)}\right]$. Thus, 1-*p* value provided the statistical significance to reject or not to reject the null assumption. The multiple testing by Bonferroni correction method was conducted to adjust the individual *p* values. The threshold values less than 0.05 were considered statistically significant unless stated otherwise. The association was further tested in shuffled sequences of *A. aegypti* supercontigs. Here we assumed that the distribution of SSR pairs was independent of the sequence structure of the genomic sequences and hence sequence shuffling would not affect their distribution. To test this assumption, the supercontig sequences were shuffled and sampled (n = 1,000 sequences, each of 1 kb in length) using the *R* code ‘*ShuffleAndExtract*’ (http://tata-box-blog.blogspot.com/search/label/R). The sequences generated from three independent shuffling experiments were then analyzed separately for distribution of rMPs using hypergeometric tests as described above.

A canonical correlation test [26] was performed using the number of rMPs associated with different intervening distances (< 10 bp, ≥ 10 bp but < 100 bp, ≥ 100 bp but <1 kb, ≥ 1 kb but < 5 kb, ≥ 5 kb but < 10 kb and ≥ 10 kb but < 50 kb) among the 20 species. Euclidean distance measures were used in the correlation test and significance of correlation was determined by permutation test (n = 9,999 random) according to methods of Anderson and Willis [26].

### Intervening sequences of paired microsatellites

The intervening DNA sequences of the paired microsatellites were extracted using the coordinates of the microsatellite ends in the genome sequences by the *R* package SeqINR [27] or the GALAXY server (https://main.g2.bx.psu.edu/). The pair-wise alignments of duplicated sequences and the percent sequence identity of the alignments were performed using the *R* package ‘Biostrings’. The phylogenetic analyses were conducted using the Neighbor-Joining method in MEGA4 [28]. The evolutionary distances were computed using the maximum composite likelihood method [29] and were in the units of the number of base substitutions per site. The estimates of average evolutionary divergences between different groups of rMP loci (e.g. genic *versus* non-genic) were also calculated by MEGA4. All the sequence polymorphism analyses including calculation of total number of mutations, number of polymorphic sites, the average number of nucleotide differences among duplicated sequences, and significance of Tajima D statistics were conducted by DnaSP v 5.10 [30].

To determine the genomic distribution patterns of paired microsatellites, genome assemblies (where sequences have been assigned to chromosomes) were binned to determine the total number of rMPs and the total number of pairs as mSDs. The size and number of bins were variable depending upon the chromosome length but they were mostly in megabases (Mb). For example, the *A. gambiae* genome was binned as < 1 Mb, 1–5 Mb, 5–10 Mb, 10–20 Mb, 20–30 Mb, 30–40 Mb, 40–50 Mb and > 50 Mb for each chromosome. The total numbers of rMPs and the mSDs across individual windows were counted. The Spearman rank order correlation test was performed with the total number of rMPs and the total number of mSDs among the binned regions to determine if regions rich in paired microsatellites accumulated more segmental duplications than regions poor in paired microsatellites. The *p*-value < 0.05 was considered significant.

### Association of mSD sequences with gene duplications and transposons

The genomic positions of mSDs were used to determine if they were localized in genic regions. The start and end coordinates of annotated genes (both coding and non-coding) of each genome (Biomart dataset: Ensembl Metazoa 16) were used to determine if mSDs were localized within or overlapping with the genes. The gene ontology (GO) terms (downloaded from Biomart) of the genes associated with mSDs were analyzed. The rank orders of GO terms were used to determine the top ranking functions of these genes. The orthology and paralogy relationships of insect genes were obtained from Biomart (Metazoa) database. Based on sequence identity between paralogous copies, the nearly identical paralogs [31] were identified. The transposable element (TE) sequences annotated from *D. melanogaster* were analyzed to determine association of mSDs with transposable elements. The TE sequences were downloaded from ftp://ftp.flybase.net/genomes/aaa/transposable_elements/ReAS/v1/consensus_fasta/. The start and end coordinates of TEs in relation to mSDs were analyzed to determine TE-mSD associations.

## Results

### Identification of repetitive paired microsatellites

The microsatellite sequences were identified in a genome-wide manner among the 20 insect species that included three mosquitoes, twelve *Drosophila*, honey bee, silkworm, beetle, wasp and pea aphid. The total number of microsatellites and the genomic density (number of microsatellites per Mb of the genome) are shown in Table 1 for each species. Collectively, the microsatellite sequences represent 0.2–2.8% of the assembled genome size of these insects. The frequencies of mono-, di-, tri-, tetra-, penta- and hexa-nucleotide SSRs in each of the 20 genomes are provided in Additional file 1. The entire list of microsatellites and their positions in the genome assembly of all the 20 species are available upon request.

**Table 1:** Total counts and density (counts/Mb) of SSRs in each genome

**Species**	**Total counts**	**Counts/Mbp**
A.aeg	145952	105.46
A.gam	107379	393.19
A.mel	47626	811.41
A.pis	169601	365.28
B.mor	105270	218.96
C.qui	104998	181.33
D.ana	58789	254.51
D.ere	39857	260.99
D.gri	166045	828.29
D.mel	55092	326.5
D.moj	201983	1042.08
D.per	117292	622.65
D.pse	110050	720.51
D.sec	40855	245.26
D.sim	36690	266.2
D.vir	145470	706.07
D.wil	159532	677.37
D.yak	49301	297.54
N.vit	120015	336.43
T.cas	17899	85.13

The species names are listed as four-letter abbreviations.

**Figure 1:**
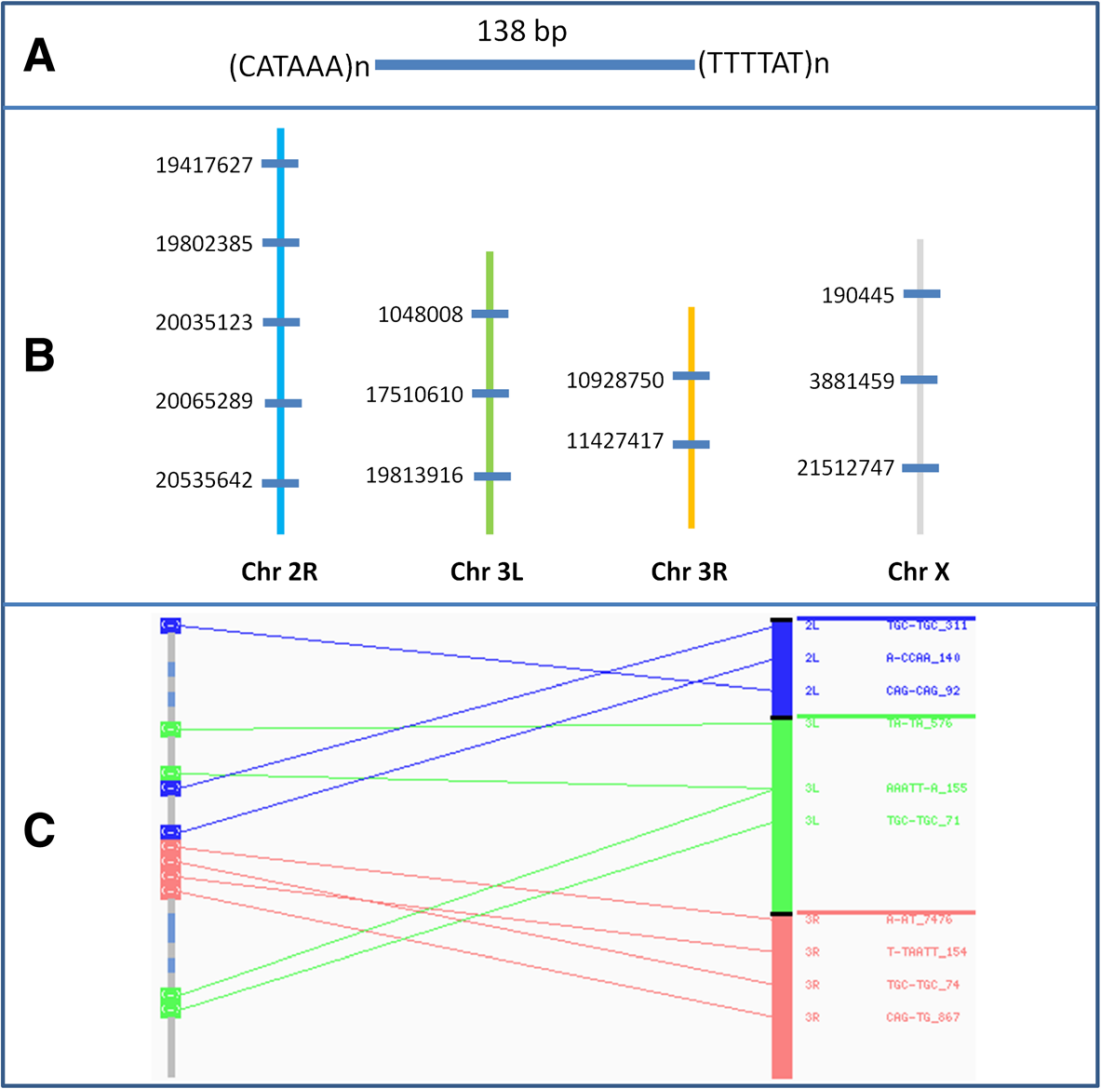
**Repetition of microsatellite pairs. A)** A repetitive microsatellite pair (rMP) of *D. melanogaster*. The pair of microsatellites (CATAA)n and (TTTTAT)n are localized at different locations in *D. melanogaster* chromosomes and in each location the distance between the two microsatellites is consistently 138 bp. **B)** The locations of the rMP are indicated by short horizontal lines (dark blue) by their mid-point positions in the chromosome. The chromosomes are shown as long vertical lines of different colors. **C)** A subset of rMPs and their relative distribution between chromosome X and chromosomes 2L (blue), 3L (green) and 3R (red). The corresponding locations of these rMPs in chromosome X are shown by different colors relative to the autosomes. The simple sequence motifs of the paired microsatellites and their intervening distances (in bp) are shown to the right end of the figure.

Based on the position of microsatellites in the genome, it was observed that specific microsatellite pairs are localized together with the same intervening distance at multiple locations in each genome (Figure 1). We identified these repetitive microsatellite pairs (rMPs) in each insect genome comprehensively. The list of all rMPs in the *D. melanogaster* genome is provided in Additional file 2, but the lists of rMPs of all the 20 genomes are available upon request. It was found that in each genome the frequency of rMPs varies depending upon the intervening distance of the paired microsatellites (Table 2). Table 2 shows that rMPs of 10 bp to 1 kb in size are predominant in all the genomes. It also shows that the rMPs with length greater than 5 kb are present only in specific species. Although some rMPs are up to 50 kb in length, none of the rMPs in any genome has an intervening distance greater than 50 kb. On the other hand, rMPs of smaller sizes (< 10 bp) were relatively high in frequency in these insects (Table 2).

**Table 2:** Frequency and inter-SSR distance of paired microsatellites in different insects

**Species**	**< 10 bp**	**10 bp −100 bp**	**100 bp −1 kb**	**1 kb -5 kb**	**5 kb - 10 kb**	**10 kb - 50 kb**	**50 kb - 100 kb**
A.aeg	779	1317	1523	804	165	56	0
A.gam	756	1035	3992	871	18	2	0
A.mel	492	594	1582	191	2	0	0
A.pis	172	917	831	0	40	0	0
B.mor	726	2065	1582	436	97	14	0
C.qui	866	2333	1054	519	54	2	0
D.ana	271	590	762	104	4	0	0
D.ere	108	195	198	47	2	0	0
D.gri	2998	5121	12952	792	0	0	0
D.mel	760	632	936	237	51	2	0
D.moj	4090	7218	12981	684	2	0	0
D.per	865	849	2681	182	2	0	0
D.pse	476	577	975	97	0	0	0
D.sec	291	647	1067	51	6	0	0
D.sim	352	389	151	8	2	0	0
D.vir	2922	4796	8353	618	10	0	0
D.wil	1246	2872	7282	532	4	0	0
D.yak	326	289	355	30	0	0	0
N.vit	3010	3487	14058	2717	40	2	0
T.cas	134	133	515	7	0	0	0

The intervening distance groups between the microsatellite pairs are shown as the heading (bp = base pairs and kb = kilobase). The distance groups exclude the upper boundary. Species names are four letter abbreviations.

### Non-random distribution of microsatellite pairs

**Figure 2:**
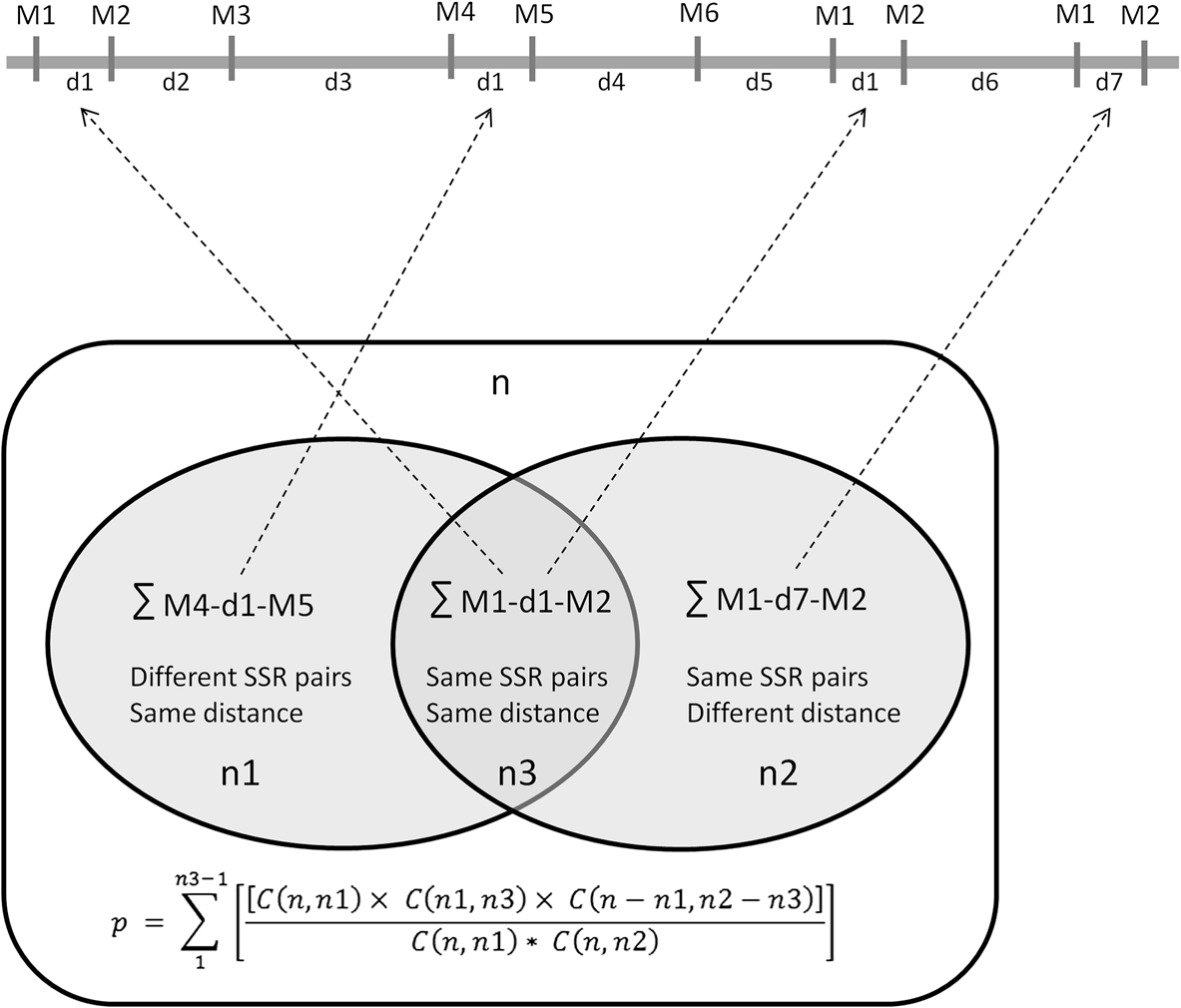
**Hypergeometric distribution of repetitive microsatellite pairs.** The grey horizontal line on the top shows hypothetical genomic locations of different microsatellites (M1 through M6) with intervening distance (d) shown in between. The microsatellite pair M1-M2 is localized at two positions where the intervening distance is the same (d1) at both locations. The M1-M2 pair is also located at another position but with a different intervening distance (d7). On the other hand, a different pair of microsatellites (M4 and M5) is found wherein the intervening distance is again d1. M3 and M6 are not associated with any repetitive microsatellite pair. Below that, the rectangular box shows all the neighboring pairs of microsatellites identified in the genome which is basically the total counts of microsatellites minus 1. The two circles within the box contain n1 and n2 microsatellite pairs respectively, where n1 is the total count of different SSR pairs but has the same distance d1 (i.e. M4-M5 pairs) and n2 is the total counts of the same SSR pairs (M1-M2) but with different intervening distance (d7). The overlap between the two circles represents the total number (n3) of SSR pairs with the same SSR sequences (M1-M2) as well as the same intervening distance (d1). The probability of obtaining these n3 numbers of repetitive pairs of M1-M2 is the hypergeometric distribution probability shown below the circles.

**Figure 3:**
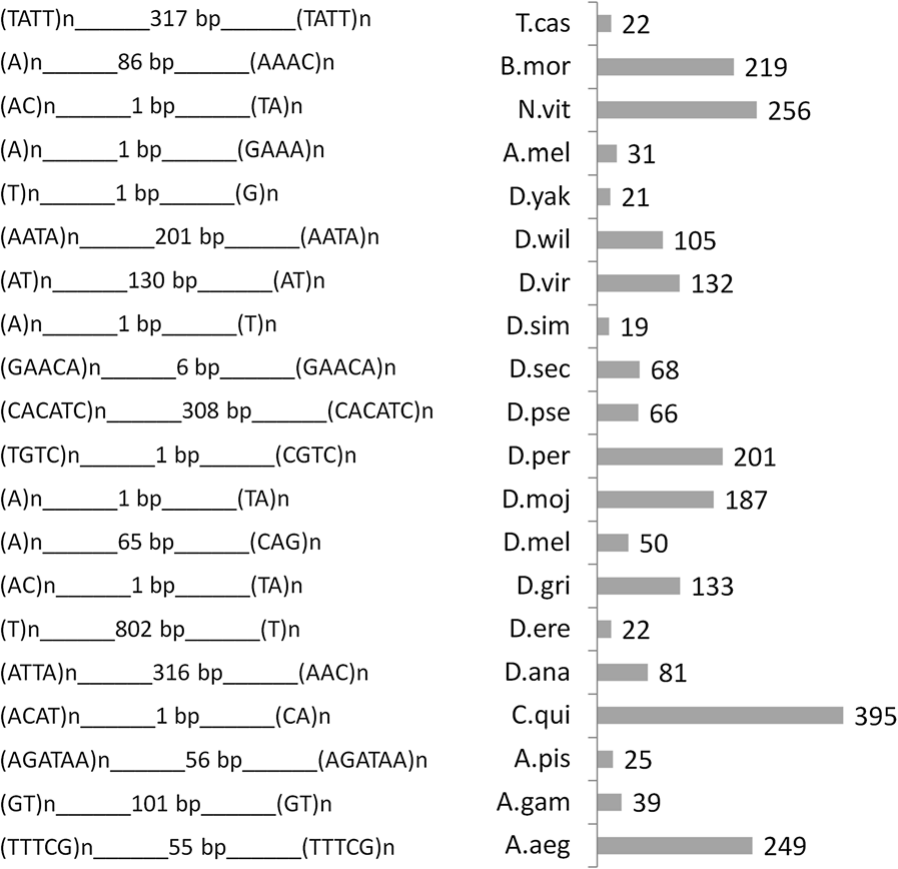
**The most repetitive microsatellite pairs in the insect species.** The rMPs are listed on the left. Corresponding to each rMP, the species names (four letters abbreviations) are shown with the number of times the pair is repeated in the genome by a bar chart.

We assumed that microsatellites with the same intervening distance are localized as pairs at different locations in the genome by random chance. Based on the number of paired microsatellites with same and/or different sequences and intervening distance, the distribution of the rMP represents a hypergeometric distribution as illustrated in Figure 2. We performed hypergeometric tests with Bonferroni correction as described in Methods to determine if microsatellite pairing is a random or non-random event. The results revealed that many of the rMPs are distributed in non-random manner (*p* < 0.05, Additional file 3) in the insect genomes. They account for 35 - 67% of the total number of rMPs identified in the genomes. The motif sequences of the most repetitive microsatellite pairs for each species are shown in Figure 3. To further validate the result, we shuffled the genomic sequences of *A. aegypti* (see Methods) and repeated the hypergeometric tests on rMP distribution in the shuffled sequences. We didn’t find a significant correlation in the distribution of rMPs in the shuffled sequences (data not shown). It was found that ~ 96% of the rMPs identified from the shuffled sequences showed random distribution pattern and lacked statistical significance after the Bonferroni correction. This suggested that the genomic distribution of microsatellite pairs is not due to a random chance but may be a characteristic feature of the genome structure of the organism.

### Inter- and intra-chromosomal distribution of rMPs

**Figure 4:**
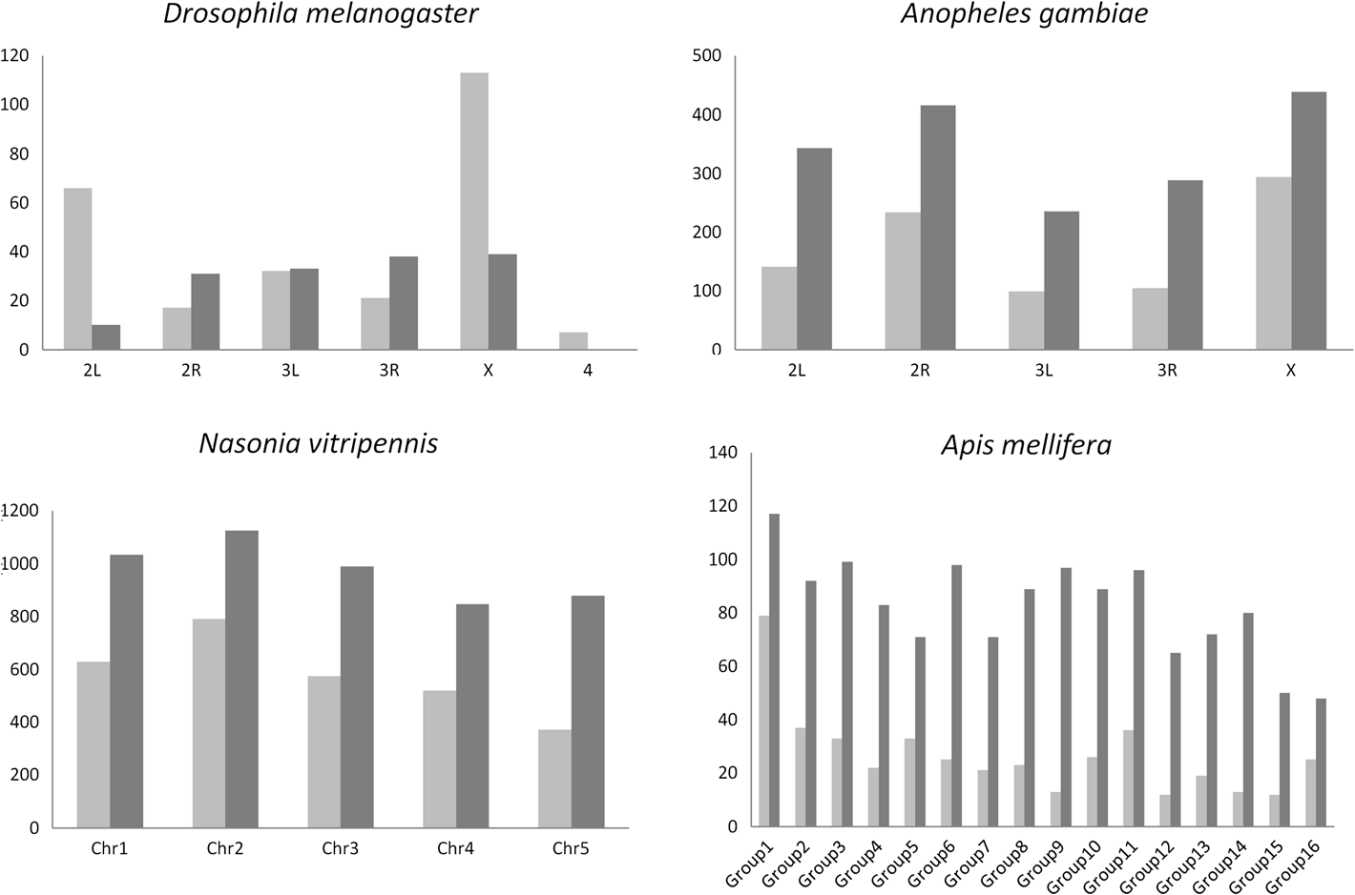
**Inter- and intra-chromosomal duplications of microsatellite pairs.** The column graphs show the number of duplications (shown on y-axis) in each chromosome annotated from genome sequences. The light grey columns represent intra-chromosomal duplication and the dark grey columns represent inter-chromosomal duplications. Only representative genomes are shown here.

**Figure 5:**
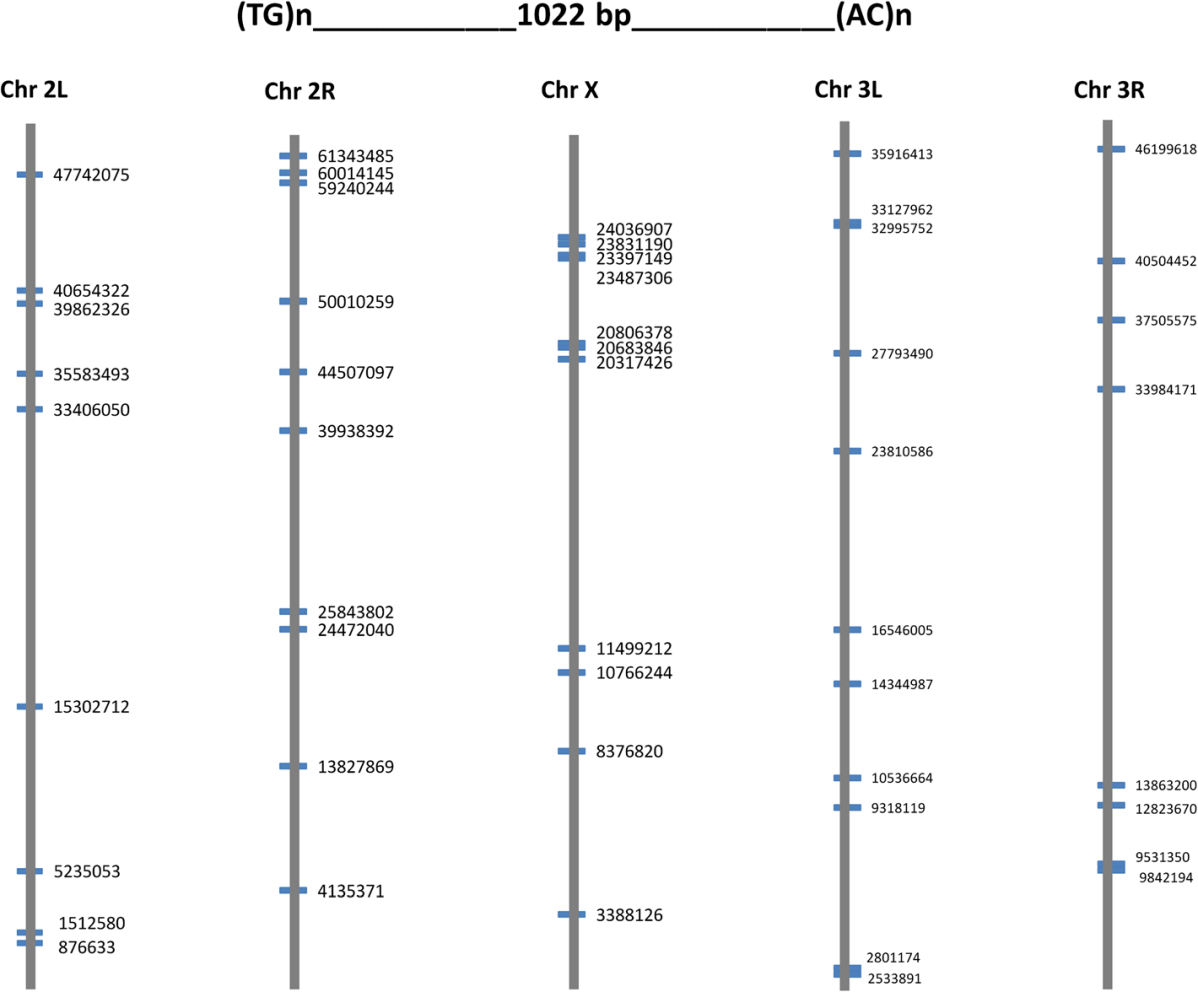
**The rMP: (TG)n-1022 bp-(CA)n of *Anopheles gambiae*.** It is repeated 40 times in the genome as shown in different chromosomes. The locations are indicated by mid-point positions of the pair in each chromosome.

The inter- and intra- chromosomal repetition of microsatellite pairs were investigated in selected species where chromosome assignments of genome sequences are available (*D. melanogaster, A. gambiae, N. vitripennis* and *A. mellifera*). It was found that the inter-chromosomal duplications are generally more frequent than intra-chromosomal duplications (Figure 4). However, in chromosome X and 2L of *D. melanogaster*, rMPs are duplicated more often within chromosomes than between chromosomes. In *D. melanogaster* and *A. gambiae*, many chromosome specific rMPs were identified (Additional file 4). These duplications are restricted to either X chromosome or autosomes but never shared by both sex chromosome and an autosome. The *A. gambiae* genome contains ~3 fold greater number of such sequences than that of the *D. melanogaster* genome. However, the frequencies of these chromosome specific rMPs are relatively lower (2–10 times) compared to frequencies of rMPs shared by both sex and autosomes. Furthermore, it was found that the shared rMPs may be selected differentially between the sex chromosome and autosomes. For example, the rMP (TG)n-1022 bp-(CA)n is repeated 40 times and shared between autosomes and the sex chromosome in *A. gambiae* (Figure 5). Sequence analysis showed contrasting patterns of evolution of these rMPs between the sex chromosome and autosomes indicating differential selection of sex-chromosome duplications compared to the autosomal duplications (Table 3).

**Table 3:** **Sequence polymorphisms among duplicated copies of mSD [(TG)n-1022 bp- (CA)n] in sex and autosomes of *A. gambiae***

**Chromosome**	**n**	**N**	**S**	**k**	**Tajima’s D**	**Significance**
X	7	644	618	336.7	1.655	Not significant
2	17	667	632	305.6	2.37	Significant
3	15	646	608	303.6	2.35	Significant

n= number of sequences, N= total number of mutations, S= no. of polymorphic sites and k= average number of nucleotide differences.

### rMP dependent aggregation of segmental duplications

**Figure 6:**
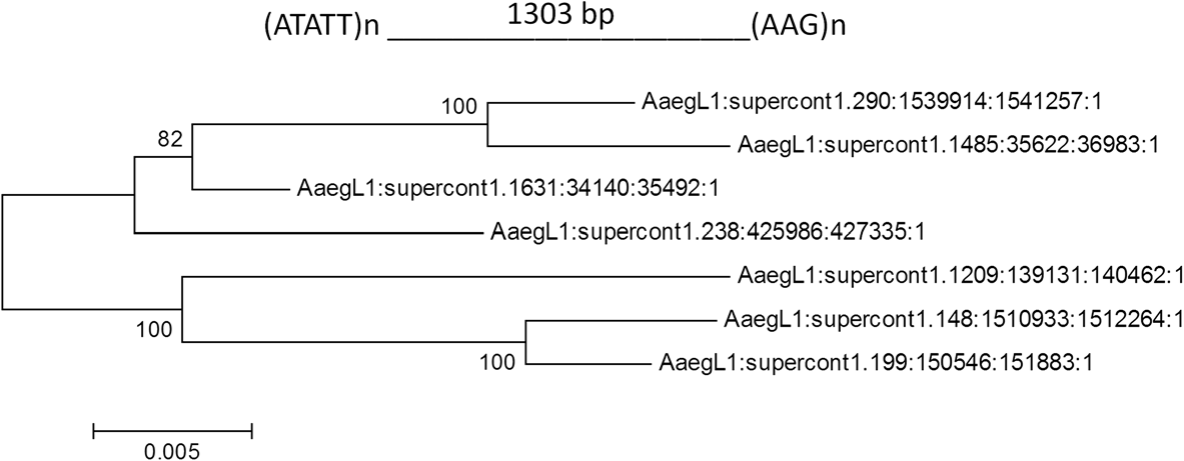
**The neighbor-joining phylogenetic tree of mSD sequences identified from the *Aedes aegypti *genome.** Each sequence is associated with the microsatellite pair (ATATT)n and (AAG)n located 1303 bp from each other. The percentage of replicate trees in which the associated taxa clustered together in the bootstrap test (1000 replicates) is shown next to the branches. The tree is drawn to scale (shown below the tree), with branch lengths in the same units as those of the evolutionary distances used to infer the phylogenetic tree. The evolutionary distances were computed using the maximum composite likelihood method and are in the units of the number of base substitutions per site.

The rMPs with intervening distances greater than 1 kb are low copy repeats in contrast to rMPs of lengths less than 1 kb (Additional file 5). We identified rMPs with intervening distance greater than 1 kb wherein sequence identity of the intervening DNA is greater than 90% [23] to identify mSDs in each species (Table 4). The Neighbor-joining trees also confirmed the sequence relatedness of these duplicated loci (Figure 6). The sequences of duplicated copies of specific mSDs in select species are shown in Additional file 6.

**Table 4:** Number of repetitive microsatellite pairs (rMPs) and the microsatellite pairs associated with segmental duplications (mSDs) in insects

**Species**	**rMP**	**mSD**	**Percentage**
A.aeg	4644	1023	22.03
A.gam	6674	891	13.35
A.mel	2861	193	6.75
A.pis	1961	40	2.04
B.mor	4920	545	11.08
C.qui	4828	573	11.87
D.ana	1731	108	6.24
D.ere	550	49	8.91
D.gri	21863	790	3.61
D.mel	2618	290	11.08
D.moj	24975	686	2.75
D.per	4579	184	4.02
D.pse	2125	97	4.56
D.sec	2062	57	2.76
D.sim	902	10	1.11
D.vir	16699	628	3.76
D.wil	11936	532	4.46
D.yak	1000	30	3
N.vit	23314	2759	11.83
T.cas	789	7	0.89

The % of rMPs classified as mSDs is shown for each species.

**Figure 7:**
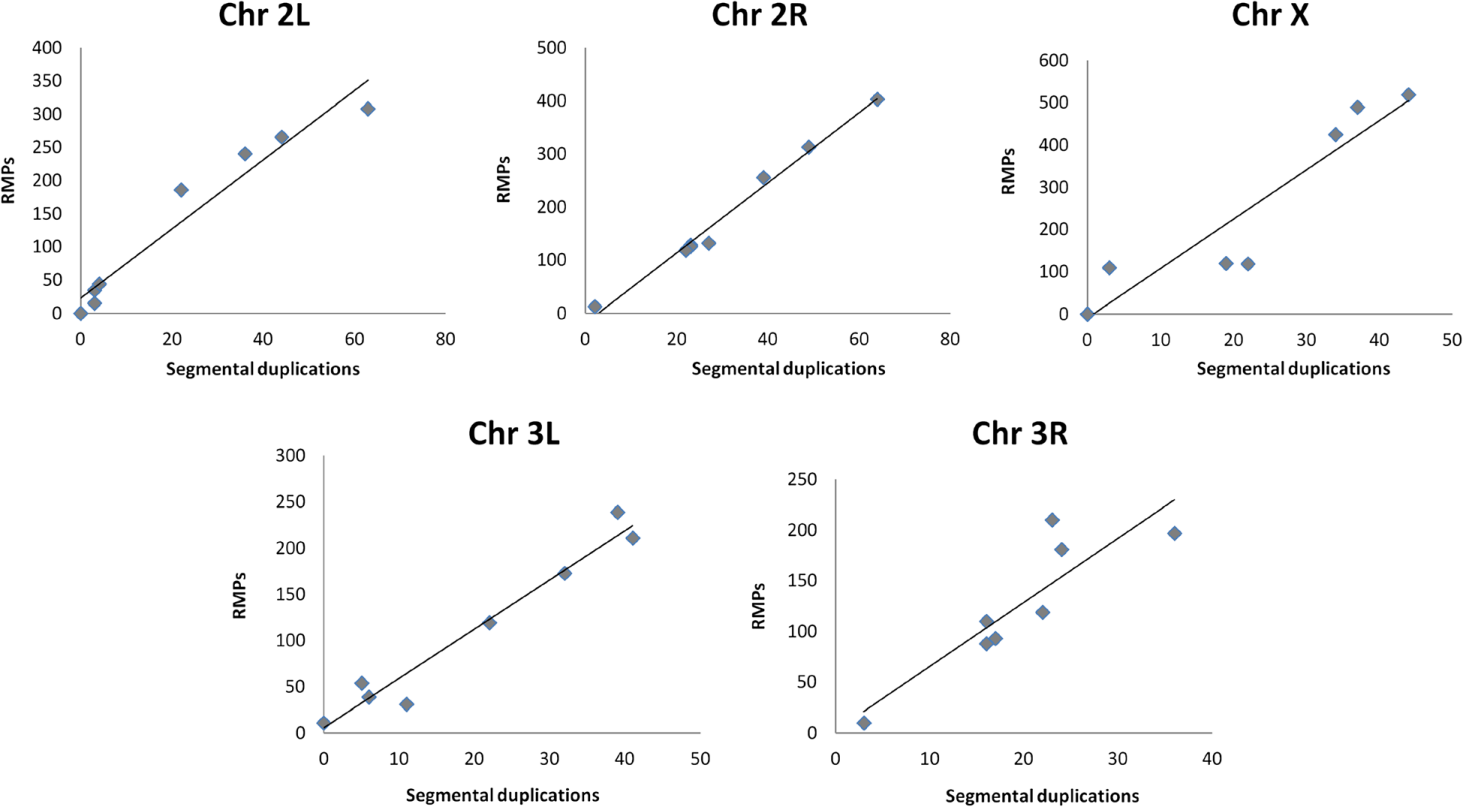
**Within-chromosome changes in frequency of microsatellites-associated segmental duplications with frequency of repetitive paired microsatellites in *Anopheles gambiae*.** The number of segmental duplications is shown on the x-axis and the number of repetitive microsatellite pairs is shown on the y-axis of the scatter plots for different windows ( < 1 Mb, 1–5 Mb, 5–10 Mb, 10–20 Mb, 20–30 Mb, 30–40 Mb, 40–50 Mb and > 50 Mb) of each chromosome.

**Figure 8:**
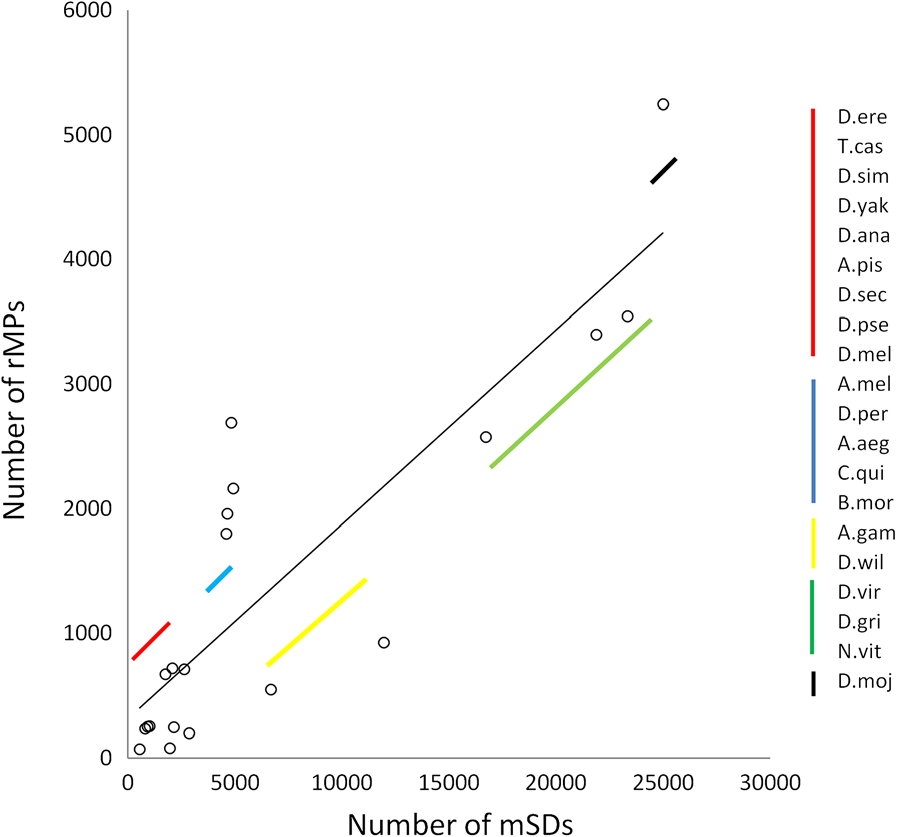
**Correlation between total number of segmental duplications and total number of repetitive microsatellite pairs among the 20 insect species.** The scatter plot shows the correlation between the number of mSDs (x-axis) and number of rMPs (y-axis) among the 20 species. However, the co-variation between mSD and rMP doesn’t correspond to the known phylogenetic relatedness of the species. Different species have been grouped based on variations in mSD and rMP values. They have been grouped by lines of different colors that matches to the species represented by data points in the graph.

We performed a sliding-window analysis of the *A. gambiae* genome to determine the frequency of rMPs and mSDs in each chromosome. The individual chromosomes were searched in different intervals to calculate the total number of mSDs as well as the number of repetitive paired microsatellites. It was found that the frequency of mSDs increases linearly (r^2^ > 78%) with the frequency of paired microsatellites in each chromosome (Figure 7). Similar results were also observed for chromosomes of other insects (Additional file 7). These results clearly suggest that regions that are already rich in rMPs tend to get richer in segmental duplications. This ‘rich-gets-richer’ based co-aggregation of mSDs along with rMPs suggest an rMP dependent mechanism of aggregation of duplicated sequences in the genome, as part of the two-step process of genesis of segmental duplications [24]. We then wanted to know whether higher segmental duplications with higher frequency of paired microsatellites is a within-genome feature or are they also correlated across genomes. By comparing the number of mSDs and the number of rMPs across the 20 species, it was found that the total number of segmental duplications increases significantly (Spearman correlation = 0.78, two tailed *p*-value = 0.00004) with increases in the total number of rMPs among the species (Figure 8) suggesting that the ‘rich-gets-richer’ mechanism may be a universal mode of rMP dependent aggregation of mSDs across genomes. However, it seems unlikely that the correlation between paired microsatellites and segmental duplications evolve in a phylogeny dependent manner among species. It is evident from Figure 8 that there is no correspondence between species phylogeny and the correlated variation between mSDs and rMPs.

### Genic *versus* non-genic association of mSDs

By mapping the mSD sequences to the annotated gene locations, we identified duplicated copies which are localized within or overlapping the coding and non-coding genes (Additional file 8). These genes represented different gene ontologies (GO) in different insects (Additional file 9), among which the ‘protein binding’ or ‘nucleus’ gene ontologies represented the top ranking predicted functions across species. The genic duplications accounted for only ~5-15% of the mSDs identified in different species indicating that the majority of duplications occur in the intergenic regions. The lower abundance of mSDs in genic regions compared to the non-genic regions may be related to differential selection pressure between genic and non-genic regions. For example, the duplications of (TG)n-1022 bp-(CA)n in the *A. gambiae* genome have genic copies (2L:47741478–47742671, 2R:4134771–4135970, 3L:10536049–10537278, 3R:12823084–12824255, 3R:13862599–13863800 and 3R:33983540–33984801) (see Figure 5). We determined the evolutionary divergence of the genic *versus* non-genic duplications (see Methods). It was found that the genic copies have lower average evolutionary divergence than that of the non-genic copies (0.32 *versus* 0.58, respectively) indicating a possibility of selection constraint on genic duplications.

In addition, we also investigated whether genes of *A. aegypti* that overlap with mSDs are also associated with the same mSD copies in the one-to-one orthologous copies in *C. quinquefasciatus* and *A. gambiae*. Our results show that these mSDs are never retained in orthologous genes (data not shown), indicating the possibility of biased selection of mSD sequences of orthologous genes. Such bias is most likely associated with purifying selection as such microsatellites in gene rich segmental duplications are known to be associated with such selection bias [22]. Furthermore, our analysis indicated that the mSDs within protein coding genes are mostly localized in the intron regions (data not shown). Hence, the lack of retention of mSDs in orthologous genes may also be due to higher rate of intron evolution than the coding sequences among the genes [32].

**Figure 9:**
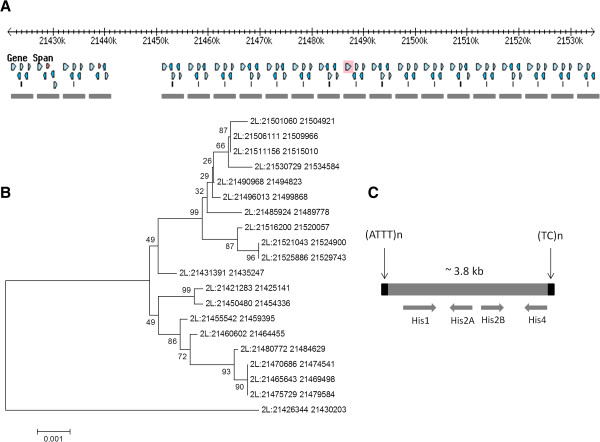
**Segmental duplications of the microsatellite pair (ATTT)n__3810 bp__(TC)n within chromosome 2L (2L:21421283–21534584) of *D. melanogaster*. A)**. The chromosome 2L region (2L:21421283–21534584) along with the gene positions and orientation of *D. melanogaster* are shown (FlyBase Genome Browser). The mSDs are indicated by grey horizontal lines below the genes. **B)**. Neighbor-joining phylogenetic tree of the twenty segmental duplications. The percentages of replicate trees in which the associated taxa clustered together in the bootstrap test (1000 replicates) are shown next to the branches. The tree is drawn to scale (shown below the tree) that shows branch lengths in the same units as those of the evolutionary distances used to infer the phylogenetic tree. **C)**. Structure of duplicated sequence. Each duplication is approximately 3.8 kb in length, contains (ATTT)n at one end and (TC)n at the other end and harbors four histone genes (His 1, His 2A, His 2B and Hist 4) localized in similar position and orientation.

We also found evidence that mSDs are associated with gene duplications in specific species. The duplication of histone genes are clustered within a ~113 kb region of chromosome 2L (2L:21421283–21534584) of *D. melanogaster* (Figure 9). In this region, twenty duplicated copies of a mSD was found wherein each copy (~3810 bp long) has (ATTT)n and (TC)n as termini sequences and harbors four histone genes (His1, His2A, His2B and His4). This pattern of duplication is often called ‘duplication shadowing’ [33] as the duplicated sequences tend to cluster in the region along with an ancestral copy. In this region of the *D. melanogaster* genome, the locus corresponding to 2L: 21426344–21430203 is the ancestral duplication as evident from phylogenetic analysis (Figure 9). As the duplications are anchored by same pair of microsatellites, it is likely that the ancestral copy might have expanded in that region by microsatellite mediated segmental duplications.

To further confirm if mSDs are associated with gene duplications, we identified the ‘nearly identical paralogous’ genes (NIPs, see Emrich *et al.*[31] for definition). We were able to find several NIPs in the *C. quinquefasciatus* and *D. melanogaster* genomes (Additional file 10) that were associated with mSDs. However, we didn’t find any NIP associated with an mSD in *A. aegypti*, *A. mellifera*, *A. gambiae* and *T. castaneum*. Thus, if microsatellite mediated SDs have a role in gene duplication in insects; it is likely that such association is species-specific.

To determine if mSDs may have association with transposable elements (TEs) [34], we analyzed the annotated TEs to identify sequence duplications that are anchored by microsatellite pairs (Additional file 11). A list of the paired microsatellites associated with different TEs in *D. melanogaster* is provided in Additional file 12. It shows that the total repertoire of mSDs associated with TEs is only a minor fraction of the total number of mSDs observed in *Drosophila*. This suggests that the mSDs are found primarily in TE-free and gene-free regions of the genome.

## Discussion

In this study, we identified microsatellites that are repeated as pairs and investigated their association with segmentally duplicated sequences in insect genomes. We adopted a conservative approach to identify the repetitive microsatellite pairs in the genome by imposing the criterion that each pair has exactly the same intervening distance. However, we observed that, in some cases, the intervening distances are not exactly same but are similar (± 1 to 20 bp) among the microsatellite pairs. For example, the microsatellite pairs (ATTT)n and (TC)n are repeated 6 times wherein the intervening distance is exactly 3,807 bp (rMP family# 55, Additional file 2) compared to the other 14 duplications of the same SSR pairs but with intervening distance ~3,810 bp (Figure 9). The variation in intervening distances between the microsatellites may have resulted due to increase or decrease of repeat length of one or both of the microsatellites, possibly by slippage events during replications [35,36]. Slippage creates a loop in one of strands that gives rise to an insertion or a deletion in the subsequent replications depending upon if the loop is formed in the replicating strand or in the template strand respectively. This leads to an increase or decrease in repeat length of microsatellites. In most of the microsatellite pairs we identified, one of the microsatellites was variable in length while the length of the other microsatellite remained unchanged. It is known that sequence composition [35], imperfection in microsatellite motifs [14] and the local mutation rate of microsatellite loci [37] have roles in modulating the repeat length of the microsatellites that may account for variable intervening distances of paired microsatellites. Furthermore, differential selection of simple sequence coding repeats [10,38] may also account for the variation in distance between microsatellite pairs.

More than two microsatellites repeated together in the genome were also identified from our analysis. For example, a cluster of microsatellites [(A)_21_..66bp..(CTG)_16_..346bp..(TA)_24_..253bp..(CA)_27_..335bp..(AGGA)_23_..711bp..(AG)_21_..1299bp..(CGGCA)_15_..225bp..(A)_21_] is repeated three times in a tandem manner within the region 2L: 9475131–9483718 of *D. melanogaster* genome. However, such repeats containing more than two microsatellites were exceptionally low in frequency in the insect genomes (data not shown). On the other hand, repeats consisting of only two microsatellites are abundant in each species which was also observed by Kofler *et al.*[14].

Segmental duplications have been characterized in few organisms, mostly in the human and *D. melanogaster* genomes [39]. They are poorly studied in other species in spite of availability of draft genome sequences for many eukaryotes. Our study is a first effort in this direction to identify segmentally duplicated sequences from genome assemblies of different insects. In this study, the segmental duplications represent only a proportion of duplications where we find microsatellites at the sequence ends of the duplications. Although a comprehensive discovery of all the segmental duplications of these insects was not the aim of the present study, our results show that the repetitions of microsatellite pairs are associated with segmental duplications in insects but with extremely variable frequency. The *A. aegypti* and *N. vitripennis* genomes have more than one thousand mSDs whereas the *T. castenium* genome has only seven mSDs (Table 4) indicating that microsatellite anchored segmental duplications may be determined by species specific evolutionary processes.

Our results further showed that genomic regions with higher numbers of repetitive microsatellite pairs accumulate a greater number of segmental duplications than regions poor in paired microsatellites (Figure 7). This is a classic ‘rich-gets-richer’ mechanism where more segmental duplications tend to occur in regions that already have more duplicated sequences [21]. Such a mode of enrichment of SDs in specific chromosomal regions has relevance to ‘duplication shadowing’ effects in genome [33,40]. For example, duplication shadowing in the human genome contributes to ~10 fold increased probability of sequence duplication in specific regions compared to their distribution in other regions [33]. We observed such a pattern of segmental duplications in chromosome 2L (2L: 21421283–21534584) of *D. melanogaster* where two (ATTT)n and (TC)n are repeated as sequence ends of each duplication (Figure 9). In this case, each SD contains four histone genes His1, His2A, His2B and His4. It was found that the entire ~3810 bp sequence representing the segmental duplication maps to a single cDNA (accession # AY119274) suggesting that the duplicated sequence containing the four genes is expressed as a common primary transcript. It is possible that duplication shadowing of gene regions may be an evolutionary strategy to modulate expression of specific genes as evident in primates [33]. Moreover, Korbel *et al.*[41] also found that segmental duplications of larger sequences enclosing specific protein coding genes often contribute to the expansion of protein-coding gene families. Although the role of microsatellites in this process is not known, it has been found that microsatellites in the flanking sequences of genes may have a regulatory role in gene expression [5]. Moreover, simple sequence repeats in the coding region can influence translational selection of genes that can modulate expression level of those genes [10]. These reports indicate that microsatellite mediated segmental duplications may have an effect on expression of the genes when they are associated with segmental duplications in the genome.

The paired microsatellites identified from our investigation may be targets of non-homologous end joining (NHEJ), which is one of the mechanisms of segmental duplication [42]. Such processes are generally mediated by microhomologies (< 25 bp homology) at the ends of target sequences similar to the termini microsatellites of mSDs found in this study. Consistently, association of microsatellites has been indicated in genomic rearrangements [43] as well as segmental duplications [44]. Furthermore, it has been shown that microsatellites are enriched at breakpoints of SDs suggesting the possible role of microsatellite repeats in the genesis of SDs [20]. Hence, our results further corroborate that microsatellites, by repetition as pairs, are likely to have a role in the genesis of SDs in insect genomes.

It is also likely that mechanisms other than involving microsatellites have roles in segmental duplications. Non-allelic homologous recombination (NAHR) during meiosis using pre-existing repeat elements (such as Alu repeats) can also lead to segmental duplications [44]. Moreover, several factors such as length, orientation, degree of sequence similarity and the distance between the duplicated copies may lead to differential degrees of genomic rearrangements of sequences in genome [45]. It is possible that the genesis of segmental duplications may also be controlled by the same mechanisms that generate copy number variations (CNV) in genomes. CNVs are caused by different rearrangement events of sequences including deletions, duplications, inversions, and translocations [46]. However, Kim *et al.* found that only a minor portion (< 10%) of CNVs is associated with segmental duplications in the human genome suggesting independent mechanisms of genesis of SDs than that of CNVs.

Our data further suggests that duplications of paired microsatellites are localized mostly in the non-genic regions. In addition to that, the paired microsatellites in the genic regions are predominantly in the intron regions (data not shown). We also found several mSDs that are associated with different transposable elements (TEs) in the *D. melanogaster* genome (Additional file 12). Therefore, the role of microsatellites in intron evolution and retrotransposition events cannot be ruled out [32,47]. Given the role of transposition events in genome structure and function [48,49], it is likely that microsatellites are instrumental in extensive sequence transposition and duplication in the genome.

## Conclusions

In this study, we have shown that microsatellites have significant association with segmental duplications in insect genomes. The repetitive paired microsatellites tend to accumulate in regions rich in segmental duplications suggesting a “rich-gets-richer” mode of aggregation of the duplicated sequences in the genome. Results further suggest that these repetitive sequences are also associated with gene duplications in specific insect genomes. The study clearly suggests that repetition of paired microsatellites contribute to extensive sequence duplications in insect genomes.

## Competing interests

Both authors declare that they have no competing interests.

## Authors’ contributions

Conceived and designed the experiments: SKB. Analyzed the data: SKB. Contributed reagents/materials/analysis tools: SKB, DWS. Wrote the paper: SKB, DWS. Both the authors read and approved the final manuscript.

## Authors’ information

SKB is a Research Assistant Professor in the Department of Biological Sciences and the Eck Institute for Global Health at the University of Notre Dame, Indiana. He has a broad interest in insect genomics and evolution with emphasis on disease transmitting vector species. DWS is a Professor of Biological Sciences and the Director of Eck Institute for Global Health at the University of Notre Dame, Indiana. His work focuses on genetic and genomic analysis of mosquito vector competence to various pathogens as well as on development and application of molecular tools to investigate population biology of mosquitoes.

## Supplementary Material

Additional file 1 Number of microsatellites (mono- though hexa-nucleotide repeats) identified from genome assemblies of different insect species. Click here for file

Additional file 2 **List of rMPs in *D. melanogaster *** **(rMPs with at least 5 copies are listed).** Click here for file

Additional file 3 **Significant enrichment of microsatellite sequences with repetitive paired microsatellites.** This is a representative list of such SSR sequences of *A. aegypti*. Click here for file

Additional file 4 **List of microsatellite pairs which are duplicated within X chromosome or an autosome.** They are not duplicated between X chromosome and an autosome. Click here for file

Additional file 5 Number of repetitive microsatellite pairs (y-axis) varies with the distance (x-axis) between the two microsatellites. Click here for file

Additional file 6 Sequence information of duplicated copies of representative mSDs in specific species. Click here for file

Additional file 7 Variation between the numbers of rMPs (y-axis) and the corresponding numbers of mSDs (x-axis) in different chromosomal regions. Click here for file

Additional file 8 List of genes associated with repetitive microsatellite pairs (rMPs) in different species. Click here for file

Additional file 9 The top five ranking gene ontology (GO) terms of genes associated with rMPs in selected species. Click here for file

Additional file 10 List of nearly identical paralogs associated with repetitive microsatellite pairs (rMP) in Culex mosquito and fruit fly genome. Click here for file

Additional file 11 **Microsatellite-anchored SDs (shown on the top) localized within the transposable elements (IDs shown below the corresponding SDs) of *D. melanogaster*.** Click here for file

Additional file 12 **Association of repetitive paired microsatellites and mSDs with transposable elements of *D. melanogaster*.** Click here for file
