# Supplementary material for: Association of microsatellite pairs with segmental duplications in insect genomes
Source: BMC Genomics. 2013 Dec 21;14:907. doi: 10.1186/1471-2164-14-907 (PMC3878106; doi:10.1186/1471-2164-14-907)
Supplement: Additional file 1 — Number of microsatellites (mono- though hexa-nucleotide repeats) identified from genome assemblies of different insect species. [file 1471-2164-14-907-S1.docx]

Number of microsatellites based on motif length (mono- though hexa-nucleotide repeats).

| Species | mono | di | tri | tetra | penta | hexa |
| --- | --- | --- | --- | --- | --- | --- |
| A.aeg | 25228 | 7605 | 38331 | 25524 | 33440 | 15824 |
| A.gam | 6898 | 47945 | 31605 | 11007 | 7453 | 2471 |
| A.mel | 9431 | 16777 | 8476 | 6382 | 4894 | 1666 |
| A.pis | 24698 | 32629 | 52316 | 15254 | 37133 | 7571 |
| B.mor | 25365 | 10455 | 17020 | 29302 | 18823 | 4305 |
| C.qui | 20272 | 17690 | 20980 | 13196 | 19151 | 13709 |
| D.ana | 7372 | 8995 | 11319 | 9892 | 9168 | 12043 |
| D.ere | 2513 | 7267 | 8246 | 4888 | 6444 | 10499 |
| D.gri | 8029 | 72281 | 42318 | 14303 | 11531 | 17583 |
| D.mel | 8557 | 12666 | 10148 | 6965 | 10222 | 6534 |
| D.moj | 23713 | 70087 | 46352 | 25723 | 14311 | 21797 |
| D.per | 4282 | 25650 | 18900 | 17691 | 21459 | 29310 |
| D.pse | 3183 | 26208 | 19066 | 13273 | 19762 | 28558 |
| D.sec | 4710 | 8011 | 8078 | 5336 | 9507 | 5213 |
| D.sim | 4179 | 8030 | 7805 | 5132 | 6124 | 5420 |
| D.vir | 13143 | 56081 | 35682 | 18304 | 8415 | 13845 |
| D.wil | 15359 | 56107 | 36881 | 28605 | 12337 | 10243 |
| D.yak | 4019 | 9920 | 9790 | 6886 | 8308 | 10378 |
| N.vit | 5902 | 84192 | 18985 | 5997 | 3213 | 1726 |
| T.cas | 579 | 870 | 6114 | 4243 | 4765 | 1328 |
