# Supplementary figures and images for: Association of microsatellite pairs with segmental duplications in insect genomes
Source: BMC Genomics. 2013 Dec 21;14:907. doi: 10.1186/1471-2164-14-907 (PMC3878106; doi:10.1186/1471-2164-14-907)

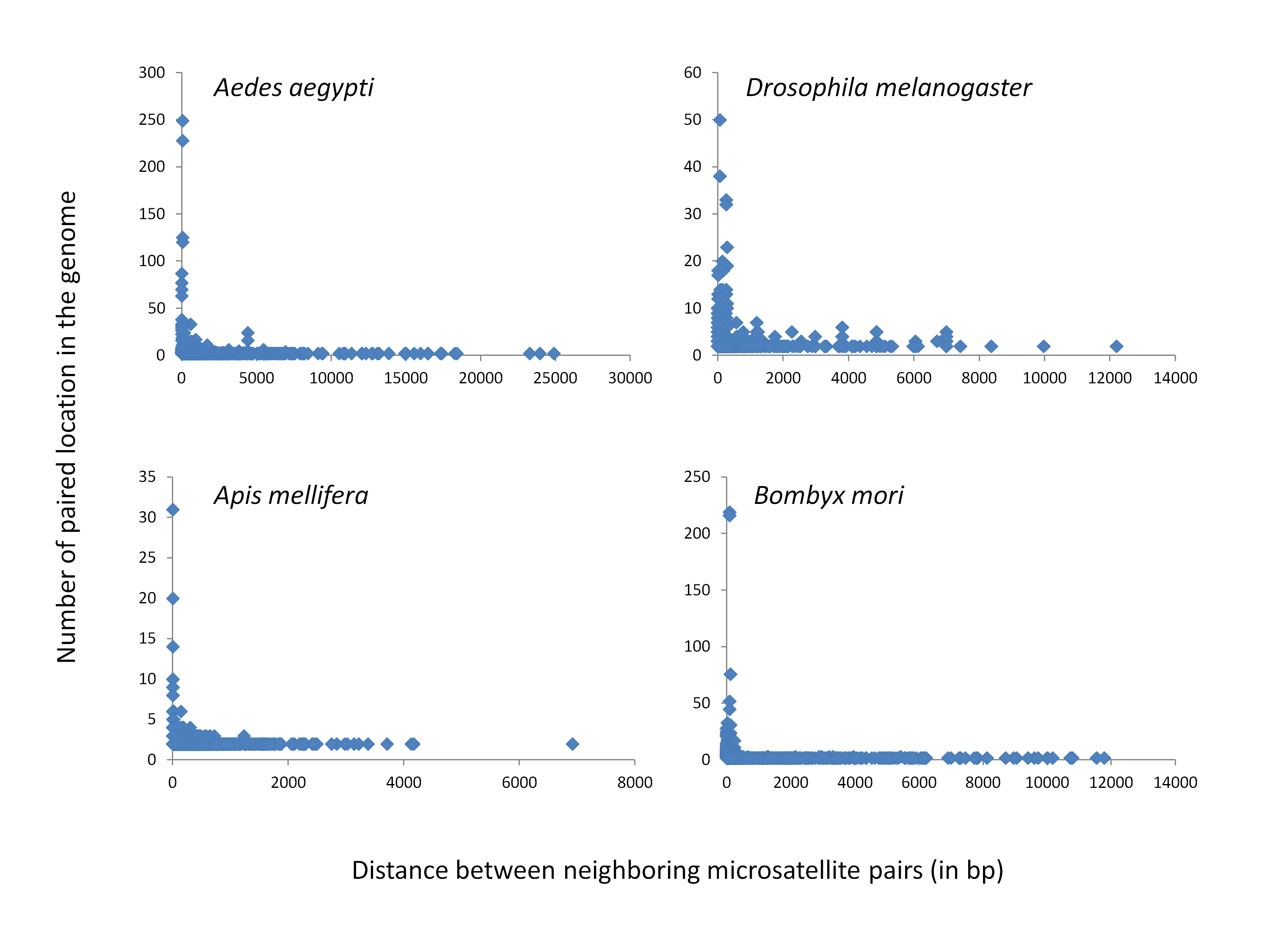

Supplement: Additional file 5 — Number of repetitive microsatellite pairs (y-axis) varies with the distance (x-axis) between the two microsatellites. [file 1471-2164-14-907-S5.tiff]

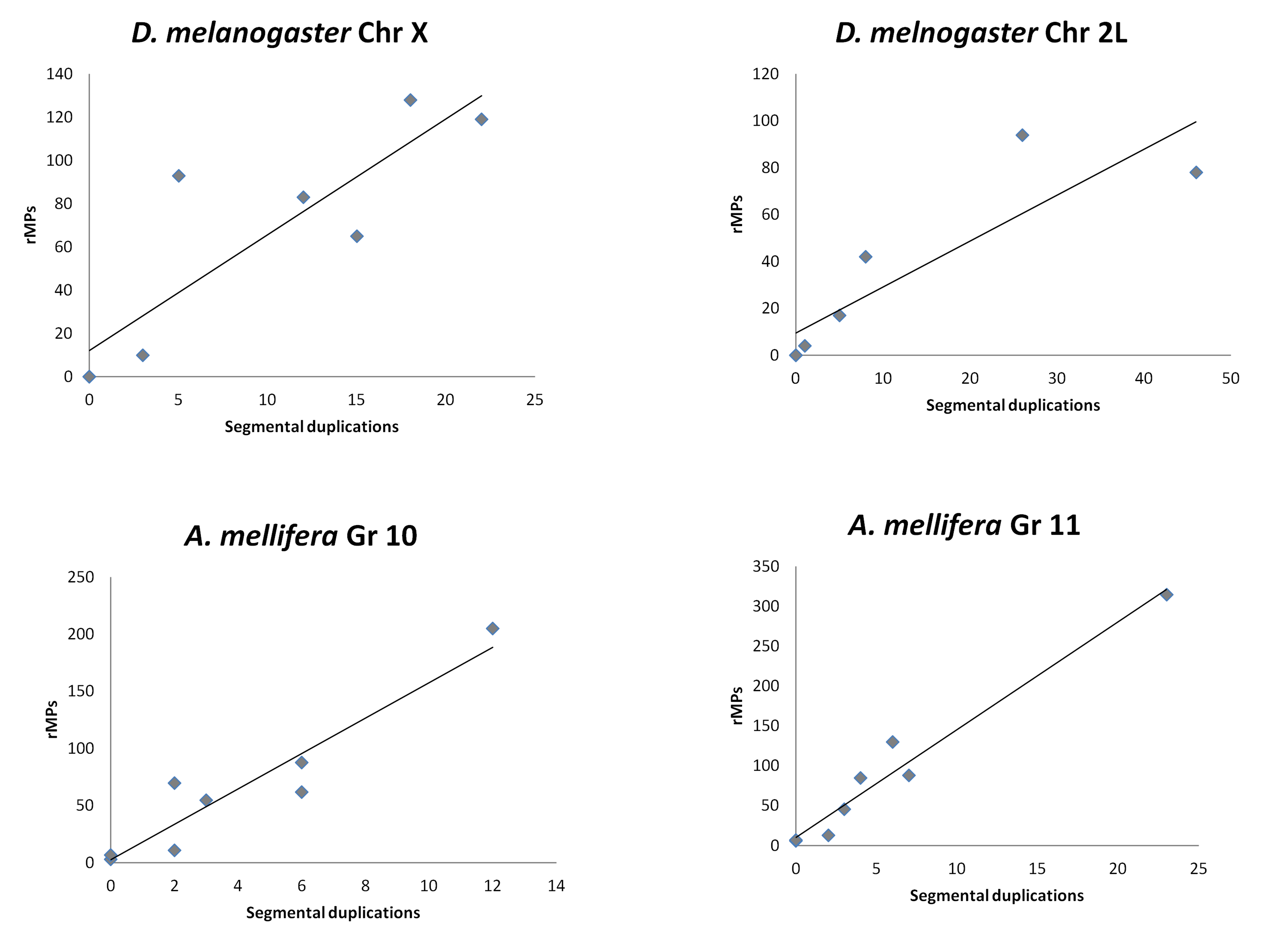

Supplement: Additional file 7 — Variation between the numbers of rMPs (y-axis) and the corresponding numbers of mSDs (x-axis) in different chromosomal regions. [file 1471-2164-14-907-S7.tiff]

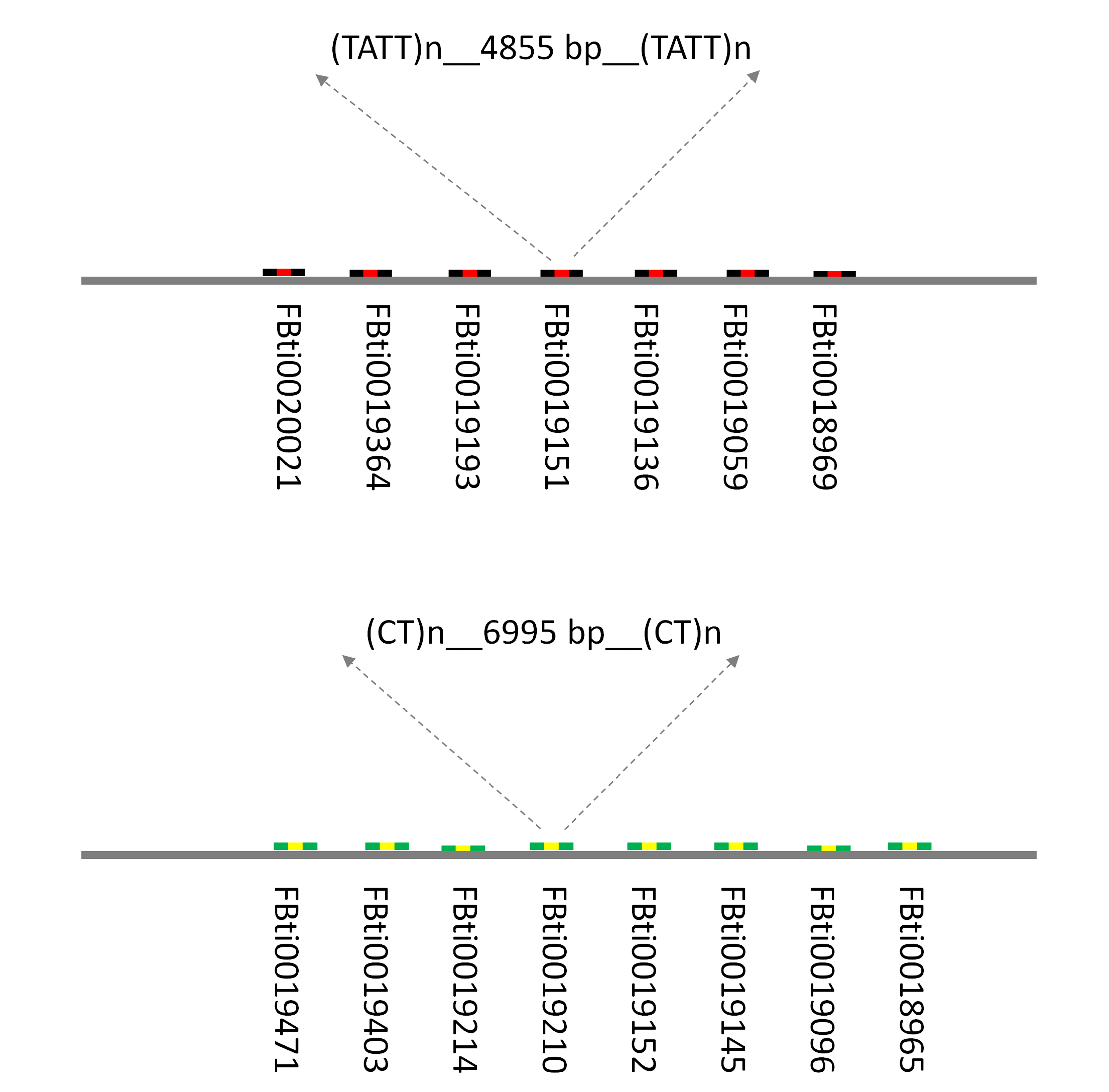

Supplement: Additional file 11 — Microsatellite-anchored SDs (shown on the top) localized within the transposable elements (IDs shown below the corresponding SDs) of D. melanogaster . [file 1471-2164-14-907-S11.tiff]
