# Supplementary material for: Association of microsatellite pairs with segmental duplications in insect genomes
Source: BMC Genomics. 2013 Dec 21;14:907. doi: 10.1186/1471-2164-14-907 (PMC3878106; doi:10.1186/1471-2164-14-907)
Supplement: Additional file 6 — Sequence information of duplicated copies of representative mSDs in specific species. [file 1471-2164-14-907-S6.docx]

**Sequence information of copies of representative mSDs.**

**A. Sequences of *A. aegypti* mSD: (ATATT)n~1303bp(AAG)n** (see Figure 6).

>AaegL1:supercont1.1209:139131:140462:1

ATATTATATTATATTCGAGTCTTAATTGATAAATTTATAAATAAACAAAATCAAAGTTTA

ATAAATAAATTAAAAATCAATGCTTTAACTACTTGGAGTACATTGTTTTCTTATTTGTTA

ACAAGTCATAAAGTCAGCCGTTTTCAAGACAATAAAATATGCTCTTTTCGGCAAATATTA

CATGAAACAAGATATCTATACCGTGTATTATGCATTCAATGTTGCAGGAGATCTCACTCA

ATCAACTCATCGATTTTTTTAATGTATCAATGCTCTTGATGGAATAGCCTGCATATTAAT

AAGGACAACAGATTCGAGTGATATCGAAATTTCCCTATCATTCAATTTTATTGAGAACTT

GGTATTTTTTTATTATTTAACTGTTTTATTATAGGTTGTGTAGGTTTATTTTATTATGAA

GAAAGACAATGAAGTTCAGCTCAAATATTAATAGAGATTAATAATTTGTATAATAATCGC

TAAAATTTTCTAAACATTCCAAATATATCAAGTGTAATCTTTTCTATATCTGGCCACTGA

TTGCCAAATGTATTTGAATTTGGATAATAATTCATTTCGAATTAATATATTTTATTGTTC

ATTTTCATTGAAATCTATTTCCTAACAAAGAATAATTAAAAAAAAACAATCCTCTAATAA

CGAAATTTCTGTTCTAACTAAAAATAAACTAGAAATATATTGTACCCTAACTCAACAATA

ACGAGCTTCATCAATCCAATCAATTTTTTTTTTTCACATTTTCTATAGTAAACTTGTTTG

TCTTGGTAACAACAGCAAAAGTAATACAATTTAGCCTATATGAAACTAGATACCAAAATT

AAGTAACAGGAACTGTGTTTATTTGAAACTGTTTTCCAAATTACTTTCGAGCGCTACTGT

ATGTCATACTGTTGGATTAGATTATCCTTACATTTTGGTCAGTAATAAATATCAGATTTT

GTATTGAACTTCTGAATTCTAATTTTTTGTTTCAACCCAATCACTGAGGAAAATTAATAA

AAAATATAAAGGGGGGGCCTTCCTTAGCCGAGTGGTTAGAGTCCGCGGCTACAAAGCAAA

GCCATGTTGAAGGAGTCTGGGTTCGATTCCCGGTCGGTCGAGGATCTTTTCGTAAAGGAA

ATTTTCTGGACTTCCCTGGGCAGAGAGTATCATCGTACCTGCCACACGATATACGAATGC

GAAAATGGCAACTTTGGCATAGAAAGCTCTCAGTGAATAACTGTGGAAGTGCTCATAAGA

ACACTAAGCTGAGAAGCTTAGTGTTCTAGGCTCTGTCCCAGTGAGGACGTCAATGCCAAG

AAGAAGAAGAAG

>AaegL1:supercont1.148:1510933:1512264:1

ATATTATATTATATTCGAGTCTTAATTGATAAATTTATTAATAAACAAAATCAAAGTTGA

ATAAATAAATTAAAAATCAATGCTTTAACTACTTGGAGTACATTGTTTTCTCATTTGTTG

ACAAGTCATGAAGTCAGCCGTTTTCAAGACAATAAAATATGCTCTTTTCGGCAAATATTA

CATGAAACAAGATATTTATACCGTGTATTATGCATTCAATGTTGCAGGAGATCTCACTTA

ATCAACTCATCCATTTGTTTTGATGTATCAATGCTCTTGATGGAATAGCCTGAATATTAA

TGAGGACAACAGATTCGAGTGATATCGAAATTTCCCTATCATTCAATTTTATTGATAACT

TGGTAATTTGATATTTTTTATTATTTAACTGTTTCATTATAGGTTGTGTAGGTTTATTTT

ATTATGAAGAAAGATAATGAAGTTCAGCTCAAATATTAATAGAGATAAATAATTTGTATA

ATAATCGCTAAAATTTTCTAAACATTCCAAATATATCAAGTGTAATCTTTTCTATATCTG

GCCACTGAATATAGATTTGAATGGATTTGAATTTGGATAATAATTCATTTCGAATTAATA

TATTTTATTGTTCATTTTCATTGAAATCTATTTCCTAACAAAGAATAATTAAAAAAAAAC

AATCCTCTAATAACGAAATTTCTGTTCTAACTAAAAATAAACTAGAAATATATTGTACCC

TAACTCAACAATAACGAGCTTCATCAATCCAATCATTTTTTTTTCACATTTTCTATAGTA

AACTTGTTTATCTTGGTAACAACAGCAAAAGTAATACAATTTAGCCTATATGAAACTAGA

TACCAAAATTATGTAACAGCAAACTGTGTTTATTTGAAACTGTTTTCCAGATAACTTTCG

AGCGCTACTGTATATCATACTGTTAGATTAGATTATCCTTACATGTTAGTCAGTAATTAA

TATCAAATTTTGTATTGAACTTCTGAATTCTAATTTTTTGTTTCAACCCAATCACTGAGG

AAAATTAATAAAAAATATAAAGGGGAGCCTTCCTTAGCCGAGTGGTTAGAGTCCGCGGCT

ACAAAGCAAAGCCATGCTGAAGGTGTCTGGGTTCAATTCCCGGTCGGTCCAGGATCTTTT

CGTAAAGGAAATTTTCTGGACTTCCCTGTGCATAGAGTATCATCGTACCTGCCACACGAT

ATACGAATGTGAAAATGGCAACTTTGGCATAAAAAGCTCTCAGTTAATAACTGTGGAAGT

GCTCATAAGAACACTAAGCTGAGAAGCAGGCTCTGTCCCAGTGAGGACGTCAATGCCAAG

AAGAAGAAGAAG

>AaegL1:supercont1.199:150546:151883:1

ATATTATATTATATTCGAGTCTTAATTGATAAATTTATTAATAAACAAAATCAAAGTTGA

ATAAATAAATTAAAAATCAATGCTTTAACTACTTGGAGTACATTGTTTTCTCATTTGTTG

ACAAGTCATAAAGTCAGCCGTTTTCAAGACAATAAAATATGCTCTTTTCGGCAAATATTA

CATGAAACAAGATATTTATACCGTGTATTATGCATTCAATGTTGCAGAAGATCTCACTTA

ATCAACTCATCGATTTGTTTTGATGTATCAATGCTCTTGATGGAATAGCCTGAATATTAA

TGAGGACAACAGATTCGAGTGATATCGAAATTTCCCTATTTTTCAATTTTATTGATAACT

TGGTAATTTGATATATTTTTATTATTTAACTGTTTCATTAAAGGTTGTGTAGGTTTATTT

TATTATGAAGAAAGATAATGAAGTTCAGCTCAAATATTAATAGAGATAAATAATTTGTAT

AATAATCGCTAAAATTTTCTAAACATTCCAAATATATCAAGTGTAATCTTTTCTATATCT

GGCCACTGAATATAGATTTGAATGGATTTGAATTTGGATAATAATTCATTTCGAATTAAT

ATATTTTATTGTTCATTTTCATTGAAATCTATTTCCTAACAAAGAATAATTAAAAAAAAC

AATCCTCTAATAACGAAATTTTCGTTCTAACTAAAAATAAACTAGAAATATATTGTACCC

TAACTCAACAATAACGAGCTTCATCAATCCAATCATTTTTTTTTCACATTTTCTATAGTA

AACTTGTTTGTCTTGGTAACAACAGCAAAAGTAATACAATTTAGCCTATATGAAACTAGA

TACCAAAATTATGTAACAGCAAACTGTGTTTATTTGAAACTGTTTTCCAAATAACTTTCG

AGCGCTACTGTATATCATACTGTTAGATTAGATTATCCTTACATTTTAGTCAGTAATTAA

TATCAAATTTTGTATTGAACTTCTGAATTCTAATTTTTTGTTTCAACCCAATCACTGAGG

AAAATTAATAAAAAATATAAAGGGGAGCCTTCCTTAGCCGAGTGGTTAGAGTCCGCGGCT

ACAAAGCAAAGCCATGCTGAAGGTGTCTGGGTTCAATTCCCGGTCGGTCCAGGATCTTTT

CGTAAAGGAAATTTTCTGGACTTCCCTGGGCATAGAGTATCATCGTACCTGCCACACGAT

ATACGAATGTGAAAATGGCAACTTTGGCATAGAAAGCTCTCAGTTAATAACTGTGGAAGT

GCTCATAAGAACACTAAGCTGAGAAGCAGGCTCTGTCCCAGTGAGGACGTCAATGCCAAG

AAGAAGAAGAAGAAGAAG

>AaegL1:supercont1.290:1539914:1541257:1

ATATTATATTATATTCGTGTCTTAATTGATAAATTTATAAATAAACAAAATCAAAGTTTA

ATAAATAAATTAAAAATCAATGCTTTAACTATTTGGAGTACATTGTTTTCTCATTTGTTA

ACAAGTCATAAAGTCAGCCGTTTTCAAGACAATAAAATATGCTCTTTTCGGCAAATATTA

CATGAAATAAGACATTTATACCGTGTATTATGCATTCAATGTTGCAGGAGATCTCACTCA

ATCAACTCATCGATTTGTTTTGATGTATCAATGCTCTTGATGGAATAGCCTGAATATTAA

TGAGGACAACAGATTCGAGTGATATCGAAATTGCCCTATCATTCAATTTTATTGATAACT

TGGTACTTTGATAATTTTTTATTATTTAACTGTTTCATTATAGGTTGTGTAGGTTTATTT

TATTATGAAGAAAGATAATGAAGTTCAGCTGAAATATTAATAGAGATAAATAATTTTTAT

AATAATCGCTAAAATTTTCTAAACATTCCAAATATATCAAGTGTAATCTTTTCTATATCT

GGCCACTGATTGCCAAATGGATTTGAATTTGGATAATAATTCATTTCGAATTAATATATT

GTATTGTTCATTTTCATTGAAATCTATTTCCTAACAAAGAATAATTTAAAAAAAAAACAA

TTCTTTAATAACGAAATTTTTGTTGTAACTAAAAATAAACTAAAAATATATTGTACCCTA

ACTCAACAATATCGAGCTTCATCAATCCAATCAATTTTTTTTTTCACATTTTCTATAGTA

AACTTGTTTGGCTTGGTGACAACAGCAAAAGTAATACAATTTAGCCTATATGAAACTAGA

TACCAAAATTAAGTAACAGGAATTGTGTTTATTTGAAACTGTTTTCCAAATTACTTTCGA

GCGCTACTGTATATCATACTGTTAGATTAGATTATCCTTACATTTTAGTCAGTAATTAAT

ATAAAATTTTGTATTGAACTTCTGAATTCTAATTTTTTGTTTCAACCCAATCACTGAGGA

AAATTAATAAAAAATGCAAAGGGGGGGCCTTCCTTAGCCGAGTGGTTAGAGTAAGCGGCT

ACAAAGCAAAGCCATGCTAAAGGTGTCTGGGTTCGATTCCCGGTCGGTCCAGGATCTTTT

CGGGTTGGAAATTTTCTCGACTTCCCTGGACATAGAGTATCATCGTACCTGCCACACGAT

ATACGAATGCGAAAATGGCAACTTTGGCATAGAAAGCTCTCAGTTAATAACTGTGAAAGT

GCTCATAAGAACACTAAGCTGAGAAGCAGGCTCTGTCCCAGAGAGGACGTCAATGCCAAG

AAGAAGAAGAAGAAGAAGAAGAAG

>AaegL1:supercont1.238:425986:427335:1

ATATTATATTATATTCGTGTCTTAATTGATAAATTTATAAATAAACAAAATCAAAGTTTA

ATAAATAAATTAAAAATCAATGCTTTAACTACTTGGAGTACATTGTTTTCTCATTTGTTA

ACAAGTCATAAAGTCAGCCGTTTTCAAGACAATAAAATATGCTCTTTTCGGCCAATATTA

CATGAAACAAGATATTTATACCGTGTATTATGCATTCAATGTTGCAGGAGATCTCACTCA

ATCAACTCATCGATTAGTTTTGATGTATTAATGCTCTTGATGGAATAGCCTGAATATTAA

TGAGGACAACAGATTCGAGTGATATCGAAATTGCCCTATCATTCAATTTTATTGATAACT

TGGTAATTTGATATTTTTTTATTATTTAACTGTTTCATTATAGGTTGTGTAGGTTTATTT

TATTATGAAGAAAGATAATGAAGTTCAGCTGAAATATTAATAAAGATAAAAAAAATTGTA

TAATAATCGCTAAAATTTTCTAAACATTCCAAATATATCAAGTGTAATCTTTTCTATATC

TGGCCACTGATTGCCAAATGGATTTGAATTTGGATAATAATTCATTTCGAATTAATATAT

TGTATTGTTCATTTTCATTGAAATCTATTTCCTAACAAAGAATAATTAAAAAAAATACAA

TCCTCTAATAACGAAAATTTTGTTCTAAATAAAAATAAAGTAAAAATATATTGTACCCTA

ACTCAACAATAACGAGCTTCATCAATCCAATCAATTTTTTTTTTAACATTTTCTATAGTA

AACTTGTTTGTCTTGGTGACAACAGCAAAAGTAATACAATTTAGCCTATATGAAACTAGA

TACCAAAATTATGTAACAGGAACTGTGTTTATTTGAAACTGTTTTCCAAATTACTTTCGA

GCGCTACTGTACATCATACTGTTAGATTAGATTATCCTAACATTTTAGTCAGTAATTAAT

ATCAAATTTTGTATTGAACTTCTGAATTCTAATTTTTTGTTTCAACCCAATCACTGAGGA

AAATTAATAAAAAATACAAAGAGGGGGCCTTCCTTAGCCAAGTGGTTAGAGTCCGCGGCT

ACAAAGCAAAGCCATGCTGAAGGTGTCTGGGTTCGATTCCCGGTCGGTCCAGGATCTTTT

CGGGTTGGAAATTTTCTCGACTTCCCTGGGCATAGAGTATCATCGTACCTGCCACACGGT

ATACGAATGCGAAAATGGCAACTTTGGCATAGAAAGCTCTCAGTTAATAACTGTGGAAGT

GCTCATAAGAACACTAAGCTGAGAAGCAGGCTCTGTCCCAGTGAGGACGTCAATGCCAAG

AAGAAGAAGAAGAAGAAGAAGAAGAAGAAG

>AaegL1:supercont1.1631:34140:35492:1

ATATTATATTATATTCGTGTCTTAATTGATAAATTAATAAATAAACAAAATCAAAGTTTA

ATAAATAAATTAAAAATCAATGCTTTAACTACTTGGAGTACATTGTTTTCTCATTTGTTA

ACAAGTCATAAAGTCAGCCGTTTTCAAGACAATAAAATATGCTCTTTTCGGCAAATATTA

CATGAAACAAGATATTTATACCGTGTATTATGCATTCAATGTTGCAGGAGATCTCACTCA

ATCAACTCATCGATTTGTTTTGATGTATCAATGCTCTTGATGGAATAGCCTGAATATTAA

TGAGGACAACAGATTCGAGTGATATCGAAATTGCCCTATCATTCAATTTTATTGATAACT

TGGTAATTTGAAATTTTTTTATTATTTAACTGTTTCATTATAGGTTGTGTAGGTTTATTT

TATTATGAAGAAAGATAATGAAGTTAAGCTGAAATATAAATAGAGATAAACAATTTGTAT

AATAATCGCTAAAATTTTCTAAACATTCCAAATATATCAAGTGTAATCTTTTCTATATCT

GGCCACTGATTGCCAAATGGATTTGAATTTGGATAATAATTCATTTCGAATTAATATATT

GTATTGTTCATTTTCATTGAAATCTATTTCCTAACAAAGAATAATTAAAAAAAAAAAACA

ATCCTTTAATAACGAAATTTTTGTTCTAACTAAAAATAAACTAAAAATATATTGTACCCT

AACTCAACAATAACGAGCTTCATCAATCCAATCAATTTTTTTTTCACATTTTCTATAGTA

AACTTGTTTGGCTTGGTGACAACAGCAAAAGTAATACAATTTAGCCTATATGAAACTAGA

TACCAAAATTATGTAACAGGAACTGTGTTTATTTGAAACTGTTTTCCAAATTACTTTCGA

GCGCTACTGTATATCATACTGTTAGATTAGATTATCCTTACATTTTAGTCAGTAATTAAT

ATCAAATTTTGTATTGAACTTCTGAATTCTAATTTTTTGTTTCAACCCAATCACTGAGGA

AAATTAATAAAAAATGCAAAGGGGGGGCCTTCCTTAGCCGAGTGGTTAGAGTCCGCGGCT

ACAAAGCAAAGCCATGCTGAAGGTGTCTGGGTTCGATTCCCGGTCGGTCCAGGATCTTTT

CGGGTTGGAAATTTTCTCGACTTCCCTGGGCATAGAGTATCATCGTACCTGCCACACGAT

ATACGAATGCGAAAATGGCAACTTTGGCATAGAAAGCTCTCAGTTAATAACTGTGGAAGT

GCTCATAAGAACACTAAGCTGAGAAGCAGGCTCTGTCCCAGTGAGGACGTCAATGCCAAG

AAGAAGAAGAAGAAGAAGAAGAAGAAGAAGAAG

>AaegL1:supercont1.1485:35622:36983:1

ATATTATATTATATTCGTGTCTTAATTGATAAATTTATAAATAAACAAAATCAAAGTTTA

ATAAATAAATTAATAATCAATGCTTTAACTACTTGGAGTACATTGTTTTCTCATTTGTTA

ACAAGTCATAAAGTCAGCCGTTTTCAAGACAATAAAATATGCTCTTTTCGGCAAATATTA

CATGAAACGAGATATTTATACCGTGTATTATGCATTCAATGTTGCAGGAGATCTCACTCA

ATCAACTCATCGATTTGTTTTGATGTATCAATGCTCTTGATGGAATAGCCTGAATATTAA

TGAGGACAACAGATTCGAGTGATGTCGAAATTGCCCTATCATTCAATTTTATTGATAACT

TGGTAATTTGATAATTTTTTATTATTTAACTGTTTCATTATAGGTTGTGTAGGTTTATTT

TATTATGAAGAAAGATAATGAAGTTCAGCTGAAATATTAATAGAGATAAATAATTTTTAT

AATAATCGCTAAAATTTTCTAAACATTCCAAATATATCAAGTGTAATCTTTTCTATATCT

GGCCACTGATTGTCAAATGGATTTAAATTTGGATAATAATTCATTTCGAATTAATATATT

GTATTGTTCATTTTCATTGAAATCTATTTCCTAACAAAGAATAATTTAAAAAAAAAGCAA

TTCTTCAGTAACGAAATTTTTGTTGTAACTAAAAATAAACTAAAAATATATTGTACCCTA

ACTCAACAATATCGAGCTTCATCAATCCAATCAATTTTTTTTTTCACATTTTCTATAGTA

AACTTGTTTGGCTTGGCGACAACAGCAAAAGTAATACAATTTAGCCTATATGAAACTAGA

TACCAAAATTAAGTAACAGGAATTGTGTTTATTTGAAACTGTTTTCCAAATTACTTTCGA

GCGCTACTGTATATCATACTGTTAGATTAGATTATCCTTACATTTTAGTCAGTAATTAAT

ATAAAATTTTGTATTGAACTTCTGAATTCTAATTTTTTGTTTCAACCCAATCACTGAGGA

AAATTAATAAAAAATGCAAAGGGGGGGCCTTCCTTAGCCGAGTGGTTAGAGTAAGCGGCT

ACAAAGCAAAGCCATGCTAAAGGTGTCTGGGTTCGATTCCCGGTCGGTCCAGGATCTTTT

CGGGTTGGAAATTTTCTCGACTTCCCTGGACATAGAGTATCATCGTACCTGCCACACGAT

GTACGAATGCGAAAATGGCAACTTTGGCATAGAAAGCTCTCAGTTAATAACTGTGGGAGT

GCTCATAAGAACACTAAGCTGAGAAGCAGGCTCTGTCCCAGTGAGGACGTCAATGCCAAG

AAGAAGAAGAAGAAGAAGAAGAAGAAGAAGAAGAAGAAGAAG

**B. An example of mSD copies in *A. gambiae* genome: (TG)n~1022bp(CA)n** (See Figure 5)

>AgamP3:2L:1511976:1513183:1

TGTGTTTGTGTTTGTGTGTGTATGTATGTGTGTGTATGTGTGTGTGTATGTGTGTGTGCA

TGTGTGTGTGTGTGTGGATGTATGTTTGAATGTGTATTGATGTGTTTGTTTGTTTCACAA

GTAGGTCGTTTCGGATAGATTTGTAAGTAGTTTTTTTGTGTTTTGGGTTAGGTTTTTGGT

GTGATAAGCAGGACCACCGCCCTCGCGATCAGTGTGTTTTCGATATATTAACAATGTTAC

GGTCTTCTTTCGCCGTGGTCTTCTGAGGACGTCCGGTTGACTTGCGCGTTTCGGTTGTCC

AAGCGCGTTTCAGTTGTCTAAGCACGTTTCGGTTGTCCGAAGCGCGTTTGCCACAAAAGT

GCGCGATCGTTCCATTACGAACTCGATCCTTTTGCGCTTAATGCCAGCAGCAGCCATTCG

CTTTTACATATGGCGCTGATAATCGGTACAACGTTTACCTCGACCCATTGTTACTAACAT

TGCTTGCTTGGACCGTCAAGAACGCGCTGGTTAAAAACTTGGACACGGCTGATCCCGTGC

TCTGCCTGCGTTCTACTTCTATACGCGATGAATTACATCGTTGTCGAGCAGCGAAAACAT

CGCATGGATAAAAACTATCATTGAGTACGCAAGTAAGCGTCTTCCCTTCAAAATCGAGAG

CAGAATACTGCCGCGCTCATGAAACAAAACGCCCATTGAAAAGAACAACTGCGCGCCAGC

TCAATGCACGCACAAAGTTTACCATTCGCACACAACGTGCAACGCAGAGATGCACGAAGG

CCTTTCAATGGCGTGCACTGGAGAAACACCTGTGAGGGAATGCCGAGTTCATTGCTATTG

TGCGTGTGTCCATTTCGCACCGGTGTCGCATTCACATTCATGAAACAGCACACCTGCGTT

TTCGTCCGAGGAACAAGCGCTGCTGTCGATGCAAACTTTAGTTTTTATCCAAGTGTAAAC

GCACAATGTTTCACATGATAGCACACATAATAAGAATAATTTCAACGCAATATGACATCA

TGGCAAGCTATGTTTTTCAGACACAAACCCGCGCACTTGCAAATACACATTAAAAAGCAC

ACTCACTTTCTACTACTACACACACACACACATGCACACACACACACACATACACACACA

CACACACACACACACACACACTCACACACATACACACACACATACACACATACACACACA

CAAACACA

>AgamP3:2L:15302114:15303309:1

TGTGTGTGTGTGTATGTGTGTGTATGTGTGTGTATGTGTGTTTGTGTGTGTGTGTGTATA

TGTGTGTGTGCATGTGTGTGTGTGTGTGGATGTATGTTTGAATGTGTATTGATGTGTGTG

TTTGTTTCACAAGTAGGTCGTTTCGAATAGATTTGTAAGTAGTTTTTTTGTGTTTTGGGT

TAGGTTTTTGGTGTGATAAGCAGGACCACCGCCCTCGCGATCAGTGTGTTTTCGATGTAT

TAACAATGTTACGGTCTTCTTTCGCCGTGGTCTTCTGAGGACGTCCGGTTGACTTGCGCG

TTTCGGTTGTCCAAACGCGTTTCAGTTGTCTAAGCACGTTTCGGTTGTCCGAAGCGCGTT

TGCCACAAAAGTGCGCGATCGTTCCATTACGAACTCGATCCTTTTGCGCTTAATGCCAGC

AGCAGCCATTCGCTTTTACATATGGCGCTGATGATCGGTACAACGTTTACCTCGACCCAT

TGTTACTAACATTGCTTGCTTGGACCGTCAAGAACGCACTGGTTAAAAACTTGGACACGG

CTGATCCCGTGCTCTGCCTGCGTTCTACTTCTATACGCGATGAATTACATCGTTTTGGAG

CAGCAAAAACATCGCATGGATAAAAACTATCAACGAGTACGCGAGTAAGCGTCTTCCCTT

CAAAATCGAGAAAAGAATACTGCCGCGCTCATGAAACAAAACGCCCATTGAAAAGAATAA

CTGCGCGCCAGCTCAATGCACGCACAAAGTTTACCATTCGCACACAACGTGCAACGCAGA

GATGCACGAAGGCCTTTCAATGGCGTGCACTGGAGAAACACCTGTGAGGGAATGCCGAGT

TCATTGCTATTGTGCGCGTGTCCATTTCGCACCGGTGTCGCATTCACATTCATGAAACAG

CACACCTGCGTTTTCGTCCGAGGAACAAGCGCTGCTGTCGATGCAAACTTTAGTTTTTAT

CCAAGTGTAAACGCACAATGTTTCACATGATAACACACATAATAAGAATAATTTCAACGC

AATATGACATCATGGCAAGCTATGTTTTTCAGACACAAACCCGCGCACTTGCTAATACAC

ATTAAAAAGCACACTCTCTTTCTACTACTACACACACACACATGCACACACACACACATA

CACACACACACACACATACACACACACACATACACACATACACACACACAAACACA

>AgamP3:2L:33405452:33406647:1

TGTGTTTGTGTTTGTGTGTGTGTGTATGTGTGTGTATGTGTGTGTGTGTATGTGTGTGTG

CATGTGTGTGTGTGTGTGTGGATGTATGTTTGAATGTGTATTGATGTGTGTGTTTGTTTC

ACAAGTAGGTCGTTTCGGATAGATTTGTAAGTAGCTTTTTTGTGTTTTGGGTTAGGTTTT

TGGTGTGATAAGCAGGACCACCGCCCTCGCGATCAGTGTGTTTTCGATATATTAACAATG

TTACGGTCTTCTTTCGCCGTGGTCTTCTGAGGACGTCCGGTTGACTTGCGCGTTTCGGTT

GTCCAAGCGCGTTTCAGTTGTCTAAGCACGTTTCGGTTGTCCGAAGCGCGTTTGCCACAA

AAGTGCGCGATCGTTCCATTACGAACTCGATCCTTTTGCGCTTAATGCCAGCAGCAGCCA

TTCGCTTTTACATATGGCGCTGATAATCGGTACAACGTTTACCTCGACCCATTGTTACTA

ACATTGCTTGCTTGGACCGTCAAGAACGCGCTGGTTAAAAACTTGGACACGGCTGATCCC

GTGCTCTGCCTGCGTTCTACTTCTATACGCGATGAATTACATCGTTGTCGAGCAGCGAAA

ACATCGCATGGATAAAAACTATCAACGAGTACGCGAGTAAGCGTCTTCCCTTCAAAATCG

AGAACAGAATACTGCCGCGCTCATGAAACAAAACGCCCATTGAAAAGAACAACTGCGCGC

CAGCTCAATGCACGCACAAAGTTTACCATTCGCACACAACGTGCAACGCAGAGATGCACG

AAGGCCTTTCAATGGCGTGCACTGGAGAAACACCTGTGAGAGAATGCCGAGTTCATTGCT

ATTGTGCGTGTGTCCATTTCGCACCGGTGTCGCATTCACATTCATGAAACAGCACACCTG

CGTTTTCGTCCGAGGAACAAGGGCTGCTGTCGATGCAAACTTTAGTTTTTATCCAAGTGT

AAACGCACAATGTTTCACATGATATCACACATAATAAGAATAATTTCAACGCAATATGAC

ATCATGGCAAGCTATGTTTTTCAGACACAAACCCGCGCACTTGCAAATACATATTAAAAA

GCACACTCACTTTCTACTACTACACACACACACATGCACACACACACACACACAAACACA

CACACACACACTCACACACACACATACACACACACATACACACATACACACACACA

>AgamP3:2L:35582898:35584087:1

TGTGTGTGTGTATGTGTGTATTTGTGTGTGTATGTGTGTGTGTGAGTGTGTGTGTGTGTG

TGTTTGTGTGTTTGTGTGTGTGTGTGTGTGTGTGTAGTAGTAGAAAGTGAATGTGCTTTT

TAATGTGTATTTGCAAGTGCGCGGGTTTGTGTCTGAAAAACATAGCTTGCCATGATGTCA

TATTGCGTTGAAATTATTCTTATTATGTGTGTTATCATGTGAAACATTGTGCGTTTACAC

TTGGATAAAAACTAAAGTTTGCATCGACAGCAGCCCTTGTTCCTCGGACGAAAACGCAGG

TGTGCTGTTTCATGAATGTGAATGCGACACCGGTGCGAAATGGACACACGCACAATAGCA

ATGAACTCGGCATTCTCTCACAGGTGTTTCTCCAGTGCACACCATTGAAAGGCCTTCGTG

CATCTCTGCGTTGCACGTTGTGTGCGAATGGTAAACTTTGTGCGTGCATTGAGCTGGCGC

GCAGTTGTTCTTTTCAATGGGCGTTTTGTTTCATGAGCGCGGCAGTATGCTTTTCTCGAT

TTTGAAGGGAAGACGCTTACTCGCGTACTCGTTGATAGTTTTTATCCATGCGATGTTTTC

GCTGCTCGACAACGATGTAATTCATCGCGTATAGAAGTAGAACGCAGGCAGAGCACGGGA

TCAGCCGTGTCCAAGTTTTTAACCAGCGCGTTCTTGACGGTCCAAGCAAGCAATGTTAGT

AACAATGGGTCGAGGTAAACGTTGTACCGATTATCAGCGCCATATGTAAAAGCGAATGGC

TGCTGCTGGCATTAAGCGCAAAAGGATCGAGTTCGTAATGGAACGATCGCGCACTTTTGT

GGCAAACGCGCTTCGGACAACCGAAACGTGCTTAGACAACTGAAACGCGCTTGGACAACC

GAAACGCGCAAGTCAACCGGATGTCCTCAGAAGACCACGGCGAAAGAAGACCGTAACATT

GTTAATATATCGAAAACACACTGATCGCGAGGGCGGTGGTCCTGCTTATCACACCAAAAA

CCTAACCCAAAACACAAAAAAGCTACTTACAAATCTATACGAAACGACCTACTTGTGAAA

CAAACACACACATCACTACACATTCAAACATACATCCACACACACACACACACACATGCA

CACACACATACACACACACATACACACACATACACACACACACAAACACA

>AgamP3:2L:39861715:39862936:1

TGTGTGTGTGTATGTGTGTATTTGTGTGTGTGTGTATGTGTGTATTTGTGTGTGTATGTG

TGTGTGAGTGTGTGTGTGTATGTGTGTGTGTGCATGTGTGTGTGTGTGTAGTAGTAGAAA

GAGAGTGTGCTTTTTAATGTGTATTAGCAAGTGCGCGGGTTTGTGTCTGAAAAACATAGC

TTGCCATGATGTCATATTGCGTTGAAATTATTCTTATTATGTGTGTTATCATGTGAAACA

TTGTGCGTTTACACTTGGATAAAAACTAAAGTTTGCATCGACAGCAGCCCTTGTTCCTCG

GACGAAAACGCAGGTGTGCTGTTTCATGAATGTGAATGCGACACCGGTGCAAAATGGACA

CGCGCACAATATCAATGAACTCGGCATTCTCTCACAGGTGTTTCTCCAGTGCACGCCATT

GAAAGGCCTTCGTGCATCTCTGCGTTGCACGTTGTGTGCGAATGGTAAACTTTGTGCGTG

CATTGAGCTGGCGCGCAGTTGTTCTTTTCAATGGGCGTTTTGTTTCATGAGCGCGGCAGT

ATTCTGTTCTCGATTTTGAAGGGAAGACGCTTACTCGCGTACTCGTTGATAGTTTTTATC

CATGCGATGTTTTCGCTGCTCGACAACGATGTAATTCATCGCGTATAGAAGTAGAACGCA

GGCAGAGCACGGGATCAGCCGTGTCCAAGTTTTTAACCAGCGCGTTCTTGACGGTCCAAG

CAAGCAATGTTAGTAACAATGGGTCGAGGTAAACGTTGTACCGATTATCAGCGCCATATG

TAAAAGCGAATGGCTGCTGCTGGCATTAAGCGCAAAAGGATCGAGTTCGTAATGGAACGA

TCGCGCACTTTTGTGGCAAACGCGCTTCGGACAACCGAAACGTGCTTAGACAACTGAAAC

GCGCTTGGACAACCGAAACGCGCAAGTCAACCGGACGTCCTCAGAAGACCACGGCGAAAG

AAGACCGTAACATTGTTAATATATCGAAAACACACTGATCGCGAGGGCGGTGGTCCTGCT

TATCACACCAAAAACCTAACCCAAAACACAAAAAAGCTACTTACAAATCTATCCGAAACG

ACCTACTTGTGAAACAAACACACACATCAATACACATTCAAACATACATCCACACACACA

CACACATGCACACACACATACACACACACACATACACACACACACATACACACACATACA

CACACACACAAACACAAACACA

>AgamP3:2L:40653737:40654906:1

TGTGTGTGTGTATGTGTGTGTATGTGTGTGTGTGTATGTGTGTGTGCATGTGTGTGTGTG

TGTGGATGTATGTTTGAATGTGTATTGATGTGTGTGTTTGTTTCACAAGTAGGTCCTTTC

GGATAGATTTGTAAGTAGCTTTTTTGTGTTTTGGGTTAGGTTTTTGGTGTGATAAGCAGG

ACCACCGCCCTCGCGATCAGTGTGTTTTCGATATATTAACAATGTTACGGTCTTCTTTCG

CCGTGGTCTTCTGAGGACGTCCGGTTGACTTGCGCGTTTCGGTTGTCCAAGAGCGTTTCA

GTTATCTATACACGTTTCGGTTGTCCGAAGCGCGTTTGCCACAAAAGTGCGCGATCGTTC

CATTACGAACTCGATCCTTTTGCGCTTAATGCCAGCAGCAGCCATTCGCTTTTACATATG

GCGCTGATGATCGGTACAACGTTTACCTCGACCCATTGTTACTAACATTGCTTGCTTGGA

CCGTCAAGAACGCGCTGGTTAAAAACTTGGACACGGCTGATCCCGTGCTCTGCCTGCGTT

CTACTTCTATACGCGATGAATTACATCGTTGTCGAGCAGCGAAAACATCGCATGGATAAA

AACTATCAACGAGTACGCGAGTAAGCGTCTTCCCTTCAAAATCGAGAACAGAATACTGCC

GCGCTCATGAAACAAAACGCCCATTGAAAAGAATAACTGCGCGCCAGCTCAATGCACGCA

CAAAGTTTACCATTCGCACACAACGTGCAACGCAGAGATGCACGAAGGCCTTTCAATGGC

GTGCACTGGAGAAACACCTGTGACGGAATGCCGAGTTCATTGCTATTGTGCGCGTGTCCA

TTTCGCACCGGTGTCGCATTCACATTCATGAAACAGCACACCTGCGTTTTCGTCCGAGGA

ACAAGCGCTGCTGTCGATGCAAACTTTAGTTTTTATCCAAGTGTAAACGAACAATGTTTC

ACATGATAACACACATAATAAGAATAATTTCAACGCAATATGACATCATGGCAAGCTATG

TTTTTCAGACACAAACCCGCGCACTTGCAAATACACATTAAAAAGCACACTCACTTTCTA

CTACTACACACACACACACATGCACACACACACACAAACACACACACACTCACACACATA

CACACACACATACACACATACACACACACA

>AgamP3:2L:42735518:42736707:1

TGTGTGTGTGTTTGTGTGTATGTGTGTGTGTATGTGTGTGTGAGTGTGTGTGTGTATGTG

TGTGTGTGTGTGCATGTGTGTGTGTGTGTAGTAGTAGAAAGAGAGTGTGCTTTTTAATGT

GTATTAGCAAGTGCGCGGGTTTGTGTCTGAAAAACATAGCTTGCCATGATGTCATATTGC

GTTGAAATTATTCTTATTATGTGTGTTATCATGTGAAACATTGTGCGTTTACACTTGGAT

AAAAACTAAAGTTTGCATCGACAGCAGCGCTTGTTCCTCGGACGAAAACGCAGGTGTGCT

GTTTCATGAATGTGAATGCGACACCGGTGCGAAATGGACACGCGCACAATAGCAATGAAC

TCGGCATTCCCTCACAGGTGTTTCTCCAGTGCACGCCATTGAAAGGCCTTCGTGCATCTC

TGCGTTGCACGTTGTGTGCGAATGGTAAACTTTGTGCGTGCATTGAGCTGGCGCGCAGTT

ATTCTTTTCAATGGGCGTTTTGTTTCATGAGCGCGGCAGTATTCTTTTCTCGATTTTGAA

GGGAAGACGCTTACTCGCGTACTCGTTGATAGTTTTTATCCATGCGATGTTTTCGCTGCT

CCAAAACGATGTAATTCATCGCGTATAGAAGTAGAACGCAGGCAGAGCACGGGATCAGCC

GTGTCCAAGTTTTTAACCAGCGCGTTCTTGACGGTCCAAGCAAGCAATGTTAGTAACAAT

GGGTCGAGGTAAACGTTGTACCGATCATCAGCGCCATATGTAAAAGCGAATGGCAGCTGC

TGGCATTAAGCGCAAAAGGATCGAGTTCGTAATGGAACGATCGCGCACTTTTGTGGCAAA

CGCGCTTCGGACAACCGAAACGTGCTTAGACAACTGAAACGCGCTTGGACAACCGAAACG

CGCAAGTCAACCGGACGTCCTCAGAAGACCACGGCGAAAGAAGACCGTAACATTGTTAAT

ATATCGAAAACACACTGATCGCGAGGGCGGTGGTCCTGCTTATCACACCAAAAACCTAAC

TCAAAACACAAAAAAACTACTTACAAATCTATCCGAAACGACCTACTTGTGAAACAAACA

CACACATCAATACACATTCAAACATACATCCACACACACATACACACACACACACACACA

CACACACACACAAACACACATACACACACATACACACACACACAAACACA

>AgamP3:2L:47741478:47742671:1_genic

TGTGTGTGTGTATGTGTGTATTTGTGTGTGTGTATGTGTGTGTGTGAGTGTGTGTGTGTG

TGTGTTTGTGTGTTTGTGTGTGTGTGTGTGTGTGTGTAGTAGTAGAAAGTGAATGTGCTT

TTTAATGTGTATTTGCAAGTGCGCGGGTTTGTGTCTGAAAAACATAGCTTGCCATGATGT

CATATTGCGTTGAAATTATTCTTATTATGTGTGTTATCATGTGAAACATTGTGCGTTTAC

ACTTGGATAAAAACTAAAGTTTGCATCGACAGCAGCCCTTGTTCCTCGGACGAAAACGCA

GGTGTGCTGTTTCATGAATGTGAATGCGACACCGGTGCGAAATGGACACACGCACAATAG

CAATGAACTCGGCATTCTCTCACAGGTGTTTTTCCAGTGCACACCATTGAAAGGCCTTCG

TGCATCTCTGCGTTGCACGTTGTGTGCGAATGGTAAACTTTGTGCGTGCATTGAGCTGGC

GCGCAGTTGTTCTTTTCAATGGGCGTTTTGTTTCATGAGCGCGGCAGTATTCTTTTCTCG

ATTTTGAAGGGAAGACGCTTACTCGCGTACTCGTTGATAGTTTTTATCCATGCGATGTTT

TCGCTGCTCGACAACGATGTAATTCATCGCGTATAGAAGTAGAACGCAGGCAGAGCACGG

GATCAGCCGTGTCCAAGTTTTTAACCAGCGCGTTCTTGACGGTCCAAGCAAGCAATGTTA

GTAACAATGGGTCGAGGTAAACGTTGTACCGATTATCAGCGCCATATGTAAAAGCGAATG

GCTGCTGCTGGCATTAAGCGCAAAAGGATCGAGTTCGTAATGGAACGATCGCGCACTTTT

GTGGCAAACGCGCTTCGGACAACCGAAACGTGCTTAGACAACTGAAACGCGCTTGGACAA

CCGAAACGCGCAAGTCAACCGGATGTCCTCAGAAGACCACGGCGAAAGAAGACCGTAACA

TTGTTAATACATCAAAAACACACTGATCGCGAGGGCGGTGGTCCTGCTTATCACACCAAA

AACCTAACCCAAAACACAAAAAAGCTACTTACAAATCTATCCGAAACGACCTACTTGTGA

AACAAACACACACATCAATACACATTCAAACATACATCCACACACACACACACACACACA

TGCACACACACATATACACACACATACACACACATACACACACACACAAACACA

>AgamP3:2L:5234462:5235643:1

TGTGTTTGTGTGTGTGTATGTGTGTGTATGTGTATGTGTGTGTGCATGTGTGTGTGGATG

TATGTTTGAATGTGTATTGATGTGTGTGTTTGTTTCACAAGTAGGTCGTTTCGGATAGAT

TTGTAAGTAGTTTTTTTGTGTTTTGGGTTAGGTTTTTGGTGTGATAAGCAGGACCACCGC

CCTCGCGATCAGTGTGTTTTCGATATATTAACAATGTTACGGTCTTCTTTCGCCGTGGTC

TTCTGAGGACGTTCGGTTGACTTGCGCGTTTCGGTTGTCCAAGCGCGTTTCAGTTGTCTA

AGCACGTTTCGGTTGTCCGAAGCGCGTTTGCCACAAAAGTGCGCGATCGTTCCATTACGA

ACTCGATCCTTTTGCGCTTAATGCCAGCAGCAGCCATTCGCTTTTACATATGGCGCTGAT

GATCGGTACAACGTTTACCTCGACCCATTGTTACTAACATTGCTTGCTTGGACCGTCAAG

AACGCGCTGCTTAAAAACTTGGACACGGCTGATCCCGTGCTCTGCCTGCGTTATACTTCT

ATACGCGATGAATTACATCGTTTTGGAGCAGCGAAAACATCGCATGGATAAAAACTATCA

ACGAGTACGCGAGTAAGCGTCTTCCCTTCAAAATCGAGAAAAGAATACTGCCGCGCTCAT

GAAACAAAACGCCCATTGAAAAGAATAACTGCGCGCCAGCTCAATGCACGCACAAAGTTT

ACCATTCGCACACTACGTGCAACGCAGAGATGCACGCATGCCTTTCAATGGCGTGCACTG

GAGAAACACCTGTGAGGGAATGCCGAGTTCATTGCTATTGTGCGCGTGTCCATTTCGCAC

CGGTGTCGCATTCACATTCATGAAACAGCACACCTGCGTTTTCGTCCGAGGAACAAGCGC

TGCTGTCGATGCAAACTTTAGTTTTTATCCAAGTGTAAACGCACAATGTTTCACATGATA

ACACACATAATAAGAATAATTTCAACGCAATATGACATCATGGCAAGCTATGTTTTTCAG

ACACAAACCCGCGCACTTGCTAATACACATTAAAAAGCACACTCTCTTTCTACTACTACA

CACACACACACATGCACACACACACACACATGCACACACACACACATACACACACACACT

CACACACACAAACACACACATGCACACATACACACACACACA

>AgamP3:2L:876032:877233:1

TGTGTTTGTGTGTGTGTATGTGTGTATGTGTGTGTGTGTATGTGTTTGTGTGTGAGTGTG

TGTGTATGTGTGTGTGTGCATGTGTGTGTGTGTGTAGTAGTAGAAAGAGAGTGTGCTTTT

TAATGTGTATTTGCAAGTGCGCGGGTTTGTGTCTGAAAAACATAGCTTGCCATGATGTCA

TATTGCGTTGAAATTATTCTTATTATGTGTGTTATCATGTGAAACATTGTGCGTTTACAC

TTGGATAAAAACTAAAGTTTGCATCGACAGCAGCGCTTGTTCCTCGGACGAAAACGCAGG

TGTGCTGTTTCATGAATGTGAACGCGACACCGGTGCGAAATGGACACGCGCACAATAGCA

ATGAACTCGGCATTCCCTCACAGGTGTTTCTCCAGTGCACGCCATTGAAAGGCCTTCGTG

CATCTCTGCGTTGCACGTTGTGTGCGAATGGTAAACTTTGTGCGTGCATTGAGCTGGCGC

GCAGTTATTCTTTTCAATGGGCGTTTTGTTTCATGAGCGCGGCAGTATTCTTTTCTCGAT

TTTGAAGGGAAGACGCTTATTCGCGTACTCGTTGATAGTTTTTATCCATGCGATGTTTTC

GCTGCTCCAAAACGATGTAATTCATCGCCTATAGAAGTATAACGCAGGCAGAGCACGGCA

TCAGCCGTGTCCAAGTTTTTAACCAGCGCGTTCTTGACGGTCCAAGCAAGCAATATTAGT

AACAATAGGTCGAGGTAAACGTTGTACCGATTATCAGCGCCATATGTAAAAGCGAATGGC

TGCTGCTGGCATTAAGCGCAAAAGGATCGAGTTCGTAATGGAACGATCGCGCACTTTTGT

GGCAAACGCGCTTCGGACAACCGAAACGTGCTTAGACAACTGAAACGCGCTTGGACAACC

GAAACGCGCAAGTCAACCGGACGTCCTCAGAAGACCACGGCGAAAGAAGACCGTAACATT

GTTAATATATCGAAAACACACTGATCGCGAGGGCGGTGGTCCTGCTTATCACACCAAAAA

CCTAACCCAAAACACAAAAAAACTACTTACAAATCTATCCGAAACGACCTACTTGTGAAA

CAAACACACACATCAATACACATTCAAACATACATCCACACACACACACACACACACATG

CACACACACATACACACACACACACACACACACACATACACACACACACACACACAAACA

CA

>AgamP3:2R:13827228:13828509:1

TGTGTGTGTGTATGTGTGTATGTGTGTGTGTATGTGTGTGAGTGTGTGTGTGTGTGTGTG

TGTGTGTGTGTGTGTGTGTGTGTGTGTGTGTGTGTGTGTGTGTGTGTGTGTGTGTGTGTG

TGTGTGTGTGTGTGTGTGTGTTTGTGTGTGTGTGTGTGTTTGTGTGTGTGTGTGTGTGTG

TAGTAGTAGAAAGTGAGTGTGCTTTTTAATGTGTATTTGCAAGTGCGCGGGTTTGTGTCT

GAAAAACATAGCTTGCCATGATGTCATATTGCGTTGAAATTATTCTTATTATGTGTGTTA

TCATGTGAAACATTGTGCGTTTACACTTGGATAAAAACTAAAGTTTGCATCGACAGCAGC

CCTTGTTCCTCGGACGAAAACGCAGGTGTGCTGTTTCATGAATGTGAATGCGACACCGGT

GCGAAATGGACACACGCACAATAGCAATGAACTCGGCATTCTCTCACAGGTGTTTCTCCA

GTGCACGCCATTGAAAGGCCTTCGTGCATCTCTGCGTTGCACGTTGTGTGCGAATGGTAA

ACTTTGTGCGTGCATTGAGCTGGCGCGCAGTTGTTCTTTTCAATGGGCGTTTTGTTTCAT

GAGCGCGGCAGTATTATGTTCTCGATTTTGAAGGGAAGACGCTTACTCGCGTACTCGTTG

ATAGTTTTTATCCATGCGATGTTTTCGCTGCTCGACAACGATGTTATTCATCGCGTATAG

AAGTAGAACGCAGGCAGAGCACGGGATCAGCCGTGTCCAAGTTTTTAACCAGCGCGTTCT

TGACGGTCCAAGCAAGCAATGTTAGTAACAATGGGTCGAGGTAAACGTTGTACCGATTAT

CAGCGCCATATGTAAAAGCGAATGGCTGCTGCTGGCATTAAGCGCAAAAGGATCGAGTTC

GTAATGGAACGATCGCGCACTTTTGTGGCAAACGCGCTTCGGACAACCGAAACGTGCTTA

GACAACTGAAACGCGCTTGGACAACCGAAACGCGCAAGTCAACCGGTCGTCCTCAGAAGA

CCACGGCGAAAGAAGACCGTAACATTGTTAATATATCGAAAACACACTGATCGCGAGGGC

GGTGGTCCTGCTTATCACACCAAAAACCTAACCCAAAACACAAAAAAGCTACTTACAAAT

CTATCCGAAACGACCTACTTGTGAAACAAACACACACATCAATACACATTCAAACATACA

TCCACACACACACACACACACATGCACACACACATACACACACACATACACACACATACA

CACACACACAAACACAAACACA

>AgamP3:2R:39937762:39939021:1

TGTGTTTGTGTGTGTGTGTATGTGTGTGTATGTGTGTGTATGTGTGTTTGTGTGTGTGTG

TGTGTGTGTGTGTGTGTGTGTGTGTATATGTGTGTGTGCATGTGTGTGTGTGTGTGTGTG

GATGTATGTTTGAATGTGTATTGATGTGTGTGTTTGTTTCACAAGTAGGTCGTTTCAGAT

AGTTTTGTTAGTAGTTTTTTTGTGTTTTGGGTTAGGTTTTTGGTGTGATAAGCAGGACCA

CCGCCCTCGCGATCAGTGTGTTTTCGATGTATTAACAATGTTACGGTCTTCTTTCGCCGT

GGTCTTCTGAGGACGTCCGGTTGACTTGCGCGTTTCGGTTGTCCAAACGCGTTTCAGTTG

TCTAAGCACGTTTCGGTTGTCCGAAGCGCGTTTGCCACAAAAGTGCGCGATCGTTCCATT

ACGAACTCGATCCTTTTGCGCTTAATGCCAGCAGCAGCCATTCGCTTTTACATATGGCGC

TGATGATCGGTACAACGTTTACCTCGACCCATTGTTACTAACATTGCTTGCTTGGACCGT

CAAGAACGCACTGGTTAAAAACTTGGACACGGCTGATCCCGTGCTCTGCCTGCGTTCTAC

TTCTATACGCGATGAATTACATCGTTTTGGAGCAGCAAAAACATCGCATGGATAAAAACT

ATCAACGAGTACGCGAGTAAGCGTCTTCCCTTCAAAATCGAGAAAAGAATACTGCCGCGC

TCATGAAACAAAACGCCCATTGAAAAGAATAACTGCGCGCCAGCTCAATGCACGCACAAA

GTTTACCATTCGCACACAACGTGCAACGCAGAGATGCACGAAGGCCTTTCAATGGCGTGC

ACTGGAGAAACACCTGTGAGGGAATGCCGAGTTCATTGCTATTGTGCGCGTGTCCATTTC

GCACCGGTGTCGCATTCACATTCATGAAACAGCACACCTGCGTTTTCGTCCGAGGAACAA

GCGCTGCTGTCGATGCAAACTTTAGTTTTTATCCAAGTGTAAACGCACAATGTTTCACAT

GATAACACACATAATAAGAATAATTTTAACGCAATATGACATCATGGCAAGCTATGTTTT

TCAGACACAAACCCGCGCACTTGCTAATACACATTAAAAAGCACACTCTCTTTCTACTAC

TACACACACACACACATGCACACACACACACACACATACACACACACACTCACACACACA

CACACACACACACTCACACACATACACACACACATACACACATACACACACACAAACACA

>AgamP3:2R:4134771:4135970:1_genic

TGTGTTTGTGTGTGTGTATGTGTGTATGTGTGTGTGTATGTGTGTGTGTGAGTGTGTGTG

TATGTGTGTGTGTGTGCATGTGTGTGTGTGTGTAGTAGTAGAAAGAGAGTGTGCTTTTTA

ATGTGTATTTGCAAGTGCGCGGGTTTGTGTCTGAAAAACATAGCTTGCCATGATGTCATA

TTGCGTTGAAATTATTCTTATTATGTGTGTTATCATGTGAAACATTGTGCGTTTACACTT

GGATAAAAACTAAAGTTTGCATCGACAGCAGCGCTTGTTCCTCGGACGAAAACGCAGGTG

TGCTGTTTCATGAATGTGAACGCGACACCGGTGCGAAATGGACACGCGCACAATAGCAAT

GAACTCGGCATTCCCTCACAGGTGTTTCTCCAGTGCACGCCATTGAAAGGCCTTCGTGCA

TCTCTGCGTTGCACGTTGTGTGCAAATGGTAAACTTTGTGCGTGCATTGAGCTGGCGCGC

AGTTATTCTTTTCAATGGGCGTTTTGTTTCATGAGCGCGGCAGTATTCTTTTCTCGATTT

TTAAGGGAAGACGCTTATTCGCGTACTCGTTGATAGTTTTTATCCATGCGATGTTTTCGC

TGCTCCAAAACGATGTAATTCATCGCGTATAGAAGTATAACGCAGGCAGAGCACGGGATC

AGCCGTGTCCAAGTTTTTAACCAGCGCGTTCTTGACGGTCCAAGCAAGCAATATTAGTAA

CAATAGGTCGAGGTAAACGTTGTACCGATTATCAGCGCCATATGTAAAAGCGAATGGCTG

CTGCTGGCATTAAGCACAAAAGGATCGAGTTCGTAATGGAACGATCGCGCACTTTTGTGG

CAAATGCGCTTCGGGCAACCGAAACGTGCTTAGACAACTGAAACGCGCTTGGACAACCGA

AACGCGCAAGTCAACCGGACGTCCTCAGAAGACCACGTCGAAAGAAGACCGTAACATTGT

TAATATATCGAAAACACACTGATCGCGAGGGCGGTGGTCCTGCTTATCACACCAAAAACC

TAACCCAAAACACAAAAAAACTACTTACAAATCTATCCGAAACGACCTACTTGTGAAACA

AAAACACACATCAATACACATTCAAACATACATCCACACACACACACACACATGCACACA

CACATACACACACACACACACACACACACACACACACACACACACACACACATACACACA

>AgamP3:2R:44506494:44507699:1

TGTGTTTGTGTGTGTGTGTGTATGTGTGTGTATGTGTGTGTGTGTGTGTGTATGTGTGTG

TGCATGTGTGTGTGTGTGTGGATGTATGTTTGAATGTGTATTGATGTGTGTGTTTGTTTC

ACAAGTAGGTCGTTTCGGATAGATTTGTAAGTAGCTTTTTTGTGTTTTGGGTTAGGTTTT

TGGTGTGATAAGCAGGACCACCGCCCTCGCGATCAGTGTGTTTTCGATATATTAACAATG

TTACGCTCTTCTTTCGCCGTGGTCTTCTGAGGACGACCGGTTGACTTGCGCGTTTCGGTT

GTCCAAGCGCGTTTCAGTTGTCTAAGCACGTTTCGGTTGTCCGAAGCGCGTTTGCCACAA

AAGTGCGCGATCGTTCCATTACGAACTCGATCCTTTTGCGCTTAATGCCAGCAGCAGCCA

TTCGCTTTTACATATGGCGCTGATAATCGGTACAACGTTTACCTCGACCCATTGTTACTA

ACATTGCTTGCTTGGACCGTCAAGAACGCGCTGGTTAAAAACTTGGACACGGCTGATCCC

GTGCTCTGCCTGCGTTCTACTTCTATACGCGATGAATTACATCGTTGTCGAGCAGCGAAA

ACATCGCATGGATAAAAACTATCAACGAGTACGCGAGTAAGCGTCTTCCCTTCAAAATCG

AGAACAGAATACTGCCGCGCTCATGAAACAAAACGCCCATTGAAAAGAACAACTGCGCGC

CAGCTCAATGCACGCACAAAGTTTACCATTCGCACACAACGTGCAACGCAGAGATGCACG

AAGGCCTTTCAATGGCGTGCACTGGAGAAACACCTGTGAGAGAATGCCGAGTTCATTGCT

ATTGTGCGTGTGTCCATTTCGCACCGGTGTCGCATTCACATTCATGAAACAGCACACCTG

CGTTTTCGTCCGAGGAACAAGGGCTGCTGTCGATGCAAACTTTAGTTTTTATCCAAGTGT

AAACGCACAATGTTTCACATGATAACACACATAATAAGAATAATTTCAACGCAATATGAC

ATCATGGCAAGCTATGTTTTTCAGACACAAACCCGCGCACTTGCAAATACACATTAAAAA

GCACACTCACTTTCTACTACTACACACACACACATGCACACACACACACACACAAACACA

CACACACACACACACACACACACACTCACACACATACACACACACATACACACATACACA

CACACA

>AgamP3:2R:59239666:59240821:1

TGTGTATGTGTGTGTGTATGTGTGTGTGTGTGTGTGTGTGTGTGCATGTGTGTGTGTGTA

GTAGTAGAAAGAGAGTGTGCTTTTTAATGTGTATTTGCAAGTGCGCGGGTTTGTGTCTGA

AAAACATAGCTTGCCATGATGTCATGTGGCGTTGAAATTATTCTTATTATGTGTGTTATC

ATGTGAAACATTGTGCGTTTGCACTTGGATAAAAACTAAAGTTTGCATCGACAGCAGCGC

TTGTTCCTCGGACGAAAACGCAGGTGTGCTGTTTAATGAATGTGAATGCGACACCGGTGC

GAAATGGACACGCGCACAATAGCAATGAACTCGGCATTCCCTCACAGGTGTTTCTCCAGT

GCACGCCATTGAAAGGCCTTCGTGCATCTCTGCGTTGCACGTTGTGTGCGAATGGTAAAC

TTTGTGCGTGCATTGAGCTGGTGCGCAGTTGTTCTTTTCAATGGGCGTTTTGTTTCATGA

GCGCGGCAGTATTCTTTTCTCGATTTTGAAGGGAAGACGCTTACTCGCGTACTCGTTGAT

AGTTTTTATCCATGCGATGTTTTCGCTGCTCCAAAACGATGTAATTCATCGCGTATAGAA

GTAGAACGCAGGCAGAGCACGGGATCAGCCGTGTCCAAGTTTTTAACCAGCGCGTTCTTG

ACGGTCCAAGCAAGCAATGTTAGTAACAATGGGTCGAGGTAAACGTTGTACCGATTATCA

GCGCCATATGTAAAAGCGAATGGCTGCTGCTGGCATTAAGTGCAAAAGGATCGAGTTCGT

AATGGAACGATCGCGCACTTTTGTGGCAAACGCGCTTCGGACAACCGAAACGTGCTTAGA

CAACTGAAACGCGCTTGGACAACCGAAACGCGCAAGTCAACCGGACGTCCTCAGAAGACC

ACGGCGAAAGAAGACCGTAACATTGTTAATATATCAAAAACACACTGATCGCGAGGGCGG

TGGTCCTGCTTATCACACCAAAAACCTAACCCAAAACACAAAATAGCTACTTACAAATCT

ATCCGAAACGACCTACTTGTGAAACAAACACACACATCAATACACACTCAAACATACATC

CACACACACACACACATGCACACACACATACACACACACACATACACACACATACACACA

CACAAACACAAACACA

>AgamP3:2R:60013531:60014758:1

TGTGTTTGTGTGTGTGTATGTGTGTATGTGTGTGTGTATGTGTGTATGAGTGTGTGTGTG

TGTATGTGTGTGTGTCTGTGTGTGTGCATGTGTGTGTGTGTGTAGTAGTAGAAAGAGAGT

GTGCTTTTTAATGTGTATTTGCAAGTGCGCGGGTTTGTGTCTGAAATACATAGCTTGCCA

TGATGTCATATTGCGTTGAAATTATTCTTATTATGTGTGTTATCATGTGAAACATTGTGC

GTTTACACTTGGATAAAAACTAAAGTTTGCATCGACAGCAGCGCTTGTTCCTCGGACGAA

AACGCAGGTGTGCTGTTTCATGAATGTGAATGCGACACCGGTGCGAAATGGACACGCGCA

CAATAGCAATGAACTCGGCATTCCCTCACAGGTGTTTCTCCAGTGCACGCCATTGAAAGG

CCTTCGTGCATCTCTGCGTTGCACGTTGTGTGCGAATGGTAAACTTTGTGCGTGCATTGA

GCTGGCGCGCAGTTGTTCTTTTCAATGGGCGTTTTGTTTCATGAGCGCGGCAGTATTCTT

TTCTCGATTTTGAAGGGAAGACGCTTACTCGCGTACTCGTTGATAGTTTTTATCCATGCG

ATGTTTTCGCTGCTCCATAACGATGTAATTCATCGCGTATAGAAGTAGAACGCAGGCAGA

GCACGGGATCAGCCGTGTCCAAGTTTTTAACCAGCGCGTTCTTGACGGTCCAAGCAAGCA

ATGTTAGTAACAATGGGTCGAGGTAAACGTTGTACCGATCATCAGCGCCATATGTAAAAG

CGAATGGCTGCTGCTGGCATTAAGCGCAAAAGGATCGAGTTCGTAATGGAACGATCGCGC

ACGTTTGTGGCAAACGCGCTTCGGACAACCGAAACGTGCTTAGACAACTGAAACGCGCTT

GGACAACCGAAACGCGCAAGTCAACCGGACGTCCTCAGAAGACCACGGCGAAAGAAGACC

GTAACATTGTTAATATATCGAAAACACACTGATCGCGAGGGCGGTGGTCCTGCTTATCAC

ACCGAAAACCTAACCCAAAACACAAAAAAACTACTTACAAATCTATCCGAAACGACCTAC

TTGTGAAACAAACACACACATCAATACACATTCAAACATACATCCACACACACACACACA

TGCACACACACATACACACACACACACACACACACACACACACAAACACACATACACACA

CATACACACACACACAAACACAAACACA

>AgamP3:2R:61342850:61344119:1

TGTGTTTGTGTGTGTATGTGTGTATGTGTGTGTATGTGTGTTTGTGTGTATGTGTGTGTG

TGTGTGTGTGTGTGTGTGTGTGTGTGTGTGTGTGTGTGTGTGTGTGTGTGTGTGTGTGTG

TGTGTGTGTGGATGTATGTTTGAATGTGTATTGATGTGTGTGTTTGTTTCACAAGTAGGT

CGTTTCGGATAGATTTGTAAGTAGTTTTTTTGTGTTTTGAGTTAGGTTTTTGGTGTGATA

AGCAGGACCACCGCCCTCGCGATCAGTGTGTTTTCGATATATTAACAATGTTACGGTCTT

CTTTCGCCGTGGTCTTCTGAGGACGTCCGGTTAACTTGCGCGTTTCGGTTGTCCAAGCGC

GTTTCAGTTGTCTAAGCACGTTTCGGTTGTCCGAAGCGCGTTTGCCACAAAAGTGCGCGA

TCGTTCCATTACGAACTCGATCCTTTTGCGCTTAATGCCAGCAGCTGCCATTCGCTTTTA

CATATGGCACTGATGATCGGTACAACGTTTACCTCGACCCATTGTTACTAACATTGCTTG

CTTGGACCGTCAAGAACGTGCTGGTTAAAAACTTGGACACGGCTGATCCCGTGCTCTGCC

TGCGTTCTACTTCTATACGCGATGAATTACATCGTTTTGGAGCAGCGAAAACATCGCATC

GATAAAAACTATCAACGAGTACGCGAGTAAGCGTCTTCCTTTCAAAATCGAGAAAAGAAT

ACTGCCGCGCTCATGAAACAAAACGCCCATTGAAAAGAATAACAGCGCGCCAGCTCAATG

CACGCACAAAGTTTACCATTCGCACACAACGTGCAACGCAGAGATGCACGAAGGCCTTTC

AATGGCGTGCACTGGAGAAACACCTGTGAGGGAATGCCGAGTTCATTGCTATTGTGCGCG

TGTCCATTTCGCACCGGTGTCGCATTCACAGTCATGAAACAGCACACCTGCGTTTTCGTC

CGAGGAACAAGCGCTGCTGTCGATGCAAACTTTAGTTTTTATCCAAGTGTAAACGCACAA

TGTTTCACATGATAACACACATAACAAGAATAATTTCAACGCAATATGACATCATGGCAA

GCTATGTTTTTCAGACACAAACCCGCGCACTTGCTAATACACATTAAAAAGCACACTCTC

TTTCTACTACTACACACACACACACATGCACACACACACACAAACACACACACACACACA

CACACACACACACACACACACACACACACACACACACACACACACACACACATACACACA

CACAAACACA

>AgamP3:3L:10536049:10537278:1_genic

TGTGTGTGTGTATGTGTGTATGTGTGTGTATGTGTGTGAGTGTGTGTGTGTGTGTGTGTG

TGTGTGTGTGTGTGTGTGTGTGTGTTTGTGTGTGTGTGTGTGTGTGTGTGTGTGTGCATG

TGTGTGTGTGTAGTAGTAGAAAGTGAGTGTGCTTTTTAATGTGTATTTGCAAGTGCGCGG

GTTTGTGTCTGAAAAACATAGCTTGCCATGATGTCATATTGCGTTGAAATTATTCTTATT

ATGTGTGTTATCATGTGAAACATTGTGCGTTTACACTTGGATAAAAACTAAAGTTTGCAT

CGACAGCAGCCCTTGTTCCTCGGACGAAAACGCAGGTGTGCTGTTTCATGAATGTGAATG

CGACACCGGTGCGAAATGGACACACGCACAATAGCAATGAACTCGGCATTCTCTCACAGG

TGTTTCTCCAGTGCACGCCATTGAAAGGCCTTCGTGCATCTCTGCGTTGCACGTTGTGTG

CGAATGGTAAACTTTGTGCGTGCATTGAGCTGGCGCGCAGTTGTTCTTTTCAATGGGCGT

TTTGTTTCATGAGCGCGGCAGTATTCTGTTCTCGATTTTGAAGGGAAGACGCTTACTCGC

GTACTCGTTGATAGTTTTTATCCATGCGATGTTTTCGCTGCTCGACAACGATGTTATTCA

TCGCGTATAGAAGTAGAACGCAGGCAGAGCACGGGATCAGCCGTGTCCAAGTTTTTAACC

AGCGCGTTCTTGACGGTCCAAGCAAGCAATGTTAGTAACAATGGGTCGAGGTAAACGTTG

TACCGATTATCAGCGCCATATGTTAAAGCGAATGGCTGCTGCTGGCATTAAGCGCAAAAG

GATCGAGTTCGTAATGGAACGATCGCGCACTTTTGTGGCAAACGCGCTTCGGACAACCGA

AACGTGCTTAGACAACTGAAACGCGCTTGGACAACCGAAACGCGCAAGTCAACCGGTCGT

CCTCAGAAGACCACGGCGAAAGAAGACCGTAACATTGTTAATATATCGAAAACACACTGA

TCGTGAGGGCGGTGGTCCTGCTTATCACACCAAAAACCTAACCCAAAACACAAAAAAGCT

ACTTACAAATCTATCCGAAACGACCTACTTGTGAAACAAACACACACATCAATCCACATT

CAAACATACATCCACACACACACACACATGCACACACACATACACACACACATACACACA

CATACACACACACACACAAACACAAACACA

>AgamP3:3L:16545413:16546596:1

TGTGTTTGTGTTTGTGTGTGTGTGTGTATGTGTGTGTATGTGTGTGTGCATGTGTGTGTG

TGTGTGTGGATGTATGTTTGAATGTGTATTGATGTGTGTGTTTGTTTCACAAGTAGGTCG

TTTCGGATAGATTTGTAAGTAGCTTTTTTGTGTTTTGGGTTAGGTTTTTGGTGTGATAAG

CAGGACCACCGCCCTCGCGATCAGTGTGTTTTCGATATATTAACAATGTTACGGTCTTCT

TTCGCCGTGGTCTTCTGAGGACGACCGGTTGACTTGCGCGTTTCGGTTGTCCAAGCGCGT

TTCAGTTGTCTAAGCACGTTTCGGTTGTCCGAAGCGCGTTTGCCACAAAAGTGCGCGATC

GTTCCATTACGAACTCGATCCTTTTGCGCTTAATGCCAGCAGCAGCCATTCGCTTTTACA

TATGGCGCTGATAATCGGTACAACGTTTACCTCGACCCATTGTTACTAACATTGCTTGCT

TGGACCGTCAAGAACGCGCTGGTTAAAAACTTGGACACGGCTGATCCCGTGCTCTGCCTG

CGTTCTACTTCTATACGCGATGAATAACATCGTTGTCGAGCAGCGAAAACATCGCATGGA

TAAAAACTATCAACGAGTACGCGAGTGAGCGTCTTCCCTTCAAAATCGAGAACAGAATAC

TGCCGCGCTCATGAAACAAAACGCCCATTGAAAAGAACAACTGCGCGCCAGCTCAATGCA

CGCACAAAGTTTACCATTCGCACACAACGTGCAACGCAGAGATGCACGAAGGCCTTTCAA

TGGCGTGCACTGGAGAAACACCTGTGAGAGAATGCCGAGTTCATTGCTATTGTGCGTGTG

TCCATTTCGCACCGGTGTCGCATTCACATTCATGAAACAGCACACCTGCGTTTTCGTCCG

AGGAACAAGGGCTGCTGTCGATGCAAACTTTAGTTTTTATCCAAGTGTAAACGCACAATG

TTTCACATGATAACACACATAATAAGAATAATTTCAACGCAATATGACATCATGGCAAGC

TATGTTTTTCAGACACAAACCCGCGCACTTGCAAATACACATTAAAAAGCACACTCACTT

TCTACTACTACACACACACACATGCACACACACACACACACACACAAACACACACACACA

CACTCACACACATACACACACACATACACACATACACACACACA

>AgamP3:3L:23810005:23811167:1

TGTGTTTGTGTTTGTGTGTGTGTGTATGTGTGTGTATGTGTGTTTGTGTGTGTGTGTGTA

TGTGTGTGTGCATGTGTGTGTGTGTGTGGATGTATGTTTGAATGTGTATTGATGTGTGTG

TTTGTTTCACAAGTAGGTCGTTTCGGATAGATTTGTAAGTAGTTTTTTTGTGTTTTGGGT

TAGGTTTTCGGTGTGATAAGCAGGACCACCGCCCTCGCGATCAGTGTGTTTTCGATATAT

TAACAATGTTACGGTCTTCTTTCGCCGTGGTCTTCTGAGGACGTCCGGTTGACTTGCGCG

TTTCGGTGGTCCAAGCGCGTTTCAGTTGTCTAAGCACGTTTCGGTTGTCCGAAGCGCGTT

TGCCACAAAAGTGCGCGATCGTTCCATTACGAACTCGATCCTTTTGCGCTTAATGCCAGC

AGCAGCCATTCGCTTTTACATATGGCGCTGATGATCGGTACAACGTTTACCTCGACCCAT

TGTTACTAACATTGCTTGCTTGGACCGTCAAGAACGCGCTGGTTAAAAACTTGGACACGG

CTGATCCCGTGCTCTGCCTGCGTTCTACTTCTATACGCGATGAATTACATCGTTATGGAG

CAGCGAAAACATCGCATGGATAAAAACTATCAACGAGTACGCGAGTAAGCGTCTTCCCTT

CAAAATCGAGAAAAGAATACTTCCGCGCTCATGAAACAAAACGCCCATTGAAAAGAATAA

CTACGCGCCAGCTCAATGTACGCACAAAGTTTACCATTCGCACACAACGTGCAACGCAGA

GATGCACGAAGGCCTTTCAATGGCGTGCACTGGAGAAACACCTGTGAGGGAATGCCGAGT

TCATTGCTATTGTGCGCGTGTCCATTTCGCACCGGTGTCGCATTCACATTCATGAAACAG

CACACCTGCGTTTTCGTCCGAGGAACAAGCTCTGCTGTCGATGCAAACTTTAGTTTTTAT

CCAAGTGTAAACGCACAATGTTTCACATGATAACACACATAATAAGAATAATTTTAACGC

AATATGACATCATGGCAAGCTATGTATTTCAGACACAAACCCGCGCACTTGCAAATACAC

ATTAAAAAGCACACTCTCTTTCTACTACTACACACACACACATGCACACACACAGACACA

CACACATACACACACACACACAC

>AgamP3:3L:2533284:2534497:1

TGTGTTTGTGTGTGTGTGTGTATGTGTGTGTATGTGTGTGTGTGTGTATGTGTGTGTGCA

TGTGTGTGTGTGTGTGTGTGTGGATGTATGTTTGAATGTGTATTGATGTGTGTGTTTGTT

TCACAAGTAGGTCGTTTCGGATAGATTTGTAAGTAGCTTTTTTGTGTTTTGGGTTAGGTT

TTAGGTGTGATAAGCAGGACCACCGCCCTCGCGATCAGTGTGTTTTCGATATATTAACAA

TGTTACGGTCTTCTTTCGCCGTGGTTTTCTGAGGACGACCGGTTGACTTGCGCGTTTCGG

TTGTCCAAGCGCGTTTCAGTTGTCTAAGCACGTTTCGGTTGTCCGAAGCGCGTTTGCCAC

AAAAGTGCGCGATCGTTCCATTACGAACTCGATCCTTTTACGCTTAATGCCAGCAGCAGC

CATTCGCTTTTACATATGGCGCTGATGATCGGTACAACGTTTACCTCGACCCATTGTTAC

TAACATTGCTTGCTTGGACCGTCAAAAACGCACTGGTTAAAAACTTGGACACGGCTGATC

CCGTGCTCTGCCTGCGTTCTACTTCTATACGCGATGAATAACATCGTTGTCGAGCAGCGA

AAACATCGCATGGATAAAAACTATCAACGAGTACGCGAGTAAGCGTCTTCCCTTCAAAAT

CGAGAACAGAATACTGCCGCGCTCATGAAACAAAACGCCCATTGAAAAGAACAACTGCGC

GCCAGCTCAATGCACGCGCAAAGTTTACCATTCGCACACAACGTGCAACGCAGAGATGCA

CGAAGGCCTTTCAATGGCGTGCACTGGAGAAACACCTGTGAGAGAATGCCGAGTTCATTG

CTTTTGTGCGTGTGTCCATTTCGCACCGGTGTCGCATTCACATTCATGAAACAGCACACC

TGCGTTTTCGTCCGAGGAACAAGGGCTGCTGTCGATGCAAACTTTAGTTTTTGTCCAAGT

GTAAACGCACAATGTTTCACATGATAACACACATAATAAGAATAATTTCAACGCAATATG

ACATCATGGCAAGCTATGTTTTTCAGACACAAACCCGCGCACTGGCAAATACACATTAAA

AAGCACACTCACTTTCTACTACTACACACACACACATGCACACACACACACACACACACA

CACACACAAACACACATACACACACACACACACTCACACACATACACACACACATACACA

CATACACACACACA

>AgamP3:3L:32995160:32996343:1

TGTGTGTGTGTATGTGTGTATGTGTGTGTGTATGTGTGTGAGTGTGTGTGTGTGTGTGTG

TGTGTGTGTTTGTGTGTGTGTGTGTGTGTGTGTGTGTGTGTGTGTGTGTGTGTGTGTGTG

TGTGTGTGCATGTGTGTGTGTGTGTGTAGTAGTAGAAAGTGAGTGTGCTTTTTAATGTGT

ATTTGCAAGTGCGCGGGTTTGTGTCTGAAAAACATGGCTTGCCATGATGTCATATTGCGT

TGAAATTATTCTTATTATGTGTGTTATCATGTGAAACATTGTGCGTTTACACTTGGATAA

AAACTAAAGTTTGCATCGACAGCAGCCCTTGTTCCTCGGACGAAAACGCAGGTGTGCTGT

TTCATGAATGTGAATGCGACACCGGTGCGAAATGGACACACGCACAATAGCAATGAACTC

GGCATTCTCCTACAGGTGTTTCTCCAGTGCACGCCATTGAAAGGCCTTCGTGCATCTCTG

CGTTGCACGTTGTGTGCGAATGGTAAACTTTGTGCGTGCATTGAGCTGGCGCGCAGTTGT

TCTTTTCAATGGGCGTTTTGTTTCACGAGCGCGGCAGTATTCTGTTCTCGATTTTGAAGG

GAAGACGCTTACTCGCGTACTCGTTGATAGTTTTTATCCATGCGATGTTTTCGCTGCTCG

ACAACGATGTAATTCATCGCGTATAGAAGTAGAACGCAGGCAGAGCACGGGATCAGCCGT

GTCCAAGTTTTTAACCAGCGCGTTCTTGACGGTCCAAGCAAGCAATGTTAGTAACAATGG

GTCGAGGTAAACGTTGTACCGATTATCAGCGCCATATGTAAAAGCGAATGGCTGCTGCTG

GCATTAAGCGCAAAAGGATCGAGTTCGTAATGGAACGATCGCGCACTTTTGTGGCAAACG

CGCTTCGGACAACCGAAACGTGCTTAGACAACTGAAACGCGCTTGGACAACCGAAACGCG

CAAGTCAACCGGACGTCCTCAGAAGACCACGGCGAAAGAAGACCGTAACATTGTTAATAT

ATCGAAAACACACTGATCGCGAGGGCGGTGGTCCTGCTTATCACACCAAAAACCTAACCC

AAAACACAAAAAAGCTACTTACAAATCTATCCGAAACGACCTACTTGTGAAACAAACACA

CACATCAATACACATTCAAACATACATCCACACACACACACACA

>AgamP3:3L:33127359:33128564:1

TGTGTTTGTGTGTGTGTATGTGTGTATGTGTGTGTGTGTATGTGTGTGAGTGTGTGCATG

TGTGTGTGTGTGTGTAGTAGTAGAAAGAGAGTGTGCTTTTTAATGTGTATTAGCAAGTGC

GCGGGTTTGTGTCTGAAAAACATAGCTTGCCATGATGTCATATTGCGTTGAAATTATTCT

TATTATGTGTGTTATCATGTGAAACATTGTGCGTTTACACTTGAATAAAAACTAAAGTTT

GCATCGACAGCAGCGCTTGTTCCTCGGACGAAAACGCAGGTGTGCTGTTTCATGAATGTG

AATGCGACACCGGTGCGAAATGGACACGCGCACAATAGCAATGAACTCGGCATTCCCTCA

CAGGTGTTTCTCCAGTGCACGCCATTGAAAGGCCTTCGTGCATCTCTGCGTTGCACGTTG

TGTGCGAATGGTAAACTTTGTGCGTGCATTGAGCTGGCGCGCAGTTATTCTTTTCAATGG

GCGTTTTGTTTCATGAGCGCGGCAGTATTCTTTTCTCGATTTTGAAGGGAAGACGCTTAC

TCGCGTACTCGTTGATAGTTTTTATCCATGCGATGTTTTTGCTGCTCCAAAACGATGTAA

TTCATCGCGTATAGAAGTAGAACGCAGGCAGAGCACGGGATCAGCCGTGTCCAAGTTTTT

AACCAGTGCGTTCTTGACGGTCCAAGCAAGCAATGTTAGTAACAATGGGTCGAGGTAAAC

GTTGTACCGATCATCAGCGCCATATGTAAAAGCGAATGGCTGCTGCTGGCATTAAGCGCA

AAAGGATCGAGTTCGTAATGGAACGATCGCGCACTTTTGTGGCAAACGCGCTTCGGACAA

CCGAAACGTGCTTAGACAACTGAAACGCGTTTGGACAACCGAAACGCGCAAGTCAACCGG

ACGTCCTCAGAAGACCACGGCGAAAGAAGACCGTAACATTGTTAATACATCGAAAACACA

CTGATCGCGAGGGCGGTGGTCCTGCTTATCACACCAAAAACCTAACCCAAAACACAAAAA

AACTACTTACAAATCTATTCGAAACGACCTACTTGTGAAACAAACACACACATCAATACA

CATTCAAACATACATCCACACACACACACACACACATGCACACACACATATACACACACA

CACACACACACACACACACACACAAACACACATACACACACATACACACACATACACACA

CACACA

>AgamP3:3L:35915786:35917039:1

TGTGTTTGTGTTTGTGTGTGTGTGTGTATGTGTGTGTGTATGTGTGTTTGTGTGTGTGTG

TGTGTGTGTGTGTGTGTGTGTGTGTGTGTGTGTGTATGTGTGTGTGCATGTGTGTGTGTG

TGTGTGGATGTATGTTTGAATGTGTATTGATGTGTGTGTTTGTTTCACAAGTAGGTCGTT

TCGGATAGATTTGTAAGTAGTTTTTTTGTGTTTTGGGTTAGGTTTTCGGTGTGATAAGCA

GGACCACCGCCCTCGCGATTAGTGTGTTTTCGATATATTAACAATGTTACGGTCTTCTTT

CGCCGTGGTCTTCTGAGGACGTCCGGTTGACTTGCGCGTTTCGGTTGTCCAAGCGCGTTT

CAGTTGTCTAAGCACGTTTCGGTTGTCCGAAGCGCGTTTGCCACAAAAGTGCGCGATCGT

TCCATTACGAACTCGATCCTTTTGCGCTTAATGCCAGCAGCAGCCATTCGCTTTTACATA

TGGCGCTGATGATCGGTACAACGTTTACCTCGACCCATTGTTACTAACATTGCTTGCTTA

GACCGTCAAGAACGCGCTGGTTAAAAACTTGGACACGGCTGATCCCGTGCTCTGCCTGCG

TTCTACTTCTATACGCGATGAACTACATCGTTATGGAGCAGCGAAAACATCGCATGGATA

AAAACTATCAACGAGTACGCGAGTAAGCGTCTTCCCTTCAAAATCGAGAAAAGAATACTG

CCGCGCTCATGAAACAAAACGCCCATTGAAAAGAACAACTGCGCGCCAGCTCAATGAACG

CACAAAGTTTACCATTCGCACACAACGTGCAACGCAGAGATGCACGAAGGCCTTTCAATG

GCGTGCACTGGAGAAACACCTGTGAGGGAATGCCGAGTTCATTGCTATTGTGCGTGTGTC

CATTTCGCACCGGTGTCGCATTCACATTCATGAAACAGCACACCTGCGTTTTCGTCCGAG

GAACAAGGGCTGCTGTCGATGCAAACTTTAGTTTTTATCCAAGTGTAAACGCACAATGTT

TCACATGATAACACACATAATAAGAATAATTTCAACGCAATATGACATCATGGCAAGCTA

TGTTTTTCAGACACAAACCCGCGCACTTGCAAATACACATTAAAAAGCACACTCACTTTC

TACTACTACACACACACACATGCACACACACACACACAAACACACACACACACAAACACA

CACACACACACACTCACACACTCACACACATACACACACACATACACACACACA

>AgamP3:3L:9317489:9318748:1

TGTGTTTGTGTGTGTGTGTATGTGTGTGTATGTGTGTGTATGTGTGTTTGTGTGTGTGTG

TGTGTGTGTGTGTGTGTGTGTGTGTATATGTGTGTGTGCATGTGTGTGTGTGTGTGTGTG

GATGTATGTTTGAATGTGTATTGATGTGTGTGTTTGTTTCACAAGTAGGTCGTTTCAGAT

AGTTTTGTTAGTAGTTTTTTTGTGTTTTGGGTTAGGTTTTTGGTGTGATAAGCAGGACCA

CCGCCCTCGCGATCAGTGTGTTTTCGATGTATTAACAATGTTACGGTCTTCTTTCGCCGT

GGTCTTCTGAGGACGTCCGGTTGACTTGCGCGTTTCGGTTGTCCAAACGCGTTTCAGTTG

TCTAAGCACGTTTCGGTTGTCCGAAGCGCGTTTGCCACAAAAGTGCGCGATCGTTCCATT

ACGAACTCGATCCTTTTGCGCTTAATGCCAGCAGCAGCCATTCGCTTTTACATATGGCGC

TGATGATCGGTACAACGTTTACCTCGACCCATTGTTACTAACATTGCTTGCTTGGACCGT

CAAGAACGCACTGGTTAAAAACTTGGACACGGCTGATCCCGTGCTCTGCCTGCGTTCTAC

TTCTATACGCGATGAATTACATCGTTTTGGAGCAGCAAAAACATCGCATGGATAAAAACT

ATCAACGAGTACGCGAGTAAGCGTCTTCCCTTCAAAATCGAGAAAAGAATACTGCCGCGC

TCATGAAACAAAACGCCCATTGAAAAGAATAACTGCGCGCCAGCTCAATGCACGCACAAA

GTTTACCATTCGCACACAACGTGCAACGCAGAGATGCACGAAGGCCTTTCAATGGCGTGC

ACTGGAGAAACACCTGTGAGGGAATGCCGAGTTCATTGCTATTGTGCGCGTGTCCATTTC

GCACCGGTGTCGCATTCACATTCATGAAACAGCACACCTGCGTTTTCGTCCGAGGAACAA

GCGCTGCTGTCGATGCAAACTTTAGTTTTTATCCAAGTGTAAACGCACAATGTTTCACAT

GATAACACACATAATAAGAATAATTTCAACGCAATATGACATCATGGCAAGCTATGTTTT

TCAGACACAAACCCGCGCACTTGCTAATACACATTAAAAAGCACACTCTCTTTCTACTAC

TACACACACACACACATGCACACACACACACACACATACACACACACACTCACACACACA

CACACACACACACTCACACACATACACACACACATACACACATACACACACACAAACACA

>AgamP3:3R:12823084:12824255:1_genic

TGTGTGTGTATGTGTGTGTGTGTATGTGTGTGTGCATGTGTGTGGGTGTGTGGATGTATG

TTTGAATGTGTATGGATGTGTTTGTTTGTTTCACAAGTAGGTCGTTTCGGATAGATTTGT

AAGTAGCTTTTTTGTGTTTTGGGTTAGGTTTTTGGTGTGATAAGCAGGACCACCGCCCTC

GCGATCAGTGTGTTTTCGATATATTAACAATGTTACGGTCTTCTTTCGCCGTGGTCTTCT

GAGGACGTCCGGTTGACTTGCGCGTTTCGGTTGTCCAAGCGCGTTTCAGTTGTCTAAGCA

CGTTTCGGTTGTCCGAAGCGCGTTTGCCACAAAAGTGCGCGATCGTTCCATTACGAACTC

GATCCTTTTGCGCTTAATGCCAGCAGCAGCCATTCGCTTTTACATATGGCGCTGATAATC

GGTACAACGTTTACCTTGACCCATTGTTACTAACATTGCTTGCTTGGACCGTCAAGAACG

CGCTGGTTAAAAACTTGGACACGGCTGATCCCGTGCTCTGCCTGCGTTATACTTCTATAC

GCGATGCATTACATCGTTTTGGAGCAGCGAAAACATCGCATGGATAAAAACTATCAACGA

GTATGCGAATAAGCGTCTTCCCTTCAAAATCGAGAAAAGAATACTGCCGCGCTCATGAAA

CAAAACGCCCATTGAAAAGAATAACTGCGCGCCAGCTCAATACACGCACAAAGTTTACCA

TTCGCACACAACGTGCAACGCAGAGATGCACGAAGGCCTTTCAATGGCGTGCACTGGAGA

AACACCTGTGAGAGAATGCCGAGTTCATTGCTATTGTGCGTGTGTCCATTTCGCACCGGT

GTCGCATTCACATTCATGAAACAGCACACCTGCGTTTTCGTCCGAGGAACAAGGGCTGCT

GTCGATGCAAACTTTAGTTTTTATCCAAGTGTAAACGCACAATGTTTCACATGATAACAC

ACATAATAAGAACAATTTCAACGCAATATGACATCATGGCAAGCTATGTTTTTCAGACAC

AAACCCGCGCACTTGCAAATACACATTAAAAAGCACACTCACTTTCTACTACTACACACA

CACACATGCACACACACACACACACATACACACACACACACACACTCACACACATACACA

CACACATACACACATACACACACACAAACACA

>AgamP3:3R:13862599:13863800:1_genic

TGTGTGTGTGTATGTGTGTATTTGTGTGTGTATGTGTGTGTGAGTGTGTGTGTGTGTGTG

TGTGTTTGTGTGTTTGTGTGTGTGTGTGTGTGTGTGTGTAGTAGTAGAAAGTGAATGTGC

TTTTTAATGTGTATTTGCAAGTGCGCGGGTTTGTGTCTGAAAAACATAGCTTGCCATGAT

GTCATATTGCGTTGAAATTATTCTTATTATGTGTGTTATCATGTGAAACATTGTGCGTTT

ACACTTGGATAAAAACTAAAGTTTGCATCGACAGCAGCCCTTGTTCCTCGGACGAAAACG

CAGGTGTGCTGTTTCATGAATGTGAATACGACACCGGTGCGAAATGAAAACGCGCACAAT

AGCAATGAACTCGGCATTCCCTCACAGGTGTTTCTCCAGTGCACGCCATTGAAAGGCATG

CGTGCATCTTTGCGTTGAACGTTGTGTGCGCATGGGAAACTTTGTGTGTGCATTGAGCAA

GCGCGCAGTTGTTCTTTTCAATGGGCGTTTTGTTTCATGAGCGCGGCAGTATTCTGTTCT

CGATTTGGAAGGGAAGACGCTTACTCGCGTACTCGTTGATAGTTTTTATCCATGCGATGT

TTTCGCTGCTCGACAACGATGTAATTCATCGCGTATAGAAGTAGAACGCAGGCAGAGCAC

GGGATCAGCCGTGTCCAAGTTTTTAACCAGCGCGTTCTTGACGGTCCAAGCAAGCAATGT

TAGTAACAATGGGTCGAGGTAAACGTTGTACCGATTATCAGCGCCATATGTAAAAGCGAA

TGGCTGCTGCTGGCATTAAGCGCAAAAGGATCGAGTTCGTAATGGAACGATCGCGCACTT

TTGCGGCAAACGCGCTTCGGACAACCGAAACGTGCTTAGACAACTGAAACGCGCTTGGAC

AACCGAAACGCGCAAGTCAACCGGATGTCCTCAGAAGACCACGGCGAAAGAAGACCGTAA

CATTGTTAATATATCGAAAACACACTGATCGCGAGGGCGGTGGTCCTGCTTATCACACCA

AAAACCTAACCCAAAACACAAAAAAGCTACTTACAAATCTATCCGAAACGACCTACTTGT

GAAACAAACACACACATCAATACACATTCAAACATACATCCACACACACACACACACATG

CACACACACATACACACACACACATACACACACATACACACACACACACAAACACAAACA

CA

>AgamP3:3R:33983540:33984801:1_genic

TGTGTTTGTGTGTGTGTATGTGTGTATGTGTGTGTGTATGTGTGTGAGTGTGTGTGTGTG

TGTGTGTGTGAGTGTGTGTGTGTATGTGTGTGTGTGTGTGTGCATGTGTGTGTGTGTGTA

GTAGTAGAAAGAGAGTGTGCTTTTTAATGTGTATTAGCAAGTGCGCGGGTTTGTGTCTGA

AAAACATAGCTTGCCATGATGTCATATTGCGTTGAAATTATTCTTATTATGTGTGTTATC

ATGTGAAACATTGTGCGTTTACACTTGGATAAAAACTAAAGTTTGCATCGACAGCAGCGC

TTGTTCCTCGGACGAAAACGCAGGTGTGCTGTTTCATGAATGTGAATGCGACACCGGTGC

GAAATGGACACGCGCACAATAGCAATGAACTCGGCATTCCCTCACAGGTGTTTCTCCAGT

GCACGCCATTGAAAGGCCTTCGTGCATCTCTGCGTTGCACGTTGTGTGCGAATGGTAAAC

TTTGTGCGTGCATTGAGCTGGCGCGCAGTTATTCTTTTCAATGGGCGTTTTGTTTCATGA

GCGCGGCAGTATTCTTTTCTCGATTTTGAAGGGAAGACGCTTACTCGCGTACTCGTTGAT

AGTTTTTATCCATGCGATGTTTTTGCTGCTCCAAAACGATGTAATTCATCGCGTATAGAA

GTAGAACGCAGGCAGAGCACGGGATCAGCCGTGTCCAAGTTTTTAACTAGTGCGTTCTTG

ACGGTCCAAGCAAGCAATGTTAGTAACAATGGGTCGAGGTAAACGTTGTACCGATCATCA

GCGCCATATGTAAAAGCGAATGGCTGCTGCTGGCATTAAGCGCAAAAGGATCGAGTTCGT

AATGGAACGATCGCGCACTTTTGTGGCAAACGCGCTTCGGACAACCGAAACGTGCTTAGA

CAACTGAAACGCGTTTGGACAACCGAAACGCGCAAGTCAACCGGACGTCCTCAGAAGACC

ACGGCGAAAGAAGACCGTAACATTGTTAATACATCGAAAACACACTGATCGCGAGGGCGG

TGGTCCTGCTTATCACACCAAAAACCTAACCCAAAACACAAAAAAACTACTAACAAAACT

ATCTGAAACGACCTACTTGTGAAACAAACACACACATCAATACACATTCAAACATACATC

CACACACACACACACACACATGCACACACACATATACACACACACACACACACACACACA

CACACACACACACAAACACACATACACACACATACACACACATACACACACACACAAACA

CA

>AgamP3:3R:37504945:37506204:1

TGTGTTTGTGTTTGTGTGTGTGTGTGTATGTGTGTGTATGTGTGTGTGTGTATGTGTGTG

TGCATGTGTGTGTGTGTGTGTGTGGATGTATGTTTGAATGTGTATTGATGTGTGTGTTTG

TTTCACAAGTAGGTCGTTTCGGATAGATTTGTAAGTAGCTTTTTTGTGTTTTGGGTTAGG

TTTTTGGTGTGATAAGCAGGACCACCGCCCTCGCGATCAGTGTGTTTTCGATATATTAAC

AATGTTACGGTCTTCTTTCGCCGTGGTCTTCTGAGGACGTCCGGTTGACTTGCGCGTTTC

GGTTGTCCAAGCGCGTTTCAGTTGTCTAAGCACGTTTCGGTTGTCCGAAGCGCGTTTGCC

ACAAAAGTGCGCGATCGTTCCATTACGAACTCGATCCTTTTGCGCTTAATGCCAGCAGCA

GCCATTCGCTTTTACATATGGCGCTGATAATCGGTACAACGTTTACCTCGACCCATTGTT

ACTAACATTGCTTGCTTGGACCGTCAAGAACGCGCTGGTTAAAAACTTGGACACGGCTGA

TCCCGTGCTCTGCCTGCGTTCTACTTCTATACGCGATGAATTACATCGTTGTCGAGCAGC

GAAAACATCGCATGGATAAAAACTATCAACGAGTACGCGAGTAAGCGTCTTCCCTTCAAA

ATCGAGAACAGAATACTGCCGCGCTCATGAAACAAAACGCCCATTGAAAAGAACAACTGC

GCGCCAGCTCAATGCACGCACAAAGTTTACCATTCGCACACAACGTGCAACGCAGAGATG

CACGAAGGCCTTTCAATGGCGCGCACTGGAGAAACACCTGTGAGGGAATGCCGAGTTCAT

TGCTATTGTGCGCGTGTCCATTTCGCACCAGTGTCGCATTCACATTCATGAAACAGCACA

CCTGCGTTTTCGTCTGAGAAACAAGCGCTGCTGTCGATGCAAACTTTAGTTTTTATCCAA

GTGTAAACGCACAATGTTTCACATGATATCACACATAATAAGAATAATTTCAACGCAATA

TGACATCATGGCAAGCTATGTTTTTCAGACACAAACCCGCGCACTTGCAAATACATATTA

AAAAGCACACTCACTTTCTACTACTACACACACACACATGCACACACACACACACACACA

CACACACACACACACACACACACACACACACACACACACACACACACACACACACACAAA

CACACACACACACTCACACACACACACATACACACACACATACACACATACACACACACA

>AgamP3:3R:46198981:46200254:1

TGTGTTTGTGTGTGTGTATGTGTGTATGTGTGTGTGTATGTGTGTGAGTGTGTGTGTGTG

TGTGTGTGTGTATGTGTGTGTGTGTGTGCATGTGTGTGTGTGTGTAGTAGTAGAAAGAGA

GTGTGCTTTTTAATGTGTATTAGCAAGTGCGCGGGTTAGTGTCTGAAAAACATAGCTTGC

CATGATGTCATATTGCGTTGAAATTATTCTTATTATGTGTGTTATCATGTGAAACATTGT

GCGTTTACCCTTGGATAAAAACTAAAGTTTGCATCGACAGCAGCGCTTGTTCCTCGGACG

AAAACGCAGGTGTGCTGTTTCATGAATGTGAATGCGACACCGGTGCGAAATGGACACGCG

CACAATAGCAATGAACTCGGCATTCCCTCACAGGTGTTTCTCCAGTGCACGCCATTGAAA

GGCCTTCGTGCATCTCTGCGTTGCACGTTGTGTGCGAATGGTAAACTTTGTGCGTGCATT

GAGCTGGCGCGCAGTTATTCTTTTCAATGGGCGTTTTGTTTCATGAGCGCGGCAGTATTC

TTTTCTCGATTTTGAAGGGAAGACGCTTACTCGCGTACTCGTTGATAGTTTTTATCCATG

CGATGTTTTTGCTGCTCCAAAACGATGTAATTCATCGCGTATAGAAGTAGAACGCAGGCA

GAGCACGGGATCAGCCGTGTCCAAGTTTTTAACCAGTGCGTTCTTGACGGTCCAAGCAAG

CAATGTTAGTAACAATGGGTCGAGGTAAACGTTGTACCGATCATCAGCGCCATATGTAAA

CGCGAATGGCTGCTGCTGGCATTAAGCGCAAAAGGATCGAGTTCGTAATGGAACGATCGC

GCACTTTTGTGGCAAACGCGCTTCGGACAACCGAAACGTGCTTAGACAACTGAAACGCGT

TTGGACAACCGAAACGCGCAAGTCAACCGGACGTCCTCAGAAGACCACGGCGAAAGAAGA

CCGTAACATTGTTAATACATCGAAAACACACTGATCGCGAGGGCGGTGGTCCTGCTTATC

ACACCAAAAACCTAACCCAAAACACAAAAAAACTACTTACAAATCTATTCGAAACGACCT

ACTTGTGAAACAAACACACACATCAATACACATTCAAACATACATCCACACACACACACA

CATGCACACACACATACACACACACACACACACACACACACACACACACACACACACACA

CACACACACACACACACACACACACACAAACACACATACACACACATACACACACATACA

CACACACAAACACA

>AgamP3:3R:9530744:9531955:1

TGTGTTTGTGTGTGTGTATGTGTGTATGTGTGTGTGTATGTGTGTGTGAGTGTGTGTGTG

TGTGTGTATGTGTGTGTGTGTGCATGTGTGTGTGTGTGTAGTAGTAGAAAGAGAGTGTGC

TTTTTAATGTGTATTAGCAAGTGCGCGGGTTTGTGTCTGAAAAACATAGCTTGCCATGAT

GTAATATTGCGTTGAAATTATTCTTATTATGTGTGTTATCATGTGAAACATTGTGCGTTT

ACATTTGGATAAAAACTAAAGTTTGCATCGACAGCAGCGCTTGTTCCTCGGACGAAAACG

CAGGTGTGCTGTTTCATGAATGTGAATGCGACACCGGTGCGAAATGGACACGCGCACAAT

AGCAATGAACTCGGCATTCCCTCACAGGTGTTTCTCCAGTGCACGCCATTGAAAGGCCTT

CGTGCATCTCTGCGTTGCACGTTGTGTGCGAATGGTAAACTTTGTGCGTGCATTGAGCTG

GCGCGCAGTTGTTCTTTTCAATGGGCGTTTTGTTTCATGAGCGCGGCAGTATTCTGTTCT

CGATTTTGAAGGGAAGACGCTTACTCGCGTACTCGTTGATAGTTTTTATCCATGCGATGT

TTTCGCTGCTCCAAAACGATGTAATTCATCGCGTATAGAAGTAGAACGCAGGCAGAGCAC

GGGATCAGCCGTGTCCAAGTTTTTAACCAGCGCGTTCTTGACGGTCCAAGCAAGCAATGT

TAGTAACAATGGGTCGAGGTAAACGTTGTACCGATCATCAGCGCCATATGTAAAAGCGAA

TGGCTGCTGCTGGCATTAAGCGCAAAAAAATCGAGTTCGTAATGGAACGATCGCGCACTT

TTGTGGCAAACGCGCTTCGGACAACCGAAACGTGCTTAGACAACTGAAACGCGCTTGGAC

AACCGAAACGCGCAAGTCAACCGGACGTCCTCAGAAGACCACGGCGAAAGAAGACCGTAA

CATTGTTAATTAATCGAAAACACACTGATCGCGAGGGCGGTGGTCCTGCTTATCACACCA

AAAACCTAACCCAAAACACAAAAAAACTACTTACAAATCTATCCGAAACGACCTACTTGT

GAAACAAACACATACATCAATACACATTCAAACATACATCCACACACACACACACACATG

CACACACACATACACACACACACACACACACAAACACACATACACACATATACACACACA

CACACAAACACA

>AgamP3:3R:9841590:9842797:1

TGTGTTTGTGTGTGTGTGTATGTGTGTGTATGTGTGTGTGTGTGTATGTGTGTGTGCATG

TGTGTGTGTGTGGATGTATGTTTGAATGTGTATTGATGTGTGTGTTTGTTTCACAAGTAG

GTCGTTTCGGATAGATTTGTAAGTAGCTTTTTTGTGTTTTGGGTTAGGTTTTTGGTGTGA

TAAGCAGGACCACCGCCCTCGCGATCAGTGTGTTTTCGATATATTAACAATGTTACGGTC

TTCTTTCGCCGTGGTCTTCTGAGGACGTCCGGTTGACTTGCGCGTTTCGGTTGTCCAAGC

GCGTTTCAGTTGTCTAAGCACGTTTCGGTTGTCCGAAGCGCGTTTGCCACAAAAGTGCGC

GATCGTTCCATTACGAACTCGATCCTTTTGCGCTTAATGCCAGCAGCAGCCATTCGCTTT

TACATATGGCGCTGATAATCGGTACAACGTTTACCTCGACCCATTGTTACTAACATTGCT

TGCTTGGACCGTCAAGAACGCGCTGGTTAAAAACTTGGACACGGCTGATCCCGTGCTCTG

CCTGCGTTCTACTTCTATACGCGATGAATTACATCGTTTTGGAGCAGCAAAAACATCGCA

TGGATAAAAACTATCAACGAGTACGCGAGTAAGCGTCTTCCCTTCAAAATCGAGAAAAGA

ATACTGCCGCGCTCATGAAACAAAACGCCCATTGAAAAGAACAACTGAGCACCAGCTCAA

TGCACGCACAAAGTTTACCATTCGCACACAACGTGCAACGCAGAGATGCACGAAGGCCTT

TCAATGGCGTGCACTGGAGAAACACCTGTGAGGGAATGCCGAGTTCATTGCTATTGTGCG

CGTGTCCATTTCGCACCGGTGTCGCATTCACATTCATGAAACAGCACACCTGCGTTTTCG

TCCGAGGAACAAGCGCTGCTGTCGATGCAAACTTTAGTTTTTATCCAAGTGCAAACGCAC

AATGTTTCACATGATAACACACATAATAAGAATAATTTCAACGCCACATGACATCATGGC

AAGCTATGTTTTTCAGACACAAACCCGCGCACTTGCAAATACACATTAAAAAGCACACTC

TCTTTCTACTACTACACACACACACACACATGCACACACACACACACACACACACACAAA

CACACACACACACACACATACACACACACTCACACACACACATACACACATACACACACA

CAAACACA

>AgamP3:X:10765596:10766891:1

TGTGTTTGTGTGTGTGTATGTGTGTATGTGTGTGTGTATGTGTGTGTGTGAGTGTGTGTG

TGTATGTGTGTGTGTGTGCATGTGTGTGTGTGTGTGTAGTAGTAGAAAGAGAGTGTGCTT

TTTAATGTGTATTTGCAAGTGCGCGGGTTTGTGTCTGAAAAACATAGCTTGCCATGATGT

CATATTGCATTGAAATTATTCTTATTATGTGTGTTATCATGTGAAACATTGTGCGTTTAC

ACTTGGATGAAAACTAAAGTTTGCATCGACAGCAGCGCTTGTTCCTCGGACGAAAACGCA

GGTGTGCTGTTTCATGAATGTGAAAGCGACACCGATGCGAAATGGACACGCGCACAATAG

CAATGAACTCGGCATTCCCTTACAGGTGTTTCTCCAGTGCACGCCATAGAAATGCCTGCG

TGCATCTCTGCGTTGCACGTTGTGTGCGAATGGTAAACTTTGTGCGTGCATTGAGCTGGC

GCGCAGTTATTCTTTTCAATGGGCGTTTTGTTTCATGAGCGCGGCAGTATTCTTTTCTCG

ATTTTGAAGGGAAGACGCTTACTCGCGTACTCGTTGATAGTTTTATCCATGCGATGTTTT

CGCTGCTCAAAAACGATGTAATTCATCGCGTATAGAAGTAGAACGCAGGCAGAGCACTGG

ATCAGCCGTGTCCAAGTTTTTAACCAGCGCGTTCTTGACGGTCCAAGCAAGCAATGTTAG

TAACAATGGGTCGAGGTAAAGGTTGTACCGATTATCAGCGCCATATGTAAAAGCGAATGG

CTGCTGCTGGCATTAAGCGCAAAAGGATCGAGTTCGTAATGGAACGATCGCGCACTTTTG

TGGCAAACGCGCTTCGGACAACCGAAACGTGCTTGGACAACTGAAACGCGCTTGGACAAC

CGAAACGCGCAAGTTAACCGGACGTCCTCAGAAGACCACGGCGAAAGAAGACCGTAACAT

TGTTAATATATCGAAAACACACTGATCGCGAGGGCGGTGGTCCTGCTTATCACAACAAAA

ACCTAACCCAAAACACAAAAAAACTACTTACAAATCTATCCGAAACGACCTACTTGTGAA

AAAAACACACACATCAATACACATTCAAAACATTCATCCACACACACACACACACACACA

CACACACACACACACACACACACACACACACACACACACACACACACACACACACACACA

CACACGCACGCACACACACACACACACACACACACATGCACACACACATACACACACACA

CACATACACACACACACACACACAAACACAAACACA

>AgamP3:X:20805691:20807064:1

TGTGTTTGTGTGTGTGTGTATGTGTGTGTATGTGTGTTTGTGTGTGTGTATGTGTGTGTG

TGTGTGTGTGTGTGTGTGTGTGTGTGTGTGTGTGTGTGTGTGTGTGTGTGTGTGTGTGTG

TGTGTGTGTGTGTGTATGTGTGTGTGCATGTGTGTGTGTGTGTGTGGATGTATGTTTGAA

TGTGTATTGATGTGTGTGTTTGTTTCACAAGTAGGTCGTTTCGGATCGATTTGTAAGTAG

TTTTTTTGTGTTTTGGGTTAGGTTTTTGGTGTGATAAGCAGGACCACCGCCCTCGCGATC

AGTGTGTTTTCGATATATTAACAATGTTACGGTCTTCTTTCGCCGTGGTCTTCTGAGGAC

GTCCGGTTGACTTGCGCGTTTCGGTTGTCCAAGCGCGTTTCAGTTGTCTAAGCACGTTTC

GGTTGTCCGAAGCGCGTTTGCCACAAAAGTGCGCGATCGTTCCATTACGAACTCGATCCT

TTTGCGCTTAATGCCAGCAGCAGCCATTCGCTTTTACATATGGCGCTGATGATCGGTACA

ACGTTTACCTCGACCCATTGTTACTAACATTGCTTGCTTGGACCGTCAAGAACGCGCTGG

TTAAAAACATGGACACGGCTGATTCCGTGCTCTGCCTGCGTTCTACTTCTATACGCGATG

AATTACATCGTTTTGGAGCAGCGAAAACATCGCATGGATAAAAACTATCAACGAGTACGC

GAGTAAGCGTCTTCCCTTCAAAATCGAGAAAAGAATACTGCCGCGCTCATGAAACAAAAC

GCCCATTGAAAAGAATAACTGCGCGCCAGCTCAATGCACGCACAAAGTTTACCATTCGCA

CACAACGTGCAACGCAGAGATGCACGAAGGCCTTTCAATGGCGTGCACTGGAGAAACACC

TGTGAGGGAATGCCGAGTTCATTGCTATTGTGCGCGTGTCCATTTCGCACCGGTGTCGCA

TTCACATTCATGAAACAGCACACCTGCGTTTTCGTCCGAGGAACAAGCGCTGCTGTCGAT

GCAAACTTTAGTTTTTATCCAAGTGCAAACGCACAATGTTTCACATGATAACACACATAA

TAAGAATAATTTCAACGCAATATGACATCATGGCAAGCTATGTTTTTCAGACACAAACCC

GCGCACTTGCAAATACACATTAAAAAGCACACTCTCTTTCTACTACTACACACACACACA

CATGCACACACACACACACACACACACATACACACTCACACATACACACTCACACACACA

CTCACACACACATACACACACACATACACACACACACACACACACACACACACACACACA

CACACACACACACACACACACACACACACACACACATACACACACACAAACACA

>AgamP3:X:23396545:23397752:1

TGTGTTTGTGTGTGTGTGTGTGTATGTGTGTGTATGTGTGTTTGTGTGTGTGTGTGTGTA

TGTGTGTGTGCATGTGTGTGTGTGTGTGTGTGTGGATGTATGTTTGAATGTGTATTGATG

TGTGTGTATGTTTCACAAGTAGGTCGTTTCGGATAGATTTGTAAGTAGTTTTTTTGTGTT

TTGGGTTAGGTTTTTGGTGTGATAAGCAGGACCACCGCCCTCGCGATCAGTGTGTTTTCG

ATATATTAACAATGTTACGGTCTTCTTTCGCCGTGGTCTTCTGAGGACGTCCGGTTGACT

TGCGCGTTTCGGTTGTCCAAGCGCGTTTCAGTTGTCTAAGCACGTTTCGGTTGTCCGAAG

CGCGTTTGCCACAAAAGTGCGCGATCGTTCCATTACGAACTCGATCCTTTTGCGCTTAAT

GCCAGCAGCAGCCATTCGCTTTTACATATGGCGCTGATGATCGGTACAACGTTTACCTCG

ACCCATTGTTACTAACATTGCTTGCTTGGACCGTCAAGAACGCGCTGGTTAAAGAGTTGG

ACACGGCTGATCCCGTGCTCTGCCTGCGTTCTACTTCTATACGCGATGAATTACATCGTT

TTGGAGCAGCGAAAACATCGCATGGATAAAAACTATCAACGAGTACGCGAGTAAGCGTCT

TCCCTTCAAAATCGAGAAAAGAATACTGCCGCGCTCATGAAACAAAACGCCCATTGAAAA

GAATAACTGCGCGCCAGCTCAATGCACGCACAAAGTTTACCATTCGCACACAACGTGCAA

CGCAGAGATGCACGAAGGCCTTTCAATGGCGTGCACTGGAGAAACACCTGTGAGGGAATG

CCGAGTTCATTGCTATTGTGCGCGTGTCCATTTCGCACCGGTGTCGCATTCACATTCATG

AAACAGCACACCTGCGTTTTCGTCCGAGGAACAAGCGCTGCTGTCGATGCAAACTTTAGT

TTTTATCCAAGTGTAAACGCACAATGTTTCACATGATAACACACATAATAAGAAAAATTT

CAACGCAATATGACATCATGGCAAGCTATGTTTTTCAGACACAAACCCGCGCACTTGCTA

ATACACATTAAAAAGCACACTCTCTTTCTACTACTACACACACACACACATGCACACACA

CACACATACACACACACACACTCACACACACATACACACACATACACACATACACACACA

CAAACACA

>AgamP3:X:23830531:23831848:1

TGTGTTTGTGTGTGTGTGTATGTGTGTGTATGTGTGTTTGTGTGTCTGTGTGTGTGTGTG

TGTGTGTGTGTGTGTGTGTGTGTGTGTGTGTGTGTGTGTGTGTGTGTGTGTGTGTGTGTG

TGTGTGTGTGTGTGTGTGTGTGTGTGTGTATGTGTGTGTGCATGTGTGTGTGGATGTATG

TTTGAATGTGTATTGATGTGTGTGTTTGTTTCACAAGTAGGTCGTTTCGGATAGATTTGT

AAGTAGTTTTTTTGTGTTTTGGGTTAGGTTTTTGGTGTGATAAGCAGGACCACCGCCCTC

GCGATCAGTGTTTTTTCGATATATTAACAATGTTACGGTCTTCTTTCGCCGTGGTCTTCT

GAGGACGTTCGGTTGACTTGCGCGTTTCGGTTGTCCAAGCGCGTTTCAGTTGTCTAAGCA

CGTTTCGGTTGTCCGAAGCGCGTTTGCCACAAAAGTGCGCGATCGTTCCATTACGAACTC

GATCCTTTTGCGCTTAATGCCAGCAGCAGCCATTCGCTTTTACATATGGCGCTGATGATC

GGTACAACGTTTACCTCGACCCATTGTTACTAACATTGCTTGCTTGGACCGTCAAGAACG

CGCTGCTTAAAAACTTGGACACGGCTGATCCCGTGCTCTGCCTGCGTTCTGTTTCTACAC

GTGATGAATTACATCGTTGTAGAGCAGCAAAAACATCGCATGGATAAAAACTATCAACGA

CTACGCGAGTAAGCGTCTTCCCTTCAAAATCGAGAACAGAATACTGCCGCGCTCATGAAA

CAAAACGCCCATTGAAAAGAATAACTGCGCGCCAGCTCAATGCACGCACAAAGTTTACCA

TTCGCACACAACGTGCAACGCAGAGATGCACGAAGGCCTTTCAATGGCGTGCACTGGAGA

AACACCTGTGAGGGAATGCCGAGTTCATTGCTATTGTGCGCGTGTCCATTTCGCACCGGT

GTCGCATTCACATTCATGAAACAGCACACCTGCGTTTTCGTCCGAGGAACAAGGGCTGCT

GTCGATGCAAACTTTAGTTTTTATCCAAGTGTAAACGCACAATGTTTCACATGATAACAC

ACATAATAAGAATAATTTCAACGCAATATGACATCATGGCAAGCTATGTTTTTCAGACAC

AAACCCGCGCACTTGCAAATACACATTAAAAAGCACACTCTCTTTCTACTACTACACACA

CACACACATGCACACACACACACATGCACACACACACATACACACACACACACACACACA

CACACTCACACACACATACACACACACATACACACACATACACACATACACACACACA

>AgamP3:X:24036297:24037516:1

TGTGTTTGTGTTTGTGTGTGTGTGTATGTGTGTGTATGTGTGTGTGTGTGTGTATGTGTG

TGTGCATGTGTGTGTGTGTGTGGATGTATGTTTGAATGTGTATTGATGTGTGTGTTTGTT

TCACAAGTAGGTCGTTTCGGATAGATTTGTAAGTAGCTTTTTTGTGCTTTGGGTTAGGTT

TTTGGTGTGATAAGCAGGACCACCGCCCTCGCGATCAGTGTGTTTTCGATATATTAACAA

TGTTACGGTCTTCTTTCGCCGTGGTCTTCTGAGGACGTCCGGTTGACTTGCGCGTTTCGG

TTGTCCAAGCGCGTTTCAGTTGTCTAAGCACGTTTCGGTTGTCCGAAGCGCGTTTGCCAC

AAAAGTGCGCGATCGTTCCATTACGAACTCGATCCTTTTGCGCTTAATGCCAGCAGCAGC

CATTCGCTTTTACATATGGCGCTGATAATCGGTACAACGTTTACCTCGACCCATTGTTAC

TAACATTGCTTGCTTGGACCGTCAAGAAAGCGCTGGTTAAAAACTTGGACACGGCTGATC

CCGTGCTCTGCCTGCGTTCTACTTCTATACGCGATGAATAACATCGTTGTCGAGCAGCGA

AAACACCGCATGGAAAAAAACTATCAACGAGTACGCGAGTAAGCGTCTTCCCTTCAAAAT

CGAGAACAGAATACTGCCGCGCTCATGAAACAAAACGCCCATTGAAAAGAACAACTGCGC

GCCAGCTCAATGCACGCACAAAGTTTACCATTCGCACACAACGTGCAACGCAGAGATGCA

CGAAGGCCTTTCAATGGCGTGCACTGGAGAAACACCTGTGAGAGAATGTCGAGTTCATTG

CTATTGTGCGTGTGTCCATTTCGCACCGGTGTCGCATTCACATTCATGAAACAGCAGACC

TGCGTTTTCGTCCGAGGAACAAGGGCTGCTGTCGATGCAAACTTTAGTTTTTATCCAAGT

GTAAACGCACAATGTTTCACATGATAACACACATAATAAGAATAATTTCAACGCAATATG

ACATCATGGCAAGCTATGTTTTTCAGACACAAACCCGCGCACTTGCAAATACACATTAAA

AAGCACACTCACTTTCTACTACTACACACACACACATGCACACACACACACACACACACA

CACACACACAAACACACACACACACACACACACACTCACACACACACATACACACACACA

TACACACATACACACACACA

>AgamP3:X:602672:603847:1

TGTGTTTGTGTGTGTGTATGTGTGTATGTGTGTGTGTGTGTGAGTGTGTGTGTGTATGTG

TGTGTGTGTGCATGTGTGTGTGTGTAGTAGTAGAAAGTGAGTGTGCTTTTGATTGTGTAT

TTGCAAGTGCGCGGGTTTGTGTCTGAAAAACATAGCTTGCCATGATGTCATATTGCGTTG

AAATTATTCTTATTATGTGTGTTATCATGTGAAACATTGTGCGTTTGCGCTTGGATAAAA

ACTAAAGTTTGCATCGACAGCAGCCCTTGTTCCTCGGACGAAAACGCAGGTGTGCTGTTT

CATGAATGTGAATGCGACACCGGTGCAAAATGGACACGCGCACAATAGCAATGAACTCGG

CATTCCCTCACAGGTGTTTCTCCAGTGCACGCCATTGAAAGGCCTTCGTGCATCTCTGCG

TTGCACGTTGTGTGCGAATGGTAAACTTTGTGCGTGCATTGAGCTGGCGCGCAGTTGTTC

TTTTCAATGGGCGTTTTGTTTCATGAGCGCGGCAGTATTCTGTTCTCGATTTTGAAGGGA

AGACGCTTACTCGCGTACTCAATGATTGTTTTTATCCATGCGATGTTTTCACTGCTCGAC

AACGATGTAATTCATCGCGTATAGAAGTAGAACGCAGGCAGAGCACGGGATCAGCCGTGT

CCAAGTTTTTAACCAGCGCGTTCTTGACGGTCCAAGCAAGCAATGTTAGTAACAATGGGT

CAAGGTAAACGTTGTACCGATTATCAGCGCCATATGTAAAAGCGAATGGCTGCTGCTGAC

ATTAAGCGCAAAAGGATCGAGTTCGTAATGGAACGATCGCGCACTTTTGTGGCAAACGCG

CTTCGGACAACCGAAACGTGCTTAGACAACTGAAACGCGCTTGGACAACCGAAACGCGCA

AGTCAACCGGACGTCCTCAGAAGACCACGGCGAAAGAAGACCGTAACATTGTTAATATAT

CGAAAACACACTGATCGCGAGGGCGGTGGTCCTGCTTATCACACCAAAAACCTAACCCAA

AACACAAAAAAGCTACTTACAAATCTATCCGAAACGACCTACTTGTGAAACAAACAAACA

CATCAATACACATTCAAACATACATCCACACACACACACATGCATACACACATACACACA

TACACACACATACATACACACACAAACACAAACACA

>AgamP3:X:8376219:8377420:1

TGTGTTTGTGTGTGTGTATGTGTGTATGTGTGTGTGTATGTGTGTGTGTGAGTGTGTGTG

TATGTGTGTGTGTGTGCATGTGTGTGTGTGTGTAGTAGTAGAAAGAGAGTGTGCTTTTTA

ATGTGTATTTGCAAGTGCGCGGGTTTGTGTCTGAAAAACATAGCTTGCCATGATGTCATA

TTGCGTTGAAATTATTCTTATTATGTGTGTTATCATGTGAAACATTGTGCGTTTACACTT

GGATAAAAACTAAAGTTTGCATCGACAGCAGCGCTTGTTCCTCGGACGAAAACGCAGGTG

TGCTGTTTCATGAATGTGAACGCGACACCGGTGCGAAATGGACACGCGCACAATAGCAAT

GAACTCGGCATTCCCTCACAGGTGTTTCTCCAGTGCACGCCATTGAAAGGCCTTCGTGCA

TCTCTGCGTTGCACGTTGTGTGCAAATGGTAAACTTTGTGCGTGCATTGAGCTGGCGCGC

AGTTATTCTTTTCAATGGGCGTTTTGTTTCATGAGCGCGGCAGTATTCTTTTCTCGATTT

TTAAGGGAAGACGCTTATTCGCGTACTCGTTGATAGTTTTTATCCATGCGATGTTTTCGC

TGCTCCAAAACGATGTAATTCATCGCGTATAGAAGTATAACGCAGGCAGAGCACGGGATC

AGCCGTGTCCAAGTTTTTAACCAGCGCGTTCTTGACGGTCCAAGCAAGCAATATTAGTAA

CAATAGGTCGAGGTAAACGTTGTACCGATTATCAGCGCCATATGTAAAAGCGAATGGCTG

CTGCTGGCATTAAGCACAAAAGGATCGAGTTCGTAATGGAACGATCGCGCACTTTTGTGG

CAAATGCGCTTCGGGCAACCGAAACGTGCTTAGACAACTGAAACGCGCTTGGACAACCGA

AACGCGCAAGTCAACCGGACGTCCTCAGAAGACCACGTCGAAAGAAGACCGTAACATTGT

TAATATATCGAAAACACACTGATCGCGAGGGCGGTGGTCCTGCTTATCACACCAAAAACC

TAACCCAAAACACAAAAAAACTACTTACAAATCTATCCGAAACGACCTACTTGTGAAACA

AAAACACACATCAATACACATTCAAACATACATCCACACACACACACACACATGCACACA

CACATACACACACACACACACACACACACACACACACACACACACACACACACATACACA

CA

>AgamP3:X:9719721:9720789:1

AGAGAGAGAGAGAGAGAGAGCGAGAGAGTGGGAATGATATCGAAAAAAAGAGAGAAATGA

CATATTACAGCCTTATTACCACCAATTAACATACGCGTAAAGCCATAAAAACTTGATGAT

GACACGGCCACGCCGAGCCGTACGGTGGTCACACACTTTGCACACCCCCGGCTGCCAAAC

CCCCCCCCCCCCCTCCACGAGAGTTCGGCGATTCCAGTTCCATCGTCGGGCGGCACAGGC

CACCGCCACGTTGGTTGGCAGGGTGCCAAAAACTTTGCCGTTTTGCGAATCGCTTACCGC

AGCTATTTTACAGTTTGCCTGCTGCAAACAAACCAATTAGACAACTTCCTCGACAGAGCA

CCCAAGGGACAGGAGGGGGAGACCTATGGCACAACTCTCAACCATCGGTGGTGTTATTTT

CAGCAGCGGGGGGATAGATAGCAGGAAGCAGTTTAGCTGAAGAAATCTCTCGACCTAATC

GTGGCAAAGTAGCCCGACAAGTCCCCATGGTAGAGGGCCGTCGCAGGACACATACCACAC

ACCGCACAAAAGGGAAACCAAAGAGTGATTCATATAAATAGATAATCTCAACATACACAC

TGCTTCGCCGTTGTGTGCTAAAATAACAATTTGTGTCCATTACAAGGCAGCCGGCCAAGT

GGGAGTGAGTGAGAGCTCGCTTAGCTTCCTCCTCGCAATACACGGGGCGCTTCCAGACGA

GCAACTTTTACGGTCGTACAATGTACCTCGCCTCGGAGTACTCCAGCTCTCCGGTTCTTC

GCTCCTGCTCTACCACTCTCGCAAACTCCCGCAGTAGGAGGAAGATTGAGTGGATGATTT

TAAGCTAGCTCCACACGCTCCACAAGCAAATTGCCTCATTCGCACGCTCATTGCCCGGAA

CAAACCTCTGCTGGCAAATTTACGGCAAACCGACAATGTCCCTAGGATCTACTCACCACC

CATCCATCCCCGTGTCTAATACACCGATACTGCCGGGAACCAAGGTCAGAGCAACCAAGG

TGCCAACTTGTGAAACCTTTCCCTGGATTCACACACACACACACACACA

**C. Copies of *D. melanogaster* mSD: (TC)n~1190bp(ATT)n**

>2L:21489756,21490994

tctctctctctctctctctctctttcaccctccacgattgctatataagtaggtagcaaa

tgctctgatcgtttattgtgttttcaaacgtgaagtagtgaacgtgaactttagtgaaac

ccaaatcggagatggctcgtaccaagcaaactgctcgcaaatcgactggtggaaaggcgc

cacgcaaacaactggctactaaggccgctcgcaagagtgctccagccaccggaggtgtga

agaagccccaccgctatcgccctggaaccgtggccttgcgtgaaattcgtcgctaccaaa

agagcaccgagcttctaatccgcaagctgcctttccagcgtctggtgcgtgaaatcgctc

aggactttaagacggacttgcgattccagagctcggcggttatggctctgcaggaagcta

gcgaagcctacctggttggtctcttcgaagataccaacttgtgtgccattcatgccaagc

gtgtcaccataatgcccaaagacatccagttagcgcgacgcattcgcggcgagcgtgctt

aagctgacacggcattaacttgcagataaagcgctagcgtactctataatcggtcctttt

caggaccacaaaccagattcaatgagataaaattttctgttgccgactatttataacttt

aaaaaaaataagaacaaaattcatattctattatttatggcgcaaacggtactgggtctt

aaatcatatgtaaaaatactaattctgccagagaaggaataaaaataatcttattttaat

tgtcagctcaacatttattaaattaaagaagaggttaatacaaaaatatatatttttatt

tgttctttgtgcgaacatcctttaaagcagtgaaagtgtcgtgcggggcaagggactctg

aaccttaaacatctaaaaaaaaaatctgaattctgtgtaagacagtttgaaattaatgaa

attacattgatgacggcaatatttataaaataacagaaaataataaaataaaactagcta

ttttatattttttccatgtgttaactgaagaatgtgttattattgaagaggtcgtacggg

acaattgacactgtcccttcaaacgtctgtaaaaaataaaacctatgtaaaattcagcac

ggaaattggctaattttgttgcggaatgtaatatatattacataataaaggataatacaa

aaattgtttctttttattttttatttgatttatttattt

>2L:21494801,21496039

tctctctctctctctctctctctttcaccctccacgattgctatataagtaggtagcaaa

tgctctgatcgtttattgtgttttcaaacgtgaagtagtgaacgtgaactttagtgaaac

ccaaatcggagatggctcgtaccaagcaaactgctcgcaaatcgactggtggaaaggcgc

cacgcaaacaactggctactaaggccgctcgcaagagtgctccagccaccggaggtgtga

agaagccccaccgctatcgccctggaaccgtggccttgcgtgaaattcgtcgctaccaaa

agagcaccgagcttctaatccgcaagctgcctttccagcgtctggtgcgtgaaatcgctc

aggactttaagacggacttgcgattccagagctcggcggttatggctctgcaggaagcta

gcgaagcctacctggttggtctcttcgaagataccaacttgtgtgccattcatgccaagc

gtgtcaccataatgcccaaagacatccagttagcgcgacgcattcgcggcgagcgtgctt

aagctgacacggcattaacttgcagataaagcgctagcgtactctataatcggtcctttt

caggaccacaaaccagattcaatgagataaaattttctgttgccgactatttataacttt

aaaaaaaataagaacaaaattcatattctattatttatggcgcaaacggtactgggtctt

aaatcatatgtaaaaatactaattctgccagagaaggaataaaaataatcttattttaat

tgtcagctcaacatttattaaattaaagaagaggttaatacaaaaatatatatttttatt

tgttctttgtgcgaacatcctttaaagcagtgaaagtgtcgtgcggggcaagggactctg

aaccttaaacatctaaaaaaaaaatctgaattctgtgtaagacagtttgaaattaatgaa

attacattgatgacggcaatatttataaaataacagaaaataataaaataaaactagcta

ttttatattttttccatgtgttaactgaagaatgtgttattattgaagaggtcgtacggg

acaattgacactgtcccttcaaacgtctgtaaaaaataaaacctatgtaaaattcagcac

ggaaattggctaattttgttgcggaatgtaatatatattacataataaaggataatacaa

aaattgtttctttttattttttatttgatttatttattt

>2L:21504899,21506137

tctctctctctctctctctctctttcaccctccacgattgctatataagtaggtagcaaa

tgctctgatcgtttattgtgttttcaaacgtgaagtagtgaacgtgaactttagtgaaac

ccaaatcggagatggctcgtaccaagcaaactgctcgcaaatcgactggtggaaaggcgc

cacgcaaacaactggctactaaggccgctcgcaagagtgctccagccaccggaggtgtga

agaagccccaccgctatcgccctggaaccgtggccttgcgtgaaattcgtcgctaccaaa

agagcaccgagcttctaatccgcaagctgcctttccagcgtctggtgcgtgaaatcgctc

aggactttaagacggacttgcgattccagagctcggcggttatggctctgcaggaagcta

gcgaagcctacctggttggtctcttcgaagataccaacttgtgtgccattcatgccaagc

gtgtcaccataatgcccaaagacatccagttagcgcgacgcattcgcggcgagcgtgctt

aagctgacacggcattaacttgcagataaagcgctagcgtactctataatcggtcctttt

caggaccacaaaccagattcaatgagataaaattttctgttgccgactatttataacttt

aaaaaaaataagaacaaaattcatattctattatttatggcgcaaacggtactgggtctt

aaatcatatgtaaaaatactaattctgccagagaaggaataaaaataatcttattttaat

tgtcagctcaacatttattaaattaaagaagaggttaatacaaaaatatatatttttatt

tgttctttgtgcgaacatcctttaaagcagtgaaagtgtcgtgcggggcaagggactctg

aaccttaaacatctaaaaaaaaaatctgaattctgtgtaagacagtttgaaattaatgaa

attacattgatgacggcaatatttataaaataacagaaaataataaaataaaactagcta

ttttatattttttccatgtgttaactgaagaatgtgttattattgaagaggtcgtacggg

acaattgacactgtcccttcaaacgtctgtaaaaaataaaacctatgtaaaattcagcac

ggaaattggctaattttgttgcggaatgtaatatatattacataataaaggataatacaa

aaattgtttctttttattttttatttgatttatttattt

>2L:21509944,21511182

tctctctctctctctctctctctttcaccctccacgattgctatataagtaggtagcaaa

tgctctgatcgtttattgtgttttcaaacgtgaagtagtgaacgtgaactttagtgaaac

ccaaatcggagatggctcgtaccaagcaaactgctcgcaaatcgactggtggaaaggcgc

cacgcaaacaactggctactaaggccgctcgcaagagtgctccagccaccggaggtgtga

agaagccccaccgctatcgccctggaaccgtggccttgcgtgaaattcgtcgctaccaaa

agagcaccgagcttctaatccgcaagctgcctttccagcgtctggtgcgtgaaatcgctc

aggactttaagacggacttgcgattccagagctcggcggttatggctctgcaggaagcta

gcgaagcctacctggttggtctcttcgaagataccaacttgtgtgccattcatgccaagc

gtgtcaccataatgcccaaagacatccagttagcgcgacgcattcgcggcgagcgtgctt

aagctgacacggcattaacttgcagataaagcgctagcgtactctataatcggtcctttt

caggaccacaaaccagattcaatgagataaaattttctgttgccgactatttataacttt

aaaaaaaataagaacaaaattcatattctattatttatggcgcaaacggtactgggtctt

aaatcatatgtaaaaatactaattctgccagagaaggaataaaaataatcttattttaat

tgtcagctcaacatttattaaattaaagaagaggttaatacaaaaatatatatttttatt

tgttctttgtgcgaacatcctttaaagcagtgaaagtgtcgtgcggggcaagggactctg

aaccttaaacatctaaaaaaaaaatctgaattctgtgtaagacagtttgaaattaatgaa

attacattgatgacggcaatatttataaaataacagaaaataataaaataaaactagcta

ttttatattttttccatgtgttaactgaagaatgtgttattattgaagaggtcgtacggg

acaagtgacactgtcccttcaaacgtctgtaaaaaataaaacctatgtaaaattcagcac

ggaaattggctaattttgttgcggaatgtaatatatattacataataaaggataatacaa

aaattgtttctttttattttttatttgatttatttattt

>2L:21514988,21516226

tctctctctctctctctctctctttcaccctccacgattgctatataagtaggtagcaaa

tgctctgatcgtttattgtgttttcaaacgtgaagtagtgaacgtgaactttagtgaaac

ccaaatcggagatggctcgtaccaagcaaactgctcgcaaatcgactggtggaaaggcgc

cacgcaaacaactggctactaaggccgctcgcaagagtgctccagccaccggaggtgtga

agaagccccaccgctatcgccctggaaccgtggccttgcgtgaaattcgtcgctaccaaa

agagcaccgagcttctaatccgcaagctgcctttccagcgtctggtgcgtgaaatcgctc

aggactttaagacggacttgcgattccagagctcggcggttatggctctgcaggaagcta

gcgaagcctacctggttggtctcttcgaagataccaacttgtgtgccattcatgccaagc

gtgtcaccataatgcccaaagacatccagttagcgcgacgcattcgcggcgagcgtgctt

aagctgacacggcattaacttgcagataaagcgctagcgtactctataatcggtcctttt

caggaccacaaaccagattcaatgagataaaattttctgttgccgactatttataacttt

aaaaaaaataagaacaaaattcatattctattatttatggcgcaaacggtactgggtctt

aaatcatatgtaaaaatactaattctgccagagaaggaataaaaataatcttattttaat

tgtcagctcaacatttattaaattaaagaagaggttaatacaaaaatatatatttttatt

tgttctttgtgcgaacatcctttaaagcagtgaaagtgtcgtgcggggcaagggactctg

aaccttaaacatctaaaaaaaaaatctgaattctgtgtaagacagtttgaaattaatgaa

attacattgatgacggcaatatttataaaataacagaaaataataaaataaaactagcta

ttttatattttttccatgtgttaactgaagaatgtgttattattgaagaggtcgtacggg

acaattgacactgtcccttcaaacgtctgtaaaaaataaaacctatgtaaaattcagcac

ggaaattggctaattttgttgcggaatgtaatatatattacataataaaggataatacaa

aaattgtttctttttattttttatttgatttatttattt

>2L:21534562,21535800

tctctctctctctctctctctctttcaccctccacgattgctatataagtaggtagcaaa

tgctctgatcgtttattgtgttttcaaacgtgaagtagtgaacgtgaactttagtgaaac

ccaaatcggagatggctcgtaccaagcaaactgctcgcaaatcgactggtggaaaggcgc

cacgcaaacaactggctactaaggccgctcgcaagagtgctccagccaccggaggtgtga

agaagccccaccgctatcgccctggaaccgtggccttgcgtgaaattcgtcgctaccaaa

agagcaccgagcttctaatccgcaagctgcctttccagcgtctggtgcgtgaaatcgctc

aggactttaagacggacttgcgattccagagctcggcggttatggctctgcaggaagcta

gcgaagcctacctggttggtctcttcgaagataccaacttgtgtgccattcatgccaagc

gtgtcaccataatgcccaaagacatccagttagcgcgacgcattcgcggcgagcgtgctt

aagctgacacggcattaacttgcagataaagcgctagcgtactctataatcggtcctttt

caggaccacaaaccagattcaatgagataaaattttctgttgccgactatttataacttt

aaaaaaaataagaacaaaattcatattctattatttatggcgcaaacggtactgggtctt

aaatcatatgtaaaaatactaattctgccagagaaggaataaaaataatcttattttaat

tgtcagctcaacatttattaaattaaagaagaggttaatacaaaaatatatatttttatt

tgttctttgtgcgaacatcctttaaagcagtgaaagtgtcgtgcggggcaagggactctg

aaccttaaacatctaaaaaaaaaatctgaattctgtgtaagacagtttgaaattaattaa

attacattgatgacggcaatatttataaaataacagaaaataataaaataaaactagcta

ttttatattttttccatgtgttaactgaagaatgtgttattattgaagaggtcgtacggg

acaattgacactgtcccttcaaacgtctgtaaaaaataaaacctatgtaaaattcagcac

ggaaattggctaattttgttgcggaatgtaatatatattacataataaaggataatacaa

aaattgtttctttttattttttatttgatttatttattt

>2L:21541081,21542317

tctctctctctctctctctctttcaccgtccacgattgctatataagtaggtagcaaatg

ctctgatcgtttattgtgttttcaaacgtgaagtagtgaacgtgaactttagtgaaaccc

aaatcggagatggctcgtaccaagcaaactgctcgcaaatcgactggtggaaaggcgcca

cgcaaacaactggctactaaggccgctcgcaagagtgctccagccaccggaggtgtgaag

aagccccaccgctatcgccctggaaccgtggccttgcgtgaaattcgtcgctaccaaaag

agcaccgagcttctaatccgcaagctgcctttccagcgtctggtgcgtgaaatcgctcag

gactttaagacggacttgcgattccagagctcggcggttatggctctgcaggaagctagc

gaagcctacctggttggtctcttcgaagataccaacttgtgtgccattcatgccaagcgt

gtcaccataatgcccaaagacatccagttagcgcgacgcattcgcggcgagcgtgcttaa

gctgacacggcattaacttgcagataaagcgctagcgtactctataatcggtccttttca

ggaccacaaaccagattcaatgagataaaattttctgttgccgactatttataacttaaa

aaaaaataagaacaaaattcatattctattatttatggcgcaaacggtattgggtcttaa

atcatatgtaaaaatactaattctgccagagaaggaataaaaataatcttattttaattg

tcagctcaacatttattaaattaaagaagaggttaatacaaaaatatatatttttatttg

ttctttgtgcgaacatcctttaaagcagtgaaagtgtcgtgcggggcaagggactctgaa

ccttaaacatctaaaaaaaaaatctgaattctgtgtaagacagtttgaaattaattaaat

tacattgatgacggcaatatttataaaataacagaaaataataaaataaaactagctatt

ttatattttttccatgtgttaactgaagaatgtgttattattgaagaggtcgtacgggac

aagtgacactgtcccttcaaacgtctgtaaaaaataaaacctatgtaaaattcagcacgg

aaattggctaattttgttgcggaatgtaatatatattacataataaaggataatacaaaa

attgtttctttttattttttatttgatttatttattt
