# Supplementary material for: Association of microsatellite pairs with segmental duplications in insect genomes
Source: BMC Genomics. 2013 Dec 21;14:907. doi: 10.1186/1471-2164-14-907 (PMC3878106; doi:10.1186/1471-2164-14-907)
Supplement: Additional file 9 — The top five ranking gene ontology (GO) terms of genes associated with rMPs in selected species. [file 1471-2164-14-907-S9.docx]

Functional prediction of genes associated with rMPs.

A). The top five ranking gene ontology (GO) terms associated with genes located in rMP regions. The annotation of only selected species is shown here.

| ***Top 5 ranking GO Term Name*** | ***No of genes*** |
| --- | --- |
| **Aaeg** |  |
| protein binding | 68 |
| membrane | 40 |
| integral to membrane | 35 |
| intracellular | 28 |
| nucleotide binding | 27 |
| **Cqui** |  |
| nucleus | 30 |
| DNA binding | 28 |
| integral to membrane | 23 |
| zinc ion binding | 20 |
| nucleotide binding | 19 |
| **Agam** |  |
| protein binding | 168 |
| intracellular | 96 |
| membrane | 91 |
| integral to membrane | 87 |
| nucleotide binding | 83 |
| **Dmel** |  |
| nucleus | 431 |
| protein binding | 245 |
| zinc ion binding | 219 |
| DNA binding | 184 |
| integral to membrane | 177 |
| **Amel** |  |
| protein binding | 18 |
| regulation of transcription, DNA-dependent | 9 |
| sequence-specific DNA binding transcription factor activity | 9 |
| nucleus | 7 |
| sequence-specific DNA binding | 7 |
| **Tcas** |  |
| protein binding | 5 |
| integral to membrane | 4 |
| membrane | 4 |
| cytoplasm | 2 |
| intracellular | 2 |

B). A select list of *A. aegypti* genes (conserved hypothetical protein genes excluded) those are associated the GO term ‘protein binding’. These are subset of genes those are identified in rMP regions in the genome.

| Gene_ID | Gene_Description |
| --- | --- |
| AAEL000242 | voltage-gated potassium channel |
| AAEL000707 | cell cycle control protein cwf22 |
| AAEL001127 | defective proboscis extension response, putative |
| AAEL001227 | sidestep protein |
| AAEL001874 | cop9 complex subunit |
| AAEL002317 | Insulin-like receptor Precursor (MIR)(EC 2.7.10.1) |
| AAEL003391 | tankyrase |
| AAEL005284 | receptor tyrosine phosphatase type r2a |
| AAEL005519 | synaptotagmin-14 |
| AAEL005661 | defective proboscis extension response, putative |
| AAEL006082 | transcription initiation factor TFIID subunit 1 |
| AAEL006261 | defective proboscis extension response, putative |
| AAEL006939 | smaug protein |
| AAEL007288 | dynamin |
| AAEL007544 | serine/threonine-protein kinase chk2 (cds1) |
| AAEL008058 | liprin alpha (lar-interacting protein alpha) (synapse defective protein 2) |
| AAEL008236 | sidestep protein |
| AAEL008426 | broad-complex core-protein |
| AAEL008914 | PHD finger protein |
| AAEL009458 | syntaxin 4, |
| AAEL009812 | type II collagen, putative |
| AAEL010328 | cAMP-dependent rap1 guanine-nucleotide exchange factor |
| AAEL011480 | tpr repeat nuclear phosphoprotein |
| AAEL012493 | attractin |
| AAEL012730 | dbl |
| AAEL013063 | autophagy related gene |
| AAEL013510 | smaug protein |
| AAEL013539 | SH2/SH3 adaptor protein |
| AAEL013882 | tkr |
