# Supplementary material for: Association of microsatellite pairs with segmental duplications in insect genomes
Source: BMC Genomics. 2013 Dec 21;14:907. doi: 10.1186/1471-2164-14-907 (PMC3878106; doi:10.1186/1471-2164-14-907)
Supplement: Additional file 12 — Association of repetitive paired microsatellites and mSDs with transposable elements of D. melanogaster . [file 1471-2164-14-907-S12.docx]

Association of repetitive paired microsatellites and mSDs with transposable elements of *D. melanogaster*. The repeat family # is shown in the first column corresponds to groups of rMPs listed in Additional File 2. The family # 28 and #49 represent segmental duplications associated with different TEs.

| Repeat family # | Sequence & Inter-SSR distance | Transposable element |
| --- | --- | --- |
| 5 | (T)n__568 bp__(TTA)n | FBti0019601 |
|  |  | FBti0019624 |
|  |  | FBti0019718 |
|  |  | FBti0019757 |
|  |  | FBti0019760 |
|  |  | FBti0019974 |
|  |  | FBti0020306 |
|  |  | FBti0020381 |
|  |  | FBti0020390 |
|  |  | FBti0059649 |
|  |  | FBti0059650 |
|  |  | FBti0059699 |
| 11 | (T)n__68 bp__(T)n | FBti0059671 |
|  |  | FBti0059739 |
|  |  | FBti0060199 |
|  |  | FBti0060200 |
| 12 | (T)n__171 bp__(T)n | FBti0018862 |
|  |  | FBti0019053 |
|  |  | FBti0019354 |
|  |  | FBti0019563 |
|  |  | FBti0020090 |
| 19 | (TGC)n__65 bp__(T)n | FBti0059692 |
|  |  | FBti0063956 |
| 28 | (TATT)n__4855 bp__(TATT)n | FBti0018969 |
|  |  | FBti0019059 |
|  |  | FBti0019136 |
|  |  | FBti0019151 |
|  |  | FBti0019193 |
|  |  | FBti0019364 |
|  |  | FBti0020021 |
| 41 | (TAAAG)n__234 bp__(A)n | FBti0102100 |
|  |  | FBti0102101 |
|  |  | FBti0102102 |
|  |  | FBti0102103 |
| 49 | (CT)n__6995 bp__(CT)n | FBti0018965 |
|  |  | FBti0019096 |
|  |  | FBti0019145 |
|  |  | FBti0019152 |
|  |  | FBti0019210 |
|  |  | FBti0019214 |
|  |  | FBti0019403 |
|  |  | FBti0019471 |
| 56 | (ATT)n__75 bp__(T)n | FBti0020134 |
|  |  | FBti0059781 |
|  |  | FBti0060199 |
| 62 | (A)n__9 bp__(TAT)n | FBti0019130 |
|  |  | FBti0019143 |
|  |  | FBti0019185 |
|  |  | FBti0019338 |
|  |  | FBti0019545 |
|  |  | FBti0019635 |
| 63 | (A)n__10 bp__(TAT)n | FBti0018921 |
|  |  | FBti0019300 |
|  |  | FBti0019620 |
|  |  | FBti0059768 |
| 64 | (ATACAT)n__256 bp__(TTAAA)n | FBti0059704 |
|  |  | FBti0059758 |
| 66 | (ATACA)n__28 bp__(ATACA)n | FBti0063660 |
|  |  | FBti0063720 |
| 68 | (ATA)n__7 bp__(AAAT)n | FBti0018861 |
|  |  | FBti0019563 |
|  |  | FBti0020019 |
|  |  | FBti0020090 |
| 82 | (A)n__65 bp__(CAG)n | FBti0019015 |
|  |  | FBti0019018 |
|  |  | FBti0019019 |
|  |  | FBti0019025 |
|  |  | FBti0019026 |
|  |  | FBti0019027 |
|  |  | FBti0019028 |
|  |  | FBti0019030 |
|  |  | FBti0019031 |
|  |  | FBti0019051 |
|  |  | FBti0019083 |
|  |  | FBti0019098 |
|  |  | FBti0019126 |
|  |  | FBti0019131 |
|  |  | FBti0019138 |
|  |  | FBti0019140 |
|  |  | FBti0019148 |
|  |  | FBti0019173 |
|  |  | FBti0019175 |
|  |  | FBti0019196 |
|  |  | FBti0019205 |
|  |  | FBti0019231 |
|  |  | FBti0019238 |
|  |  | FBti0019337 |
|  |  | FBti0019339 |
|  |  | FBti0019374 |
|  |  | FBti0019416 |
|  |  | FBti0019421 |
|  |  | FBti0019432 |
|  |  | FBti0019436 |
|  |  | FBti0019458 |
|  |  | FBti0019461 |
|  |  | FBti0019463 |
|  |  | FBti0019532 |
|  |  | FBti0019539 |
|  |  | FBti0019544 |
|  |  | FBti0019553 |
|  |  | FBti0019556 |
|  |  | FBti0019558 |
|  |  | FBti0019608 |
|  |  | FBti0019619 |
|  |  | FBti0019630 |
|  |  | FBti0019838 |
|  |  | FBti0020014 |
|  |  | FBti0020022 |
|  |  | FBti0020038 |
|  |  | FBti0020063 |
|  |  | FBti0020085 |
|  |  | FBti0020129 |
|  |  | FBti0020154 |
|  |  | FBti0020166 |
|  |  | FBti0059691 |
|  |  | FBti0059697 |
|  |  | FBti0059707 |
|  |  | FBti0059710 |
|  |  | FBti0059714 |
|  |  | FBti0059726 |
|  |  | FBti0059737 |
|  |  | FBti0059762 |
|  |  | FBti0059768 |
|  |  | FBti0059769 |
|  |  | FBti0059777 |
|  |  | FBti0059830 |
|  |  | FBti0060681 |
|  |  | FBti0062208 |
|  |  | FBti0062794 |
| 87 | (A)n__75 bp__(AAT)n | FBti0019000 |
|  |  | FBti0019001 |
|  |  | FBti0019002 |
|  |  | FBti0019347 |
|  |  | FBti0019447 |
|  |  | FBti0019548 |
|  |  | FBti0019987 |
|  |  | FBti0020025 |
|  |  | FBti0020158 |
| 88 | (AAAATA)n__138 bp__(TATGTT)n | FBti0018871 |
|  |  | FBti0018872 |
|  |  | FBti0018873 |
|  |  | FBti0018874 |
|  |  | FBti0019086 |
|  |  | FBti0019380 |
|  |  | FBti0019383 |
|  |  | FBti0019390 |
|  |  | FBti0019462 |
|  |  | FBti0019521 |
|  |  | FBti0019524 |
|  |  | FBti0019531 |
|  |  | FBti0019566 |
|  |  | FBti0019582 |
|  |  | FBti0019614 |
|  |  | FBti0019704 |
|  |  | FBti0020013 |
|  |  | FBti0020015 |
|  |  | FBti0020033 |
|  |  | FBti0020072 |
|  |  | FBti0020082 |
|  |  | FBti0020094 |
|  |  | FBti0020132 |
|  |  | FBti0020165 |
|  |  | FBti0020186 |
|  |  | FBti0059698 |
